# Sustainability and Techno-Economic Assessment of Batch and Flow Chemistry in Seven Industrial Pharmaceutical Processes

**DOI:** 10.1021/acssuschemeng.4c09289

**Published:** 2025-02-14

**Authors:** Mert Can Ince, Brahim Benyahia, Gianvito Vilé

**Affiliations:** † Department of Chemistry, Materials, and Chemical Engineering “Giulio Natta”, Politecnico di Milano, Piazza Leonardo da Vinci 32, 20133 Milano, Italy; ‡ Chemical Engineering Department, Loughborough University, Epinal Way, LE11 3TU Loughborough, Leicestershire, U.K.

**Keywords:** continuous-flow synthesis, API manufacturing, green chemistry, life-cycle assessment, techno-economic analysis

## Abstract

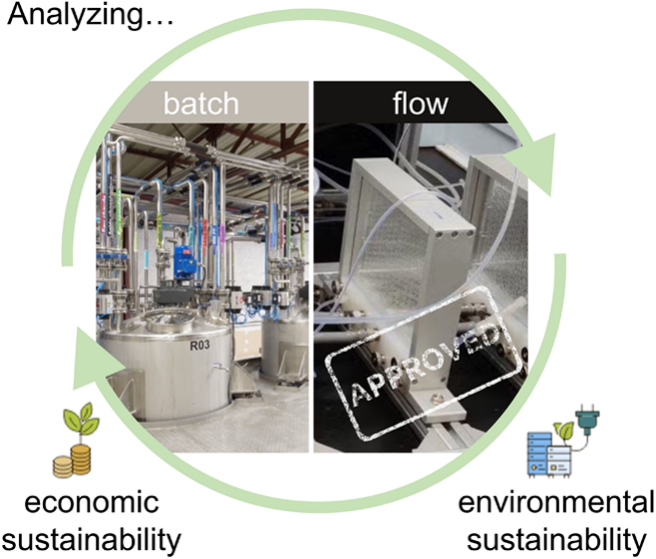

The synthesis of active pharmaceutical ingredients (APIs) is commonly perceived as more efficient when performed using continuous-flow methods, whereas batch processes are often seen as less favorable due to their limitations in yield, heat and mass transfer, and safety. This perception largely stems from existing studies that focus on green metrics such as the E-factor and yield. However, a comprehensive comparison of batch and flow processes through full techno-economic analyses (TEA) and life-cycle assessments (LCA) remains underexplored, leaving key aspects of their environmental and economic impacts inadequately assessed. This work addresses this gap by presenting a detailed comparison of batch and flow syntheses of seven industrially relevant APIs, including amitriptyline hydrochloride, tamoxifen, zolpidem, rufinamide, artesunate, ibuprofen, and phenibut. Eleven environmental impact categories within the framework of nine planetary boundaries were assessed, and the study also included an evaluation of capital and operating costs for both production methods. The results demonstrated that, on average, continuous-flow processes are significantly more sustainable with improvements in energy efficiency, water consumption, and waste reduction. Flow processes also show a marked reduction in carbon emissions and up to a 97% reduction in energy consumption, highlighting their potential for greener API manufacturing. Despite these advantages, the study identified areas where the continuous-flow technology requires further development. Specifically, manufacturing certain APIs in flow show lower-than-average improvements in operating expenditure and land system changes, the latter being directly correlated with the consumption of organic solvents, that can be comparable to or even higher than in batch. These challenges highlight the need for further optimization of flow processes to fully realize their potential in API production.

## Abstract 2

This study compares batch and continuous-flow manufacturing for seven pharmaceuticals, quantifying how flow chemistry enhances sustainability and reduces costs.

## 1 Introduction

Medicines are essential to human health and the well-being of a growing and aging global population, playing a key role in achieving the United Nations Sustainable Development Goal on “Good Health and Well-Being”. Pharmaceuticals encompass a wide variety of chemical compounds designed to ensure safe and effective therapies. Among these, active pharmaceutical ingredients (APIs) are specific chemical entities intended to exert an effect on chemical and biological targets. Currently, the manufacturing of APIs largely relies on traditional batch processes.^1−5^ One factor contributing to the extensive use of batch manufacturing is the adaptability of chemical production units, enabling the synthesis of a wide range of APIs within the same facilities.^6^ This flexibility allows manufacturers to switch between different production processes with relative ease, accommodating varying chemical reactions and adjusting to market demands without the need for extensive reconfiguration or new infrastructure. However, batch synthesis methods present limitations in process productivity, resulting in inefficient mixing of the reaction mixture, limited heat and mass transfer characteristics, and lower process safety margins.^7,8^ Additionally, scaling up batch processes poses significant challenges due to a lower surface area-to-volume ratio, which raises concerns about heat transfer control, hotspot formation, and insufficient mixing.^9,10^ As a result, the production of APIs is recognized as one of the most energy- and material-intensive industries within the chemical sector, contributing (only in 2023) to 358 megatons of greenhouse gas emissions.^11,12^ This is equivalent to 6% of global CO_2_ emissions, more than those from the automotive industry. This critical issue often flies under the radar, but is due to the industry’s stringent regulatory requirements, high-risk aversion, and the need for extensive validation and market acceptance.

**Figure 1:**
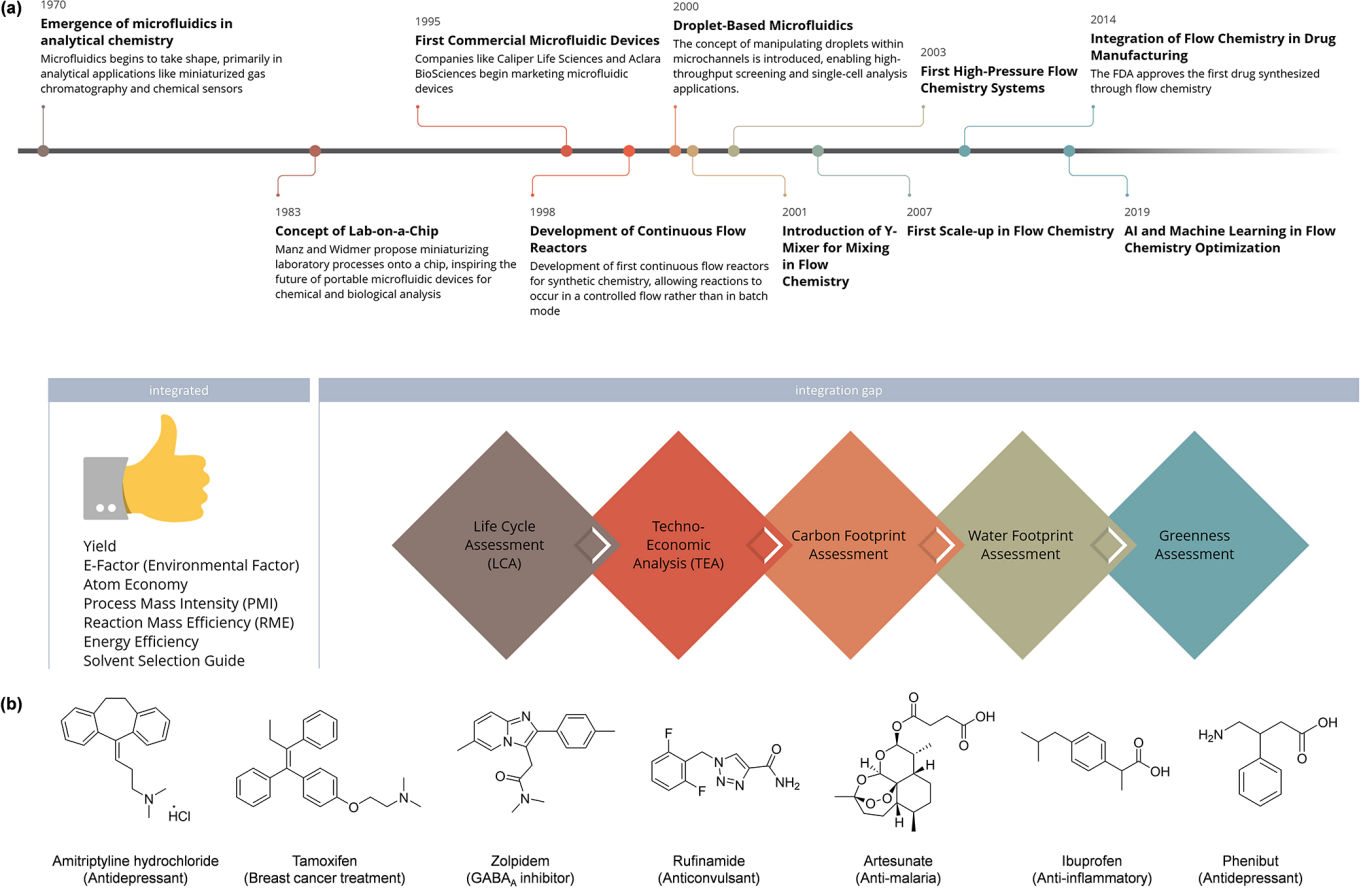
Timeline highlighting the key milestones in the conceptualization and development of flow chemistry and the stage of integration of green methods into flow chemistry practices (a). Chemical structures and applications of the seven APIs investigated in this work (b). The manufacturing methods, including specific operating conditions, are detailed in the Supporting Information.

Over the past two decades, continuous-flow processes have emerged as a potential alternative to batch processes in the preparation of APIs, and many drugs, including artemisinin and rufinamide, have been transitioned to continuous flow operation (Figure 1a).^13,14^ Flow techniques promise better control of operating conditions, leading to safer operations, homogenized mixing, reduced waste, enhanced transfer phenomena, and lower solvent and energy usage compared to batch reactors.^15−19^ It has been also hypothesized that the potential of this technology to enhance sustainability lies in its capacity to reduce the environmental footprint compared to conventional methods.^20^ However, to date, comparisons of API production processes in batch and continuous-flow configuration still primarily exploit basic green metrics such as the E-factor, process mass intensity (PMI), and yield analysis.^21^ While these metrics are valuable for assessing certain aspects of process sustainability, such as material efficiency and waste generation, they fall short of providing a complete evaluation of the overall environmental impact of a process and do not account for critical factors such as energy consumption, greenhouse gas emissions, and the broader life-cycle implications of raw material sourcing, waste disposal, and process scalability (Figure 1a). Additionally, these metrics often overlook economic feasibility, which is essential for integrating sustainable practices in real-world industrial applications, especially when considering capital and operational costs, regulatory compliance, and market acceptance,^22^ as also emphasized in a recent critical review.^22^ Therefore, it is not surprising that a comprehensive techno-economic analysis (TEA) and life-cycle assessment (LCA) encompassing the full scope of API production in flow, and the holistic evaluation of the batch-to-microreactor transition, has never been conducted. In particular, TEA is important for assessing a process’s economic feasibility considering capital investment and operating costs along with service life, maintenance requirements, and utilities.^23^ Additionally, LCA evaluates the environmental impacts of processes in a standardized manner.^24^ This highlights the need for a detailed investigation that thoroughly assesses the economic feasibility and environmental impact across a diverse range of APIs, to pave the way for more informed and sustainable manufacturing practices.

In this study, we present for the first time a complete TEA and LCA of manufacturing processes for a diverse set of seven different APIs, including the production of amitriptyline hydrochloride, tamoxifen, zolpidem, rufinamide, artesunate, ibuprofen, and phenibut (Figure 1b). By comparing manufacturing methods for seven drugs that have been industrially conducted in both batch and flow, we fill a gap in literature by contributing an integrated analysis of batch and continuous-flow techniques with techno-economic and life-cycle assessments.

## 2 Results and Discussion

The analysis focused on seven distinct APIs—amitriptyline hydrochloride,^25−28^ tamoxifen,^29,30^ zolpidem,^31−33^ rufinamide,^34−36^ artesunate,^37−39^ ibuprofen,^40−44^ and phenibut^45,46^—each selected for their industrial relevance and diverse chemical properties. These APIs span a range of therapeutic applications, including antidepressants (amitriptyline), anticancer agents (tamoxifen), sedatives (zolpidem), antiepileptics (rufinamide), antimalarials (artesunate), nonsteroidal anti-inflammatory drugs (ibuprofen), and neuroactive compounds (phenibut).^25−46^ The experimental conditions for both batch and continuous-flow processes were derived from a combination of patent data and peer-reviewed literature, ensuring that the selected parameters reflect realistic and scalable industrial practices. These conditions are depicted in Figures S1–S7, Supporting Information. As the reader can appreciate, by selecting APIs with diverse chemical structures and synthetic pathways, we aimed to ensure a broad evaluation of both economic feasibility and environmental impacts, making the findings more widely applicable and rigorous to the pharmaceutical sector. The process simulations and techno-economic analysis were performed using Aspen Plus V11 while the environmental assessment was achieved using SimaPro V9.5. Detailed analytical data are included in the Supporting Information.

### 2.1 Techno-Economic Analysis of the Seven Processes

**Figure 2:**
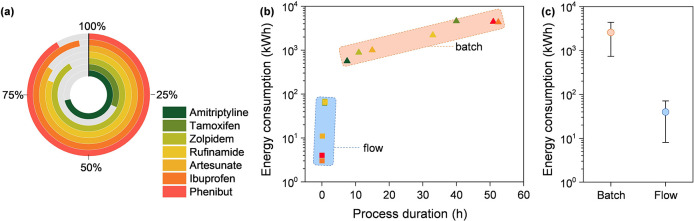
Energy reductions per gram of product and per hour in the batch-to-flow manufacturing transition (a). Correlation between total energy consumption and process duration for the seven processes in batch and flow (b). Statistical analysis of the batch and flow processes where the circle represents the average value, and the whisker shows ±1 standard deviation (c). Color codes in (a) apply to (b) and (c) as well.

We initiated the work by conducting a techno-economic analysis to evaluate the feasibility and cost-effectiveness of the flow technology, which is crucial for obtaining essential data on resource utilization. We quantified, in particular, the energy used in the two configurations (Table S1, Supporting Information). Generally, the batch manufacturing process exhibited energy consumption ranging from 1 × 10^–1^ W h^–1^ g_product_^–1^ to 1 × 10^2^ W h^–1^ g_product_^–1^. In contrast, the continuous-flow process demonstrated a significantly lower range from 10^–2^ W h^–1^ g_product_^–1^ to 10^1^ W h^–1^ g_product_^–1^. From a quantitative perspective, the implementation of the continuous-flow process enhanced energy efficiency by one order of magnitude compared to the batch process, consistently achieving an overall enhancement exceeding 30% and with an average improvement of approximately 78% (Table S1, Supporting Information and Figure 2a). The ibuprofen flow process demonstrated the highest enhancement by 97%. This exceptional performance was accompanied by 91% reduction in energy consumption for the phenibut process, from 9.51 W g_product_^–1^ h^–1^ to 0.82 W g_product_^–1^ h^–1^. These results stemmed from the continuous-flow technique improving productivity, owing to higher yields and a greater amount of target products in a shorter time. The lowest fraction of minimization in energy efficiency was observed in the tamoxifen process, which decreased from 1.49 W g_product_^–1^ h^–1^ to 0.99 W g_product_^–1^ h^–1^. The increased energy efficiency observed in the continuous-flow system was attributed to several characteristics inherent to continuous-flow technology. We explored correlations with multiple factors, such as reaction kinetics and advanced mass and heat transfer, but we could find that energy efficiency primarily correlates with process duration alone. This suggests that shorter reaction times in the continuous-flow system, which inherently demands less electricity, drive the observed reduction in total energy consumption. This relationship is illustrated for the seven processes in Figure 2b.

**Figure 3:**
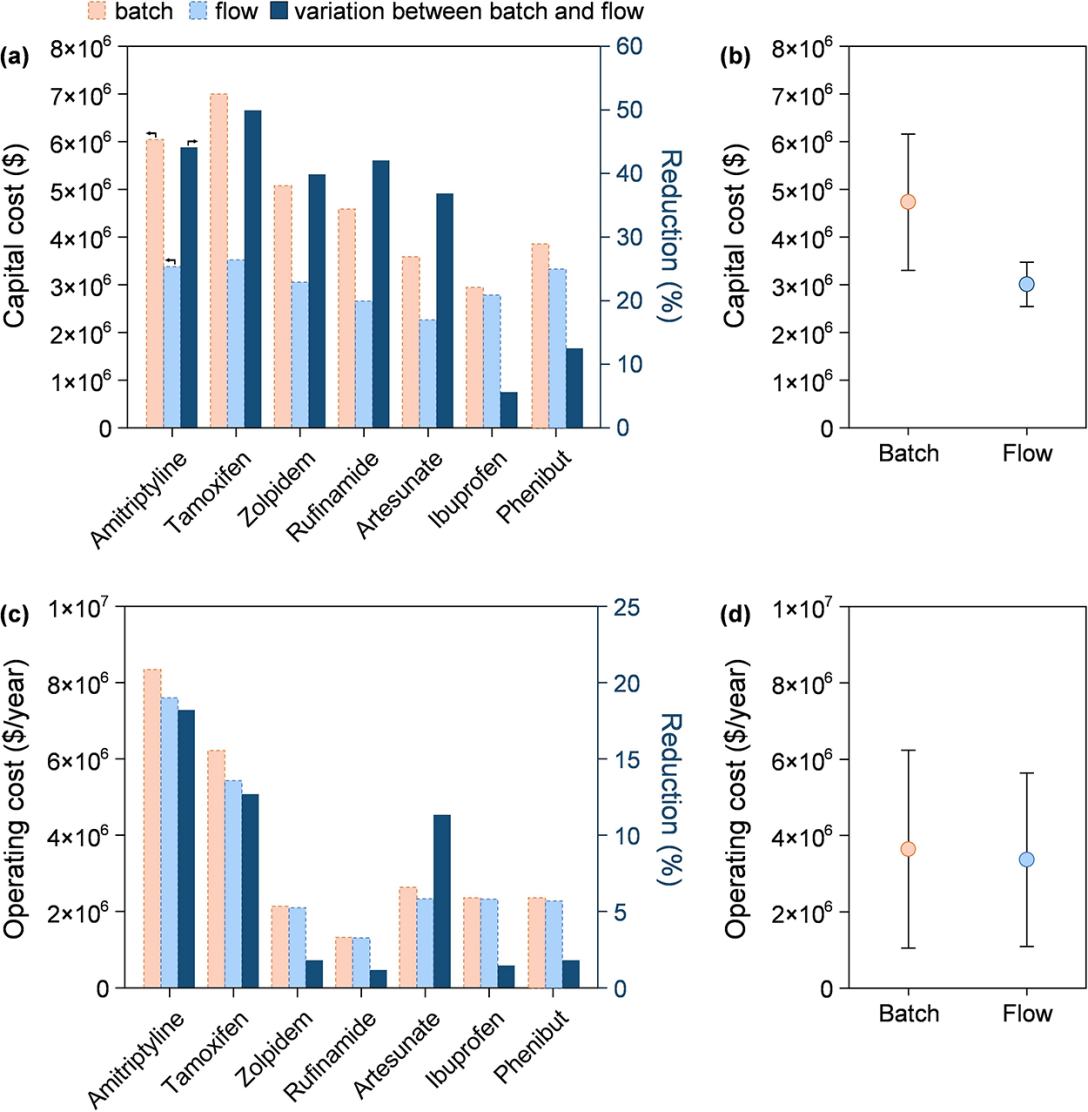
Capital cost expenses for the seven processes in batch and flow, and cost reduction in the batch-to-flow transition (a). Statistical analysis of capital cost results (b). Operating costs per year for the seven processes in batch and flow (c). Statistical analysis of the operating cost results (d). Color codes in (a) apply to all. The circle in the statistical analysis in (b) and (d) represents the average value, and the whisker shows ±1 standard deviation.

The statistical analysis of energy consumption for batch and flow processes, as shown in Figure 2c, demonstrated the performance difference in the energy efficiency between the two methods. Batch processes exhibit an average energy consumption of approximately 10^3^ kWh. Conversely, flow processes showed a statistically relevant reduced energy consumption on the order of 10^1^–10^2^ kWh. The capital cost expenses of the processes and their reductions are illustrated in Figure 3a (and detailed in Table S2, Supporting Information). The batch configuration was estimated to cost between $3,000,000 and $7,000,000, whereas the continuous-flow technology ranged from $2,000,000 to $4,000,000. From an economic point of view, the continuous-flow method leads to a less drastic decrease in capital cost expenses. The best performance in terms of capital cost reduction was observed in the rufinamide process, which experienced an almost 50% drop from $7,030,000 to $3,520,000. However, capital cost reductions are case-dependant, as it was also noted that other processes, such as ibuprofen, exhibited reduction performance below 10%. Capital costs between batch and flow processes for the seven APIs were statistically investigated in Figure 3b. The batch process demonstrates a slightly lower average operating cost compared to the flow configuration. The observed variability in the operating costs is primarily attributed to differences in infrastructure and instrumentation expenses, which account for nearly 50% of the total costs. The higher average and variability of batch processes indicated that the method requires more resource-intensive infrastructure (Table S3, Supporting Information). In fact, in continuous systems, the reactor operates with a consistent feed and flow of reactants, which allows for a compact design optimized for efficient mass and heat transfer. This reduction in reactor volume directly lowers the quantity of construction materials needed as well as associated costs. In addition to the capital cost expenses, yearly operating costs were also assessed, considering the raw material and the utility and maintenance expenses (Figure 3c). The batch manufacturing processes exhibited an average annual operating cost of $3,640,000 on average, while the flow configuration demonstrated a slight reduction in expenses, averaging $3,360,000 on average (Table S4, Supporting Information). However, for the seven processes, variations in operating costs were not statistically significant (Figure 3d). In other words, the reduction in costs observed with the flow configuration is likely not due to a definitive trend that can be generalized across chemical processes. The analysis, in fact, suggests that there is no strong evidence to support that the flow configuration consistently leads to lower operating costs in a meaningful way.

These results are particularly noteworthy because they are not solely dependent on a specific process but reflect, in statistical terms, the broader perspective on the advantages of continuous manufacturing. In transitioning from batch to continuous-flow methods, capital costs may be reduced due to smaller reactor sizes. This reduction is especially beneficial for the pharmaceutical sector, where high initial investments can hinder the adoption of new technologies. However, annual operating costs might not experience a similar decline. For pharmaceutical companies, this implies that although the initial capital investment in continuous-flow systems is appealing, the decision to transition should also take into account long-term operational efficiencies and the potential for improved product quality and consistency. Companies may find it most advantageous to adopt flow manufacturing, for example, for high-volume production of well-established products, where capital savings can be complemented by operational efficiencies over time or for new product developments when launching new pharmaceutical products. This approach allows companies to design processes specifically optimized for continuous flow, sidestepping the complexities and costs associated with retrofitting existing batch systems and facilitating a smoother integration of advanced technologies from the outset.

In addition, a study of 15-year net present value (NPV) projections at a discount rate of 8% (Figure S8, Supporting Information) was conducted to demonstrate clear financial advantages of flow processes over batch processes across all seven APIs. Initially, both methods show negative NPV values due to the high capital investment required. However, flow processes consistently achieve breakeven points earlier, with breakeven times ranging from year 7 to year 12 depending on the API, compared to later breakeven points for batch processes, which occur between years 9 and year 14. This earlier breakeven for flow processes is attributed to their reduced operating costs, lower resource consumption, and streamlined operations. Additionally, the slope of the NPV analysis for flow processes is steeper than that for batch processes, indicating higher profitability growth over time. By the end of the 15-year period, flow processes show consistently higher NPV values across all APIs, reflecting their superior long-term economic viability. The widening gap between the NPV curves of batch and flow methods highlights the cumulative financial advantage of flow processes, which stems from their efficiency in energy, solvent, and water usage, as well as lower environmental impacts, as demonstrated in previous analyses.

### 2.2 Life-Cycle Analysis of the Seven Processes

In order to evaluate the environmental impact and sustainability of the manufacturing processes, we then moved to a comprehensive LCA of the seven selected processes to identify key areas where improvements can be made, quantify resource consumption, and evaluate emissions associated with each process.

**Figure 4:**
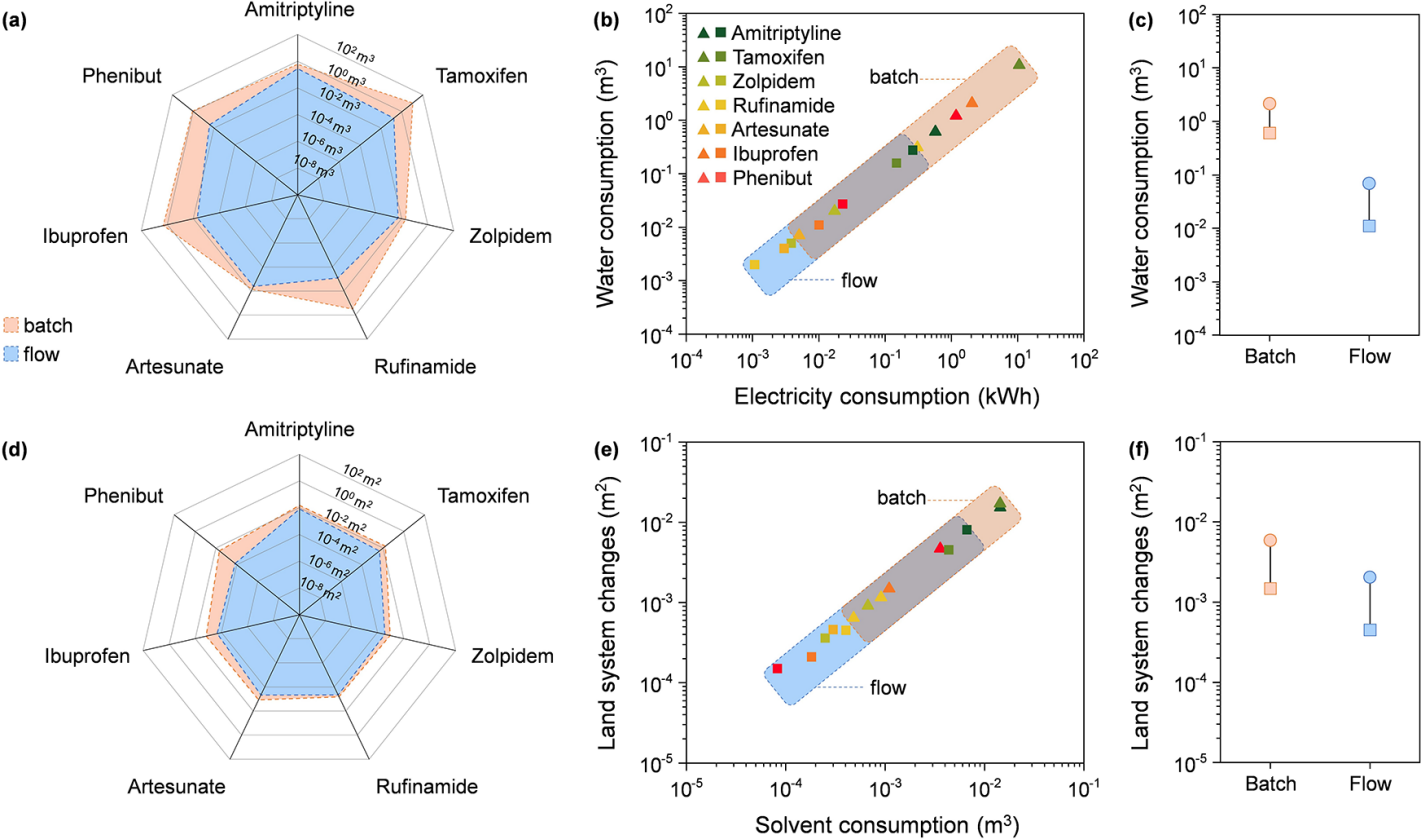
Water consumption for the seven processes in batch and flow (a). Correlation between water consumption and electricity usage (b). Statistical analysis of the water consumption results (c). Land system change for the seven processes in batch and flow (d). Correlation between land system changes and solvent consumption (e). Statistical analysis of the land system changes results (f). Color codes in (a) apply to all. The circle in the statistical analysis in (c) and (f) represents the average value, the square represents the median, and the line serves as a guide for the reader. Data are per gram of target product.

Regarding water consumption (Figure 4a), the batch manufacturing process resulted in a usage ranging between 10^–2^ and 10^1^ m^3^ per unit of product, whereas the continuous-flow process demonstrated a significantly lower range, between 10^–3^ and 10^–1^ m^3^ per unit of product (detailed in Table S5, Supporting Information). This indicates that the continuous-flow process consumes one-to-two orders of magnitude less water compared to the batch process, leading to a reduction of water usage of between 50 and 90%. The reduction in water consumption in the continuous-flow process can be attributed to several factors inherent to this technology. Continuous-flow systems generally operate at lower temperatures and pressures, resulting in the more efficient utilization of reagents and solvents. This efficiency leads to decreased water usage needed for cooling and cleaning unit operations. Additionally, the compact design and enhanced control over reaction parameters in continuous-flow systems further reduce the need for solvents and water, as reactions occur in a more controlled and steady-state environment, minimizing the requirements for excess water in the handling and processing stages. The greatest reduction was obtained in the ibuprofen production process, which achieved a 99% reduction in total solvent usage per gram of the target product, from 20.70 m^3^ throughout the batch process to 5.4 m^3^ in the continuous-flow method. In contrast, the artesunate production process exhibited the lowest reduction in total water consumption, with a decrease of only 46%, from 0.0072 m^3^ to 0.0039 m^3^, well below the average reduction of 81% in most of the seven pharmaceutical processes.

A correlation between water consumption and electricity usage is visible (Figure 4b). Specifically, lower water usage can be linked with reduced electricity consumption as less energy is required for cooling and processing operations. Thus, this linear relationship points out the significance of water efficiency not only in terms of resource conservation but also in terms of its potential impact on overall energy consumption and environmental sustainability. In fact, the water-energy nexus is a critical consideration in evaluating manufacturing processes due to the interdependence between water consumption and electricity usage. The statistical analysis supported the variations in water consumption between batch and flow processes (Figure 4c).

The effects on land system changes, which consider the impact of technology on deforestation and destruction of natural habitats, are depicted in Figure 4d. It was observed that the batch processes exhibited a change on land systems ranging from 10^–4^ to 10^–2^ m^2^ per unit of product, while the continuous-flow method led to a slight reduction of one order of magnitude, resulting in a range of 10^–4^ to 10^–3^ m^2^ per unit of product (detailed in Table S6, Supporting Information). Considering 1 gram of product, the phenibut process showed the best performance with a decrease of 97% from 0.00468 m^2^ in batch to 0.00015 m^2^ in flow, due to the minimization of toluene and tetrahydrofuran usage from 23.3 g g_product_^–1^ and 17.1 g g_product_^–1^ in batch to 7.6 g g_product_^–1^ and 0.1 g g_product_^–1^ in flow, respectively. In addition, ibuprofen exhibited a reduction of more than 85% in land system changes because of less solvent (methanol) usages (from 48.2 g g_product_^–1^ in batch to 3.5 g g_product_^–1^ in flow). Moreover, the rufinamide process demonstrated only a 30% reduction due to the similar amounts of dimethyl sulfoxide (DMSO) used during the manufacturing processes of the two configurations, noted as 14.0 g_DMSO_ g_product_^–1^ and 11.9 g_DMSO_ g_product_^–1^, respectively. The correlation between land system changes and solvent usage in Figure 4e can be attributed to the environmental footprint associated with the production and disposal of solvents. The extraction of raw materials for solvent production often involves land use changes, including deforestation and habitat destruction. Thus, reducing solvent consumption not only lessens the direct environmental impact of manufacturing processes but also diminishes the associated land use changes linked to solvent life-cycle management. In the cases of phenibut and ibuprofen, the significant reductions in solvent usage led to substantial decreases in land system changes, illustrating that efficient resource management in manufacturing can contribute to minimizing ecological disruption. Conversely, in the rufinamide process, the limited reduction in solvent usage resulted in only a modest decrease in land system change, highlighting the importance of optimizing solvent utilization as a strategy for promoting environmental sustainability within pharmaceutical manufacturing. The statistical analysis presented in Figure 4f supports these findings, although it also shows that the variation of land system changes the variation in land system changes is not statistically significant, and this might indicate that solvent consumption is not substantially different between batch and flow configurations. This could mean that while flow processes are often expected to reduce solvent usage due to continuous operation and improved efficiency, in practice, the reduction is not as pronounced. This could be due to the need for comparable solvent volumes for reaction control, cleaning, or separation steps, making the overall solvent consumption between the two processes relatively similar.

**Figure 5:**
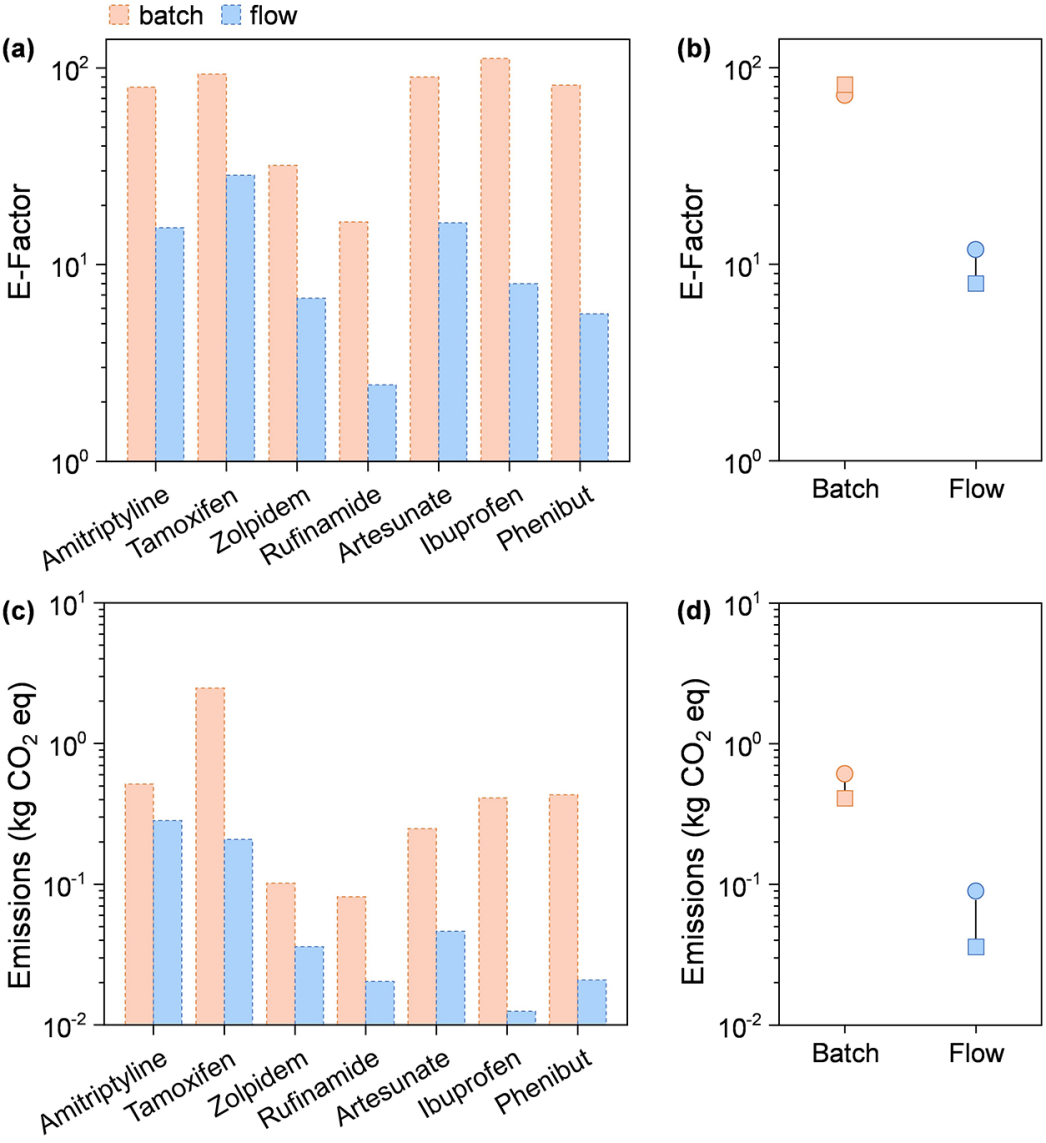
E-factor for the seven processes in batch and flow (a). Statistical analysis of the E-factor results (b). CO_2_ emissions for the seven processes in batch and flow (c). Statistical analysis of the carbon emission results (d). Color codes in (a) apply to all. The circle in the statistical analysis in (b) and (d) represents the average value, the square represents the median, and the line serves as a guide for the reader.

The environmental performance of the seven processes was investigated by evaluating their greenness, considering both the E-factor (mass of waste per mass of product) and carbon emissions (kg CO_2_ equiv). Continuous-flow processes generated fewer processes relative to the target product (Figure 5a). In fact, the E-factor of batch processes ranged between 10 and 110, while continuous-flow technology significantly outperformed batch methods, exhibiting an E-factor range of 2 to 20, hence with an average reduction of 87% (detailed in Table S7, Supporting Information). This improvement can be attributed to the intrinsic characteristics of continuous-flow techniques, including overall higher yields, lower waste production, and minimization of solvent usage, as discussed above. Notably, with respect to the shift from traditional batch reactors to a continuous-flow method, the artesunate process showed an excellent example of green production, achieving a 97% drop in E-factor. In addition, the phenibut and ibuprofen processes also followed this trend with ca. 93% reductions, respectively. Also, the rufinamide process performed the lowest drop in E-factor by 85%. The higher E-factor in batch processes is demonstrated in Figure 5b, which well depicts the high propensity for waste generation in batch systems.

It is important to note, however, that while the E-factor provides valuable insights into waste generation, it does not account for the carbon emissions associated with the production processes. This limitation means that the E-factor alone may not fully capture the environmental impact of a manufacturing process, particularly in terms of its contribution to greenhouse gas emissions. Carbon emissions can arise from various sources, including energy-intensive thermal processes required for heating, cooling, or phase changes, as well as from solvent evaporation, reagent decomposition, and byproduct formation. Additionally, emissions may result from upstream activities such as raw material extraction, transportation, and purification, as well as downstream processing steps like separation, purification, and waste treatment. Therefore, an assessment of carbon (CO_2_) emissions was also performed for the batch and flow methods (Figure 5c,d).

Batch configurations resulted in carbon emissions (kg of CO_2_ equiv) ranging from the order of magnitude of 10^–1^ to 10^1^, and via the implementation of the continuous-flow method, the emissions were obtained in a range of 10^–2^ to 10^–1^. The flow technology significantly lowered the carbon emissions by one order of magnitude with a 79% reduction on average (Table S8, Supporting Information). Similar to the results of E-factor, the ibuprofen process demonstrated an outstanding reduction performance by 97% from emitting 0.41 kg of CO_2_ equiv to 0.01 kg of CO_2_ equiv. This outcome was obtained during the analysis due to longer durations (to produce the same amount of product) for batch reactors, which led to higher electricity usage throughout the manufacturing stage. Additionally, the phenibut process also followed this well-executed trend by performing a 95% decrease from 0.43 kg of CO_2_ equiv to 0.02 kg of CO_2_ equiv. Finally, carbon emissions in the amitriptyline hydrochloride process showed the lowest minimization by 45% as a result of the implementation of the continuous-flow method.

**Figure 6:**
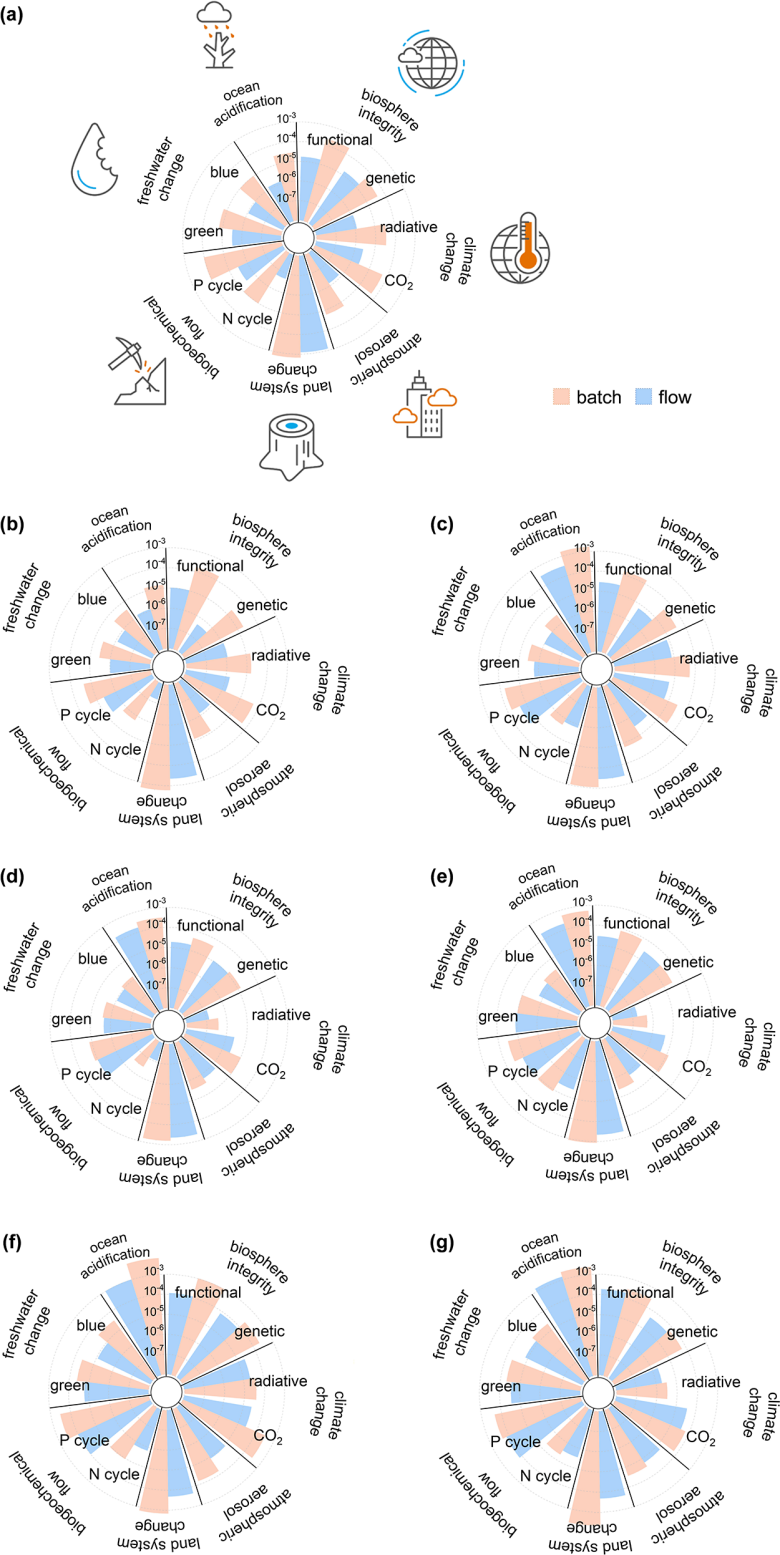
Integrated planetary analysis for the seven processes in batch and flow. The processes analyzed are amitriptyline (a), tamoxifen (b), zolpidem (c), rufinamide (d), artesunate (e), ibuprofen (f), and phenibut (g). Color codes in (a) apply to all. All numerical raw data used to construct the plots for each individual process are provided in Tables S16–S75, Supporting Information.

These findings highlight important insight regarding the interplay between CO_2_ emissions, water consumption, and energy use. Notably, some processes that exhibit low E-factors, such as rufinamide, may still generate significant CO_2_ emissions and consume considerable amounts of water due to their high energy requirements. Therefore, an integrated approach to sustainability assessment is essential to make informed decisions that promote sustainability across all dimensions of production. Finally, the environmental impacts of the seven processes were analyzed for the first time in the flow chemistry field in relation to the nine planetary boundaries (detailed in Tables S9–15, Supporting Information), including ocean acidification, biosphere integrity (both functional and genetic), carbon emissions, atmospheric aerosol loading, land system change, biogeochemical flows (in terms of phosphorus and nitrogen cycles), and freshwater use (Figure 6). The average reduction in ocean acidification across the seven processes was 72%. The phenibut process demonstrated the greatest reduction, with a 95% decrease, due to the lower electricity consumption and operating hours of the continuous-flow method compared to batch processing as well as reduced benzaldehyde feedstock usage. The ibuprofen process similarly showed a 92% reduction in ocean acidification. In contrast, the amitriptyline hydrochloride process exhibited the smallest reduction, at 41%, primarily due to the reduction in tetrahydrofuran (THF) usage. Atmospheric aerosol loading, a critical environmental category linked to human health due to fine particulate matter affecting air quality, was significantly reduced in the phenibut and ibuprofen processes with reductions of 95 and 90%, respectively. These reductions were largely driven by the decreased use of trimethyl orthoformate (TMOF) and THF. Furthermore, continuous-flow methods led to a greater than 60% reduction in the phosphorus cycle impacts for five of the seven processes. In the case of the nitrogen cycle, the phenibut process exhibited an impressive 98% reduction, largely due to decreased nitromethane and toluene usage, highlighting the efficiency of the continuous-flow technique and its lower solvent requirements. The impacts on genetic biosphere integrity, which are related to the carcinogenic effects of chemicals on the ecosystem, were also investigated. The ibuprofen process achieved a 98% reduction in this category, primarily due to a substantial decrease in the use of isobutylbenzene (1.12 to 0.21 g) per gram of product.

The Midpoint method performed a detailed evaluation of environmental impacts across the aforementioned 18 categories and enabled an in-depth examination of component-specific contributions and facilitated the identification of critical environmental hotspots. To build upon these outcomes and to offer an alternative perspective, both batch and flow techniques for the seven different API manufacturing processes were assessed via an Endpoint method. The Endpoint method consolidated the Midpoint impacts into three higher-level damage categories such as human health, ecosystem quality, and resource scarcity. By combining the precise Midpoint results with the aggregated Endpoint outcomes, we provide a robust and comprehensive assessment of the environmental performance of the seven processes capturing both detailed category-specific impacts and their broader environmental effects. The implementation of the continuous-flow method resulted in an average reduction of 74% across the seven processes based on the average outcomes observed in the respective categories of the Endpoint method (Tables S22–23, S30–31, S38–39, S46–47, S54–55, S62–63, S72–75, Supporting Information). In addition, among the seven processes evaluated, the ibuprofen, phenibut, and tamoxifen processes demonstrated the most substantial reductions in damage categories, achieving average decreases of 95, 93, and 89%, respectively. In contrast, the amitriptyline process exhibited the lowest performance, with an average reduction of 43%. Based on the in-depth evaluation of the Endpoint method, it was observed that the Endpoint results demonstrated a consistent alignment with the trends observed in the Midpoint method analysis. Overall, this study highlighted once more the effectiveness of continuous-flow methods in enhancing the environmental performance across multiple dimensions.

## 3 Conclusions

In conclusion, we carried out a thorough sustainability and circularity assessment of seven manufacturing methods for well-known APIs, comparing continuous-flow techniques with batch processes. Our analysis quantified the impact of flow chemistry, revealing that the continuous-flow method significantly outperformed batch methods in terms of economic feasibility and environmental impact. Specifically:

- continuous-flow processes showed energy efficiency improvements by one order of magnitude, averaging a 78% reduction in energy consumption compared to batch processes.
- capital costs for batch processes ranged from $3 million to $7 million, while continuous-flow technology ranged from $2 million to $4 million, leading to a potential 50% cost reduction.
- continuous-flow processes utilized significantly less water (50–90% reduction) and led to lower CO_2_ emissions compared to batch processes (ca. 79%).
- continuous-flow processes had an average E-factor reduction of 87% (from 10–110 for batch processes, to 2–20 for continuous-flow processes).

The environmental effects were also studied through nine planetary boundaries. The implementation of the continuous-flow method resulted in reductions in carbon emissions, ocean acidification, water consumption, and atmospheric aerosol loading. Nevertheless, the variations in land system changes and operating costs were less relevant, and this highlights the need to focus also on optimizing solvent management and operating conditions to fully exploit the potential of the flow microreactor technology. Currently, these aspects are not being maximized, leading to inefficiencies and missed opportunities for improving sustainability and cost-effectiveness. Overall, our results can empower the pharmaceutical industry to make informed decisions regarding the adoption of continuous methods and could influence policy development by emphasizing the economic and environmental benefits of adopting more sustainable manufacturing practices in the industry.

## 4 Methods

### 4.1 Techno-Economic Analysis

Techno-economic analyses were performed using Aspen Plus V11 software. The simulations employed a stoichiometric reactor model, utilizing fractional conversions to replicate the precise experimental performance of the processes under steady-state conditions. The convergence tolerance was set to 0.0001. Physical and chemical characteristics of the components investigated were obtained from databases (i.e., APV110, APESV110, and NISTV110) integrated within the software. Operating conditions for the simulations (i.e., temperature, pressure, and molar flow rates) were directly extracted from the experimental reaction conditions. Electricity was also included as a utility. Equipment costs were calculated based on the actual reactor volumes, utilizing the Aspen Process Economic Analyzer (APEA) platform. To assess the raw material expenses, the cost of each chemical component was obtained from Merck. Profitability analyses (net present values) were conducted with an 8% discount rate. Sensitivity analysis of the TEA simulations is presented in Figure S9.

### 4.2 Life-Cycle Analysis

LCA studies were conducted using SimaPro V9.5 within the framework of a cutoff system model and cradle-to-gate analysis.^47^ The ReCiPe 2016 Midpoint (H),^48^ ReCiPe 2016 Endpoint (H),^48^ and Environmental Footprint 3.1 (EF)^49^ methods were employed to evaluate planetary boundaries. Specifically, ReCiPe 2016 Midpoint (H) methodology evaluated (i) climate change through global warming and ionizing radiation categories, (ii) atmospheric aerosol loading through the fine particulate matter category, (iii) biogeochemical flows through freshwater and marine eutrophication categories, (iv) land system change through the land use category, (v) freshwater change through freshwater and marine ecotoxicity categories, and (vi) functional and genetic biosphere integrities through their carcinogenic toxicity. Mineral and fossil resource scarcities, terrestrial acidification, and ozone formation were addressed using both the ReCiPe Midpoint and the EF methodologies. Ecoinvent-3 data sets were sourced from the life-cycle inventory database and used for the mass and energy flow data. The related energy usages for all processes were acquired through Aspen Plus V11 simulations. The sensitivity analysis of the LCA simulations is presented in Figure S9.

## Data Availability

All the data supporting the findings of this study are available within the article and its Supporting Information and from the corresponding authors upon reasonable request.

## Supporting Information Available

Additional experimental details, including model equations utilized in Aspen Plus V11 simulations, process layouts for batch and flow chemistry, combined techno-economic and sustainability analyses, and individual results for seven pharmaceutical processes (amitriptyline hydrochloride, tamoxifen, zolpidem, rufinamide, artesunate, ibuprofen, and phenibut). The document also includes profitability analysis; sensitivity analysis; energy efficiency data, capital and operational cost comparisons; environmental impact assessments (CO_2_ emissions, water consumption, land use, and waste generation), and planetary analyses for each process (PDF)
