# Supplementary material for: Sustainability and Techno-Economic Assessment of Batch and Flow Chemistry in Seven Industrial Pharmaceutical Processes
Source: ACS Sustain Chem Eng. 2025 Feb 14;13(7):2864–74. doi: 10.1021/acssuschemeng.4c09289 (PMC11864096; doi:10.1021/acssuschemeng.4c09289)
Supplement: Supplementary file 1 — sc4c09289_si_001.pdf [file sc4c09289_si_001.pdf]

Supporting Information

Sustainability and Techno-Economic Assessment of Batch and Flow Chemistry in Seven Industrial Pharmaceutical Processes

Mert Can Ince,[a] Brahim Benyahia,[b] and Gianvito Vilé,[a]*

[a] *Department of Chemistry, Materials, and Chemical Engineering "Giulio Natta", Politecnico di Milano, Piazza Leonardo da Vinci 32, 20133 Milano, Italy.*

[b] *Chemical Engineering Department, Loughborough University, Epinal Way, Loughborough, Leicestershire LE11 3TU, U.K.*

* *Corresponding author. E-mail: gianvito.vile@polimi.it (GV)*

Table of contents

1. Model equivuations utilized in the Aspen Plus V11 simulations[1] ....page S3
2. Process layouts..page S4
3. Combined analyses ....page S8
4. Amitriptylne hydrochloride results...page S23
5. Tamoxifen results ....page S30
6. Zolpidem results ....page S37
7. Rufinamide results ....page S43
8. Artesunate results..page S50
9. Ibuprofen results ....page S57
10. Phenibut results...page S66
11. References for Supporting Information ....page S75

1. Model equivuations utilized in the Aspen Plus V11 simulations[1]

The UNIQUAC (Universal Quasichemical) thermodynamic model was integrated into simulations, functioning as an advanced activity coefficient method employed in the comprehensive characterization of phase equilibria. The UNIQUAC model was well-suited in the accurate illustration of strongly non-ideal liquid solutions and in the detailed analysis of liquid-liquid equilibria. The model requires the specification of binary interaction parameters. Aspen includes numerous binary parameters in the vapor-liquid equilibrium (VLE) and liquid-liquid equilibrium (LLE) derived from both literature and the regression of experimental data in Aspen Physical Property System databanks. In addition, the Redlich–Kwong equation of state is an empirical and algebraic expression that defines the relationship between temperature, pressure, and volume in gases and it was employed as the basis in the thermodynamic model in the simulation system. Moreover, extended Antoine equation was used for determining the vapor pressure of the components. Heat of vaporization was calculated *via* Clausius-Clapeyron equation. Furthermore, heat capacities and liquid viscosities of the compounds were computed by using DIPPR methods. The Henry's constant model is applied in the context of Henry's Law to determine K-values in the gas components dissolved in a mixture. Also, Sato-Riedel thermal conductivity model was utilized to calculate the thermal conductivity of the gas phase components. Lastly, the Wilke-Chang equation was employed to estimate the diffusivity of the synthesized solid APIs in liquid solvents.

2. Process layouts

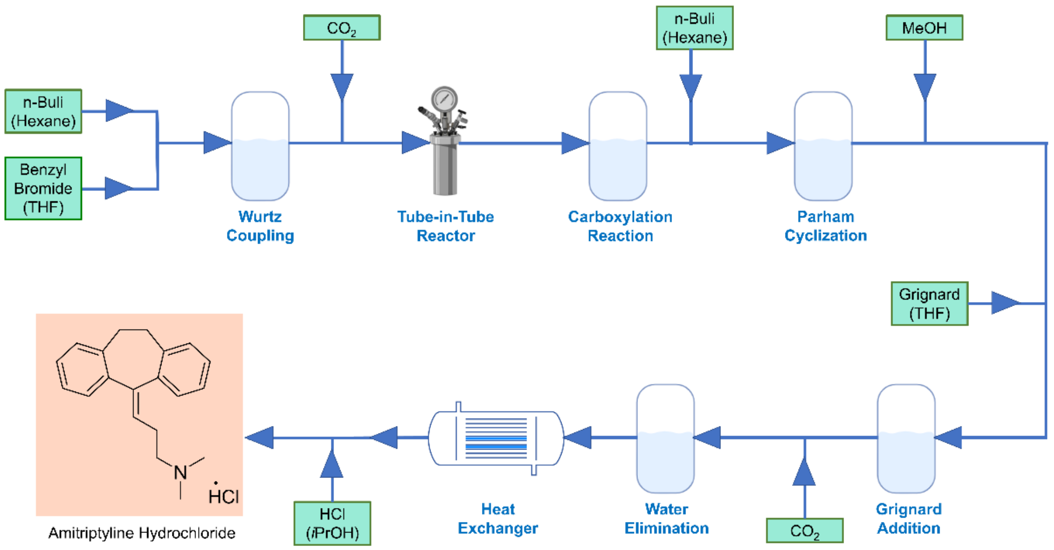

Figure S1. Illustration of amitriptyline hydchloride manufacturing stages.

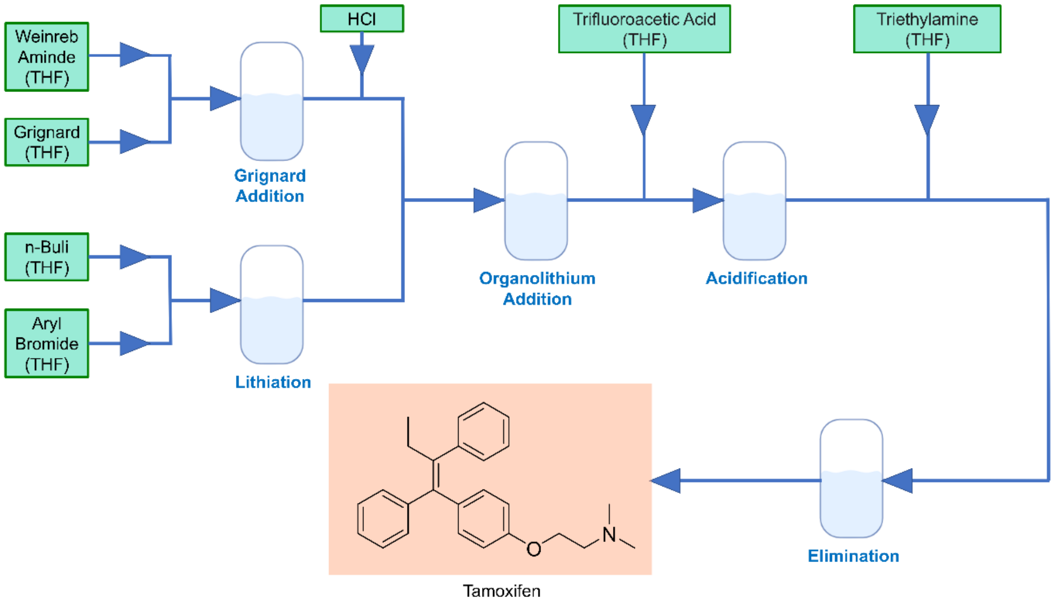

Figure S2. Illustration of tamoxifen manufacturing stages.

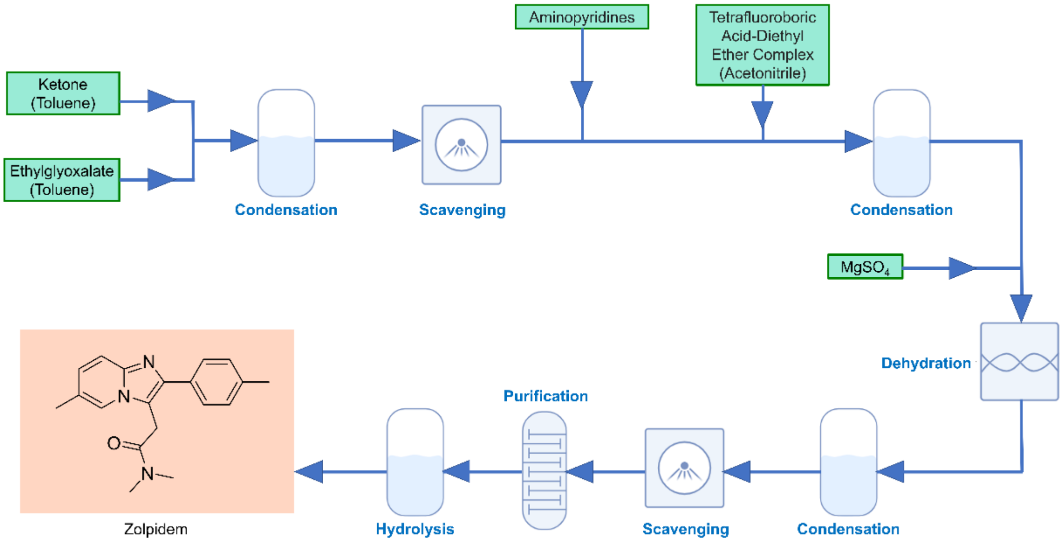

Figure S3. Illustration of zolpidem manufacturing stages.

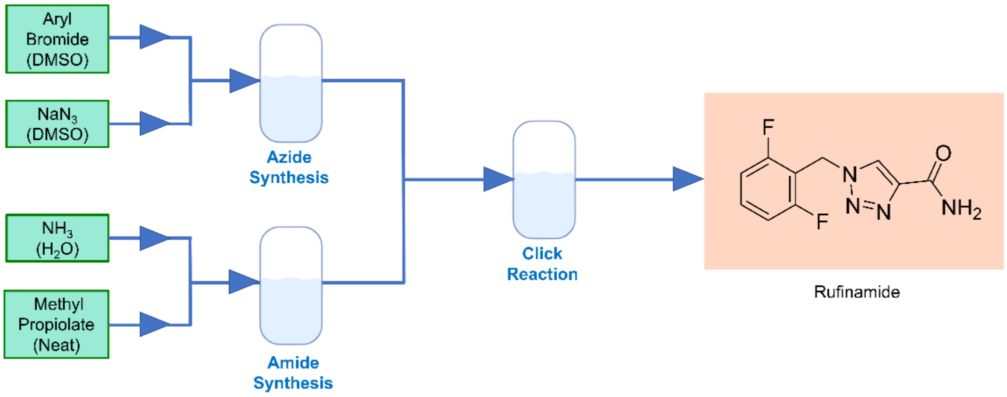

Figure S4. Illustration of rufinamide manufacturing stages.

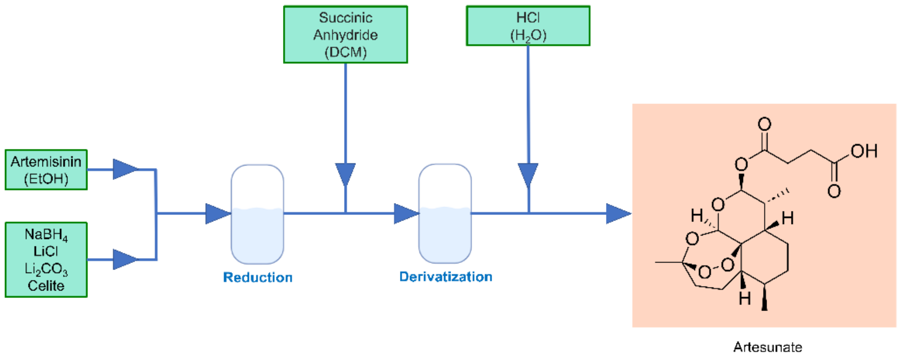

Figure S5. Illustration of artesunate manufacturing stages.

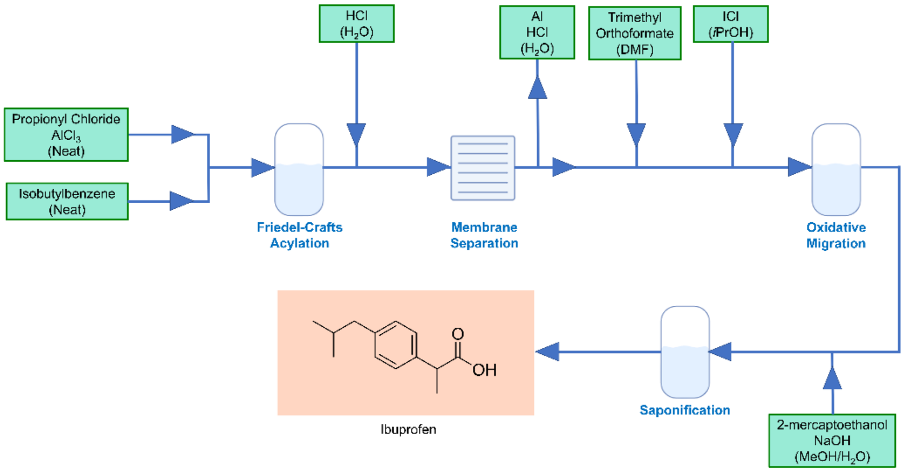

Figure S6. Illustration of ibuprofen manufacturing stages.

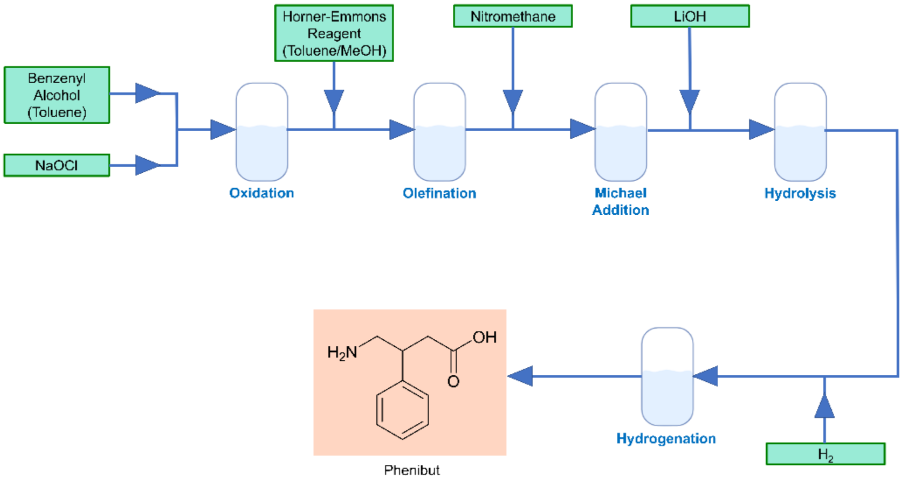

Figure S7. Illustration of phenibut manufacturing stages.

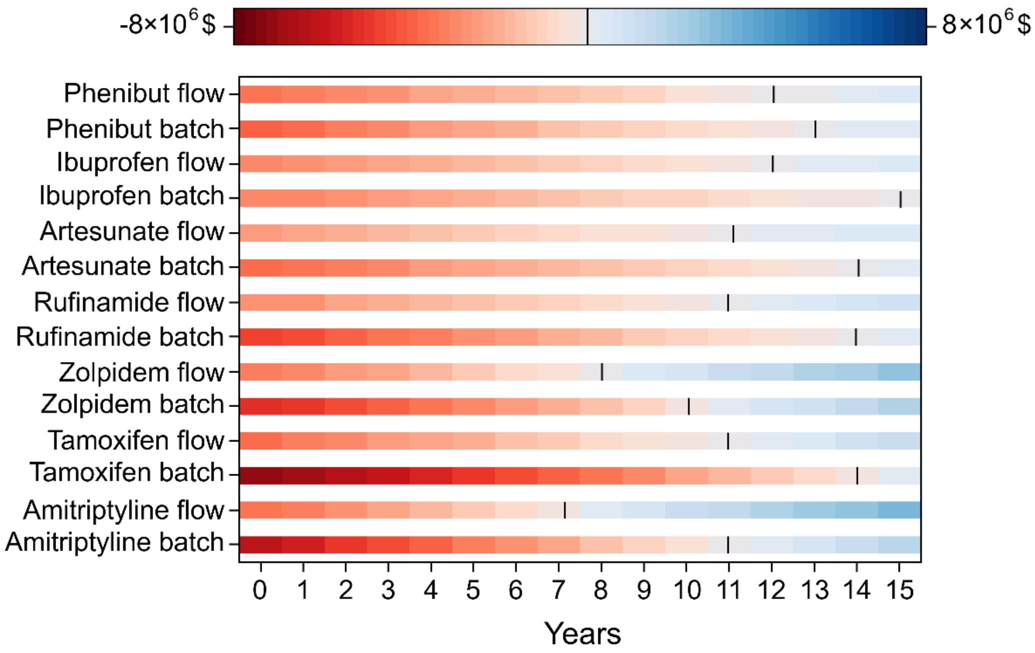

Figure S8. Profitability analysis (net present value) results for the seven differerent API processes comparing batch and continuous-flow methods .

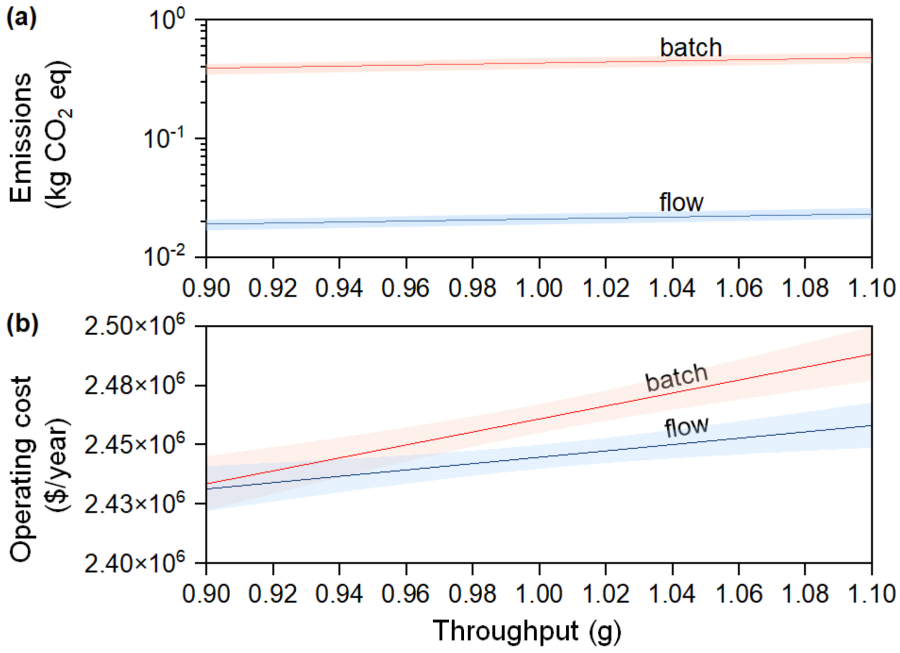

Figure S9. Sensitivity analysis results for the techno-economic and life-cycle analyses simulations.

3. Combined analyses

Table S1. Energy efficiency results in the seven different API processes, using traditional batch and continuous-flow methods.

Process	Method	Energy used ($W\ h^{-1}\ g_{product}^{-1}$)	Absolute reduction ($W\ h^{-1}\ g_{product}^{-1}$)	Reduction (%)
Amitriptyline hydrochloride	Batch	0.88	0.63	71.3
	Flow	0.25		
Tamoxifen	Batch	1.45	0.46	31.7
	Flow	0.99		
Zolpidem	Batch	3.47	3.17	91.3
	Flow	0.30		
Rufinamide	Batch	38.51	30.99	80.5
	Flow	7.52		
Artesunate	Batch	24.03	20.33	84.6
	Flow	3.70		
Ibuprofen	Batch	0.18	0.17	97.3
	Flow	0.01		
Phenibut	Batch	9.51	8.69	91.4
	Flow	0.82		

Table S2. Capital cost expenses in the seven different API manufacturing processes contrasting traditional batch and continuous-flow methods.

Process	Method	Capital cost ($\times 10^6$ \$)	Absolute reduction ($\times 10^6$ \$)	Reduction (%)
Amitriptyline hydrochloride	Batch	6.04	2.66	44.1
	Flow	3.38		
Tamoxifen	Batch	7.03	3.51	49.9
	Flow	3.52		
Zolpidem	Batch	5.08	2.03	39.9
	Flow	3.05		
Rufinamide	Batch	4.59	1.93	42.1
	Flow	2.66		
Artesunate	Batch	3.59	1.33	36.9
	Flow	2.26		
Ibuprofen	Batch	2.95	0.17	5.6
	Flow	2.78		
Phenibut	Batch	3.88	0.48	12.5
	Flow	3.40		

Table S3. Impact of the infrastructure and instrumentation costs on the capital cost expenses in batch and continuous-flow manufacturing methods.

Expenses	Amitriptyline hydrochloride		Tamoxifen		Zolpidem		Rufinamide		Artesunate		Ibuprofen		Phenibut	
	**batch**	**flow**	**batch**	**flow**	**batch**	**flow**	**batch**	**flow**	**batch**	**flow**	**batch**	**flow**	**batch**	**flow**
Infrastructure (10^5 \$)	7.8	3.4	9.3	3.0	6.1	2.3	5.6	1.8	3.7	1.2	1.5	1.4	2.3	1.9
Instrumentation (10^5 \$)	17	8.2	19	9.2	14	7.1	13	6.7	9.1	5.5	68	6.6	11	8.7
**Total capital cost (10^5 \$)**	**60**	**34**	**74**	**35**	**51**	**31**	**48**	**27**	**38**	**27**	**30**	**28**	**39**	**34**

Table S4. Operational cost expenses in the seven different API manufacturing processes contrasting traditional batch and continuous-flow methods.

**Process**	**Method**	**Operating cost ($\times 10^6$ $)**	**Absolute reduction ($\times 10^5$ $)**	**Reduction (%)**
Amitriptyline hydrochloride	Batch	8.34	7.36	18.2
	Flow	7.60		
Tamoxifen	Batch	6.22	7.89	12.7
	Flow	5.43		
Zolpidem	Batch	2.14	0.38	1.8
	Flow	2.10		
Rufinamide	Batch	1.32	0.14	1.2
	Flow	1.31		
Artesunate	Batch	2.63	2.98	11.3
	Flow	2.34		
Ibuprofen	Batch	2.36	0.34	1.5
	Flow	2.33		
Phenibut	Batch	2.46	0.22	0.9
	Flow	2.44		

Table S5. Water consumption results in the seven different API manufacturing processes contrasting traditional batch and continuous-flow methods.

Process	Method	Water consumption ($\times 10^{-1}$ m^3)	Absolute reduction ($\times 10^{-1}$ m^3)	Reduction (%)
Amitriptyline hydrochloride	Batch	6.02	3.28	54.5
	Flow	2.74		
Tamoxifen	Batch	107.00	105.42	98.5
	Flow	1.58		
Zolpidem	Batch	0.20	0.15	74.2
	Flow	0.05		
Rufinamide	Batch	3.09	3.08	99.3
	Flow	0.02		
Artesunate	Batch	0.07	0.03	45.7
	Flow	0.04		
Ibuprofen	Batch	20.70	20.65	99.5
	Flow	0.11		
Phenibut	Batch	12.00	11.73	97.7
	Flow	0.27		

Table S6. Land system changes results in the seven different API manufacturing processes contrasting traditional batch and continuous-flow methods.

Process	Method	Land system change ($\times 10^{-3}$ m^2)	Absolute reduction ($\times 10^{-3}$ m^2)	Reduction (%)
Amitriptyline hydrochloride	Batch	15.20	7.11	46.8
	Flow	8.09		
Tamoxifen	Batch	17.10	12.57	73.5
	Flow	4.53		
Zolpidem	Batch	0.91	0.55	60.7
	Flow	0.36		
Rufinamide	Batch	0.64	0.19	29.5
	Flow	0.45		
Artesunate	Batch	1.15	0.69	60.2
	Flow	0.46		
Ibuprofen	Batch	1.48	1.27	85.5
	Flow	0.21		
Phenibut	Batch	4.68	4.53	96.8
	Flow	0.15		

Table S7. E-factor results in the seven different API manufacturing processes contrasting traditional batch and continuous-flow methods.

Process	Method	E-factor (g_{waste} $g_{product}^{-1}$)	Absolute reduction (g_{waste} $g_{product}^{-1}$)	Reduction (%)
Amitriptyline hydrochloride	Batch	79.8	66.1	82.9
	Flow	13.7		
Tamoxifen	Batch	93.1	70.0	75.2
	Flow	23.1		
Zolpidem	Batch	31.9	25.2	78.9
	Flow	6.7		
Rufinamide	Batch	15.8	13.4	85.2
	Flow	2.4		
Artesunate	Batch	90.0	87.4	97.1
	Flow	2.6		
Ibuprofen	Batch	111.8	103.8	92.9
	Flow	8.0		
Phenibut	Batch	82.0	76.4	93.1
	Flow	5.6		

Table S8. CO_2 emissions results in the seven different API manufacturing processes contrasting traditional batch and continuous-flow methods.

Process	Method	CO_2 emission (kg CO_2 equiv)	Absolute reduction (kg CO_2 equiv)	Reduction (%)
Amitriptyline hydrochloride	Batch	0.51	0.23	44.7
	Flow	0.28		
Tamoxifen	Batch	2.48	2.27	91.6
	Flow	0.21		
Zolpidem	Batch	0.10	0.06	64.7
	Flow	0.04		
Rufinamide	Batch	0.08	0.06	75.0
	Flow	0.02		
Artesunate	Batch	0.25	0.20	81.4
	Flow	0.05		
Ibuprofen	Batch	0.41	0.40	97.0
	Flow	0.01		
Phenibut	Batch	0.43	0.41	95.2
	Flow	0.02		

Table S9. Results in the nine planetary boundaries analysis in the amitriptyline hydrochloride process. Abbreviated units of kBq Co-60 equiv, CFC11, 1,4-DCB and E/MSY correspond to kg Co-60 equiv, kg chlorofluorocarbon equiv, kg 1,4-dichlorobenzene (1,4-DB) equiv and extinctions per million species-years, respectively.

Category	Unit	Method	Results	Absolute reduction	Reduction (%)
CO_2 concentration	ton CO_2 equiv	Batch	5.10×10^{-4}	2.30×10^{-1}	43.6
		Flow	2.80×10^{-4}		
Radiative forcing	kBq Co-60 equiv	Batch	3.35×10^{-6}	1.40×10^{-6}	41.7
		Flow	1.95×10^{-6}		
S. ozone depletion	kg CFC11 equiv	Batch	6.74×10^{-5}	2.89×10^{-5}	43.0
		Flow	3.85×10^{-5}		
A. aerosol loading	kg PM2.5 equiv	Batch	2.84×10^{-5}	1.16×10^{-5}	41.0
		Flow	1.68×10^{-5}		
Ocean acidification	mol H^+ equiv	Batch	2.43×10^{-3}	1.01×10^{-3}	41.6
		Flow	1.42×10^{-3}		
P. cycle	kg P equiv	Batch	2.23×10^{-4}	8.36×10^{-5}	37.5
		Flow	1.39×10^{-4}		
N. cycle	kg N equiv	Batch	4.77×10^{-6}	2.01×10^{-6}	42.1
		Flow	2.76×10^{-6}		
Blue water	kg 1,4-DCB	Batch	2.32×10^{-5}	9.60×10^{-6}	41.4
		Flow	1.36×10^{-5}		
Green water	kg 1,4-DCB	Batch	4.89×10^{-5}	2.19×10^{-5}	44.8
		Flow	2.70×10^{-5}		
Land system change	m^2	Batch	1.50×10^{-2}	7.11×10^{-3}	45.9
		Flow	8.09×10^{-3}		
Functional B.I.	E/MSY	Batch	2.90×10^{-4}	1.06×10^{-4}	36.7
		Flow	1.84×10^{-4}		
Genetic B.I.	E/MSY	Batch	6.11×10^{-5}	1.61×10^{-5}	26.4
		Flow	4.50×10^{-5}		

Table S10. Results in the nine planetary boundaries analysis in the tamoxifen process.

Category	Unit	Method	Results	Absolute reduction	Reduction (%)
CO_2 concentration	ton CO_2 equiv	Batch	2.48×10^{-3}	2.27×10^{0}	91.6
		Flow	2.10×10^{-4}		
Radiative forcing	kBq Co-60 equiv	Batch	3.98×10^{-5}	1.81×10^{-5}	45.6
		Flow	2.16×10^{-5}		
S. ozone depletion	kg CFC11 equiv	Batch	1.43×10^{-4}	1.15×10^{-4}	80.0
		Flow	2.87×10^{-5}		
A. aerosol loading	kg PM2.5 equiv	Batch	6.60×10^{-5}	5.39×10^{-5}	81.7
		Flow	1.21×10^{-5}		
Ocean acidification	mol H^+ equiv	Batch	6.62×10^{-3}	5.60×10^{-3}	84.6
		Flow	1.02×10^{-3}		
P. cycle	kg P equiv	Batch	3.32×10^{-4}	2.34×10^{-4}	70.4
		Flow	9.83×10^{-5}		
N. cycle	kg N equiv	Batch	1.09×10^{-5}	9.37×10^{-6}	86.2
		Flow	1.50×10^{-6}		
Blue water	kg 1,4-DCB	Batch	3.53×10^{-5}	2.68×10^{-5}	75.9
		Flow	8.51×10^{-6}		
Green water	kg 1,4-DCB	Batch	4.10×10^{-5}	2.48×10^{-5}	60.5
		Flow	1.62×10^{-5}		
Land system change	m^2	Batch	1.71×10^{-2}	1.26×10^{-2}	73.4
		Flow	4.53×10^{-3}		
Functional B.I.	E/MSY	Batch	1.02×10^{-3}	9.06×10^{-4}	88.6
		Flow	1.17×10^{-4}		
Genetic B.I.	E/MSY	Batch	2.28×10^{-4}	1.53×10^{-4}	66.9
		Flow	7.55×10^{-5}		

Table S11. Results in the nine planetary boundaries analysis in the zolpidem process.

Category	Unit	Method	Results	Absolute reduction	Reduction (%)
CO_2 concentration	ton CO_2 equiv	Batch	1.00×10^{-4}	6.00×10^{-2}	64.5
		Flow	4.00×10^{-5}		
Radiative forcing	kBq Co-60 equiv	Batch	5.09×10^{-7}	3.36×10^{-7}	66.0
		Flow	1.73×10^{-7}		
S. ozone depletion	kg CFC11 equiv	Batch	2.66×10^{-6}	1.26×10^{-6}	47.5
		Flow	1.39×10^{-6}		
A. aerosol loading	kg PM2.5 equiv	Batch	4.07×10^{-6}	2.50×10^{-6}	61.5
		Flow	1.57×10^{-6}		
Ocean acidification	mol H^+ equiv	Batch	5.34×10^{-4}	3.29×10^{-4}	61.6
		Flow	2.05×10^{-4}		
P. cycle	kg P equiv	Batch	3.38×10^{-5}	2.13×10^{-5}	63.0
		Flow	1.25×10^{-5}		
N. cycle	kg N equiv	Batch	1.21×10^{-5}	8.34×10^{-6}	68.7
		Flow	3.79×10^{-6}		
Blue water	kg 1,4-DCB	Batch	2.68×10^{-6}	1.05×10^{-6}	39.0
		Flow	1.64×10^{-6}		
Green water	kg 1,4-DCB	Batch	1.01×10^{-5}	-1.71×10^{-6}	-16.9
		Flow	1.18×10^{-5}		
Land system change	m^2	Batch	9.10×10^{-4}	5.50×10^{-2}	60.7
		Flow	3.60×10^{-4}		
Functional B.I.	E/MSY	Batch	7.79×10^{-5}	4.96×10^{-5}	63.6
		Flow	2.84×10^{-5}		
Genetic B.I.	E/MSY	Batch	2.86×10^{-5}	8.11×10^{-7}	2.8
		Flow	2.78×10^{-5}		

Table S12. Results in the nine planetary boundaries analysis in the rufinamide process.

Category	Unit	Method	Results	Absolute reduction	Reduction (%)
CO_2 concentration	ton CO_2 equiv	Batch	8.00×10^{-5}	6.00×10^{-2}	75.0
		Flow	2.00×10^{-5}		
Radiative forcing	kBq Co-60 equiv	Batch	3.78×10^{-7}	2.41×10^{-7}	63.8
		Flow	1.37×10^{-7}		
S. ozone depletion	kg CFC11 equiv	Batch	4.91×10^{-6}	2.34×10^{-6}	47.7
		Flow	2.57×10^{-6}		
A. aerosol loading	kg PM2.5 equiv	Batch	2.96×10^{-6}	1.27×10^{-6}	42.8
		Flow	1.69×10^{-6}		
Ocean acidification	mol H^+ equiv	Batch	3.13×10^{-4}	1.45×10^{-4}	46.3
		Flow	1.68×10^{-4}		
P. cycle	kg P equiv	Batch	1.34×10^{-5}	3.43×10^{-6}	25.6
		Flow	9.98×10^{-6}		
N. cycle	kg N equiv	Batch	3.11×10^{-7}	2.28×10^{-7}	73.3
		Flow	8.32×10^{-8}		
Blue water	kg 1,4-DCB	Batch	1.40×10^{-6}	4.80×10^{-7}	34.3
		Flow	9.22×10^{-7}		
Green water	kg 1,4-DCB	Batch	2.53×10^{-6}	3.94×10^{-7}	15.5
		Flow	2.14×10^{-6}		
Land system change	m^2	Batch	6.40×10^{-4}	1.90×10^{-2}	29.6
		Flow	4.50×10^{-4}		
Functional B.I.	E/MSY	Batch	4.36×10^{-5}	2.46×10^{-5}	56.5
		Flow	1.90×10^{-5}		
Genetic B.I.	E/MSY	Batch	1.39×10^{-5}	3.04×10^{-6}	21.9
		Flow	1.09×10^{-5}		

Table S13. Results in the nine planetary boundaries analysis in the artesunate process.

Category	Unit	Method	Results	Absolute reduction	Reduction (%)
CO_2 concentration	ton CO_2 equiv	Batch	2.50×10^{-4}	2.00×10^{-1}	81.4
		Flow	5.00×10^{-5}		
Radiative forcing	kBq Co-60 equiv	Batch	5.38×10^{-5}	4.62×10^{-5}	85.9
		Flow	7.60×10^{-6}		
S. ozone depletion	kg CFC11 equiv	Batch	3.61×10^{-6}	1.96×10^{-6}	54.2
		Flow	1.65×10^{-6}		
A. aerosol loading	kg PM2.5 equiv	Batch	1.61×10^{-5}	1.29×10^{-5}	80.4
		Flow	3.15×10^{-6}		
Ocean acidification	mol H^+ equiv	Batch	1.70×10^{-3}	1.38×10^{-3}	81.0
		Flow	3.23×10^{-4}		
P. cycle	kg P equiv	Batch	6.01×10^{-5}	3.82×10^{-5}	63.7
		Flow	2.18×10^{-5}		
N. cycle	kg N equiv	Batch	2.19×10^{-6}	7.77×10^{-7}	35.5
		Flow	1.41×10^{-6}		
Blue water	kg 1,4-DCB	Batch	2.92×10^{-6}	1.90×10^{-6}	65.1
		Flow	1.02×10^{-6}		
Green water	kg 1,4-DCB	Batch	3.62×10^{-6}	1.96×10^{-6}	54.1
		Flow	1.66×10^{-6}		
Land system change	m^2	Batch	1.15×10^{-3}	6.90×10^{-4}	60.1
		Flow	4.60×10^{-4}		
Functional B.I.	E/MSY	Batch	1.54×10^{-4}	1.25×10^{-4}	81.5
		Flow	2.83×10^{-5}		
Genetic B.I.	E/MSY	Batch	3.91×10^{-5}	3.30×10^{-5}	84.3
		Flow	6.13×10^{-6}		

Table S14. Results in the nine planetary boundaries analysis in the ibuprofen process.

Category	Unit	Method	Results	Absolute reduction	Reduction (%)
CO_2 concentration	ton CO_2 equiv	Batch	4.10×10^{-4}	4.00×10^{-1}	97.0
		Flow	1.00×10^{-5}		
Radiative forcing	kBq Co-60 equiv	Batch	1.61×10^{-5}	1.49×10^{-5}	92.7
		Flow	1.18×10^{-6}		
S. ozone depletion	kg CFC11 equiv	Batch	1.70×10^{-6}	1.62×10^{-6}	94.9
		Flow	8.65×10^{-8}		
A. aerosol loading	kg PM2.5 equiv	Batch	8.04×10^{-6}	7.28×10^{-6}	90.6
		Flow	7.60×10^{-7}		
Ocean acidification	mol H^+ equiv	Batch	1.44×10^{-5}	1.32×10^{-5}	91.5
		Flow	1.22×10^{-6}		
P. cycle	kg P equiv	Batch	2.40×10^{-5}	1.96×10^{-5}	81.6
		Flow	4.41×10^{-6}		
N. cycle	kg N equiv	Batch	1.70×10^{-6}	1.63×10^{-6}	96.2
		Flow	6.52×10^{-8}		
Blue water	kg 1,4-DCB	Batch	3.92×10^{-6}	3.12×10^{-6}	79.6
		Flow	7.99×10^{-7}		
Green water	kg 1,4-DCB	Batch	3.53×10^{-6}	2.55×10^{-6}	72.4
		Flow	9.74×10^{-7}		
Land system change	m^2	Batch	1.48×10^{-3}	2.04×10^{-7}	85.5
		Flow	2.10×10^{-4}		
Functional B.I.	E/MSY	Batch	1.69×10^{-4}	1.59×10^{-4}	93.8
		Flow	1.04×10^{-5}		
Genetic B.I.	E/MSY	Batch	2.04×10^{-5}	2.00×10^{-5}	97.9
		Flow	4.37×10^{-7}		

Table S15. Results in the nine planetary boundaries analysis in the phenibut process.

Category	Unit	Method	Results	Absolute reduction	Reduction (%)
CO_2 concentration	ton CO_2 equiv	Batch	4.30×10^{-4}	4.10×10^{-1}	95.2
		Flow	2.00×10^{-5}		
Radiative forcing	kBq Co-60 equiv	Batch	3.46×10^{-5}	3.34×10^{-5}	96.5
		Flow	1.21×10^{-6}		
S. ozone depletion	kg CFC11 equiv	Batch	1.92×10^{-6}	1.83×10^{-6}	95.4
		Flow	8.92×10^{-8}		
A. aerosol loading	kg PM2.5 equiv	Batch	1.71×10^{-5}	1.63×10^{-5}	95.7
		Flow	7.29×10^{-7}		
Ocean acidification	mol H^+ equiv	Batch	2.77×10^{-5}	2.64×10^{-5}	95.3
		Flow	1.30×10^{-6}		
P. cycle	kg P equiv	Batch	1.04×10^{-4}	1.01×10^{-7}	96.9
		Flow	3.28×10^{-6}		
N. cycle	kg N equiv	Batch	1.02×10^{-5}	1.00×10^{-5}	98.1
		Flow	1.89×10^{-7}		
Blue water	kg 1,4-DCB	Batch	1.01×10^{-5}	9.16×10^{-6}	90.9
		Flow	9.12×10^{-7}		
Green water	kg 1,4-DCB	Batch	1.51×10^{-5}	1.20×10^{-5}	79.5
		Flow	3.08×10^{-6}		
Land system change	m^2	Batch	4.68×10^{-3}	4.53×10^{-3}	96.8
		Flow	1.50×10^{-4}		
Functional B.I.	E/MSY	Batch	2.17×10^{-4}	2.00×10^{-4}	92.2
		Flow	1.70×10^{-5}		
Genetic B.I.	E/MSY	Batch	4.17×10^{-5}	2.60×10^{-5}	62.3
		Flow	1.57×10^{-5}		

4. Amitriptylne hydrochloride results

Table S16. Component-wise e-factor results in the amitriptyline hydrochloride process, comparing traditional batch and continuous-flow methods. THF, n-BuLi, EtOH and HCl represent tetrahydrofuran, n-butyllithium, ethanol and hydrochloric acid, respectively.

Compounds	Waste batch (mol)	Waste flow (mol/h)	Flow duration (h)	E-factor (batch)	E-factor (flow)
1-Bromo-2-bromomethyl	0.010	0.008	0.89	0.25	0.06
Benzene	0.015	0.009	0.89	0.10	0.02
n-BuLi	8.500	3.783	0.89	63.25	7.44
THF	1.230	2.304	0.89	10.94	5.42
n-hexane	0.113	0.026	0.89	1.70	0.10
Grignard reagent	0.417	0.104	0.89	1.98	0.13
EtOH	0.089	0.021	0.89	0.34	0.02
HCl	0.196	0.301	0.89	1.22	0.49
**Total:**				79.78	13.68

Table S17. Component-wise CO_2 emission results in the amitriptyline hydrochloride process, comparing traditional batch and continuous-flow methods. THF, n-BuLi, EtOH and HCl represent tetrahydrofuran, n-butyllithium, ethanol and hydrochloric acid, respectively.

**Compounds**	**Batch (kg CO_2 equiv)**	**Flow (kg CO_2 equiv)**	**Sub-components**	**Batch (kg CO_2 equiv)**	**Flow (kg CO_2 equiv)**
1-bromo-2-bromomethylbenzene	6.13×10^{-3}	6.81×10^{-3}	Benzene	6.67×10^{-4}	6.99×10^{-4}
			Ethylene	3.71×10^{-3}	4.23×10^{-3}
			Bromine	1.76×10^{-3}	1.87×10^{-3}
n-BuLi	1.38×10^{-3}	5.33×10^{-3}	Lithium oxide	1.07×10^{-3}	4.78×10^{-3}
			Butane	3.09×10^{-3}	5.52×10^{-4}
THF	3.91×10^{-1}	2.07×10^{-1}	THF	3.91×10^{-3}	2.07×10^{-1}
Hexane	6.04×10^{-3}	1.35×10^{-2}	Hexane	6.04×10^{-3}	1.35×10^{-2}
Grignard reagent	1.52×10^{-3}	4.57×10^{-4}	Dimethylamine	1.14×10^{-3}	3.42×10^{-4}
			Propane	3.83×10^{-4}	1.15×10^{-4}
			$MgCl_2$	0	0
Ethanol	2.53×10^{-3}	7.51×10^{-3}	Ethanol	2.53×10^{-3}	7.51×10^{-4}
HCl	2.82×10^{-4}	7.59×10^{-5}	HCl	2.82×10^{-4}	7.59×10^{-5}
Isopropanol	2.46×10^{-3}	4.47×10^{-3}	Isopropanol	2.46×10^{-3}	4.47×10^{-3}
Electricity	1.03×10^{-1}	4.56×10^{-2}	Electricity	1.03×10^{-1}	4.56×10^{-2}
**Total:**	5.14×10^{-1}	2.84×10^{-1}			

Table S18. Component-wise water consumption results in the amitriptyline hydrochloride process, comparing traditional batch and continuous-flow methods. THF, n-BuLi, EtOH and HCl represent tetrahydrofuran, n-butyllithium, ethanol and hydrochloric acid, respectively.

**Compounds**	**Batch (m^3)**	**Flow (m^3)**	**Sub-components**	**Batch (m^3)**	**Flow (m^3)**
1-bromo-2-bromomethylbenzene	4.60×10^{-5}	5.36×10^{-5}	Benzene	9.78×10^{-6}	1.16×10^{-5}
			Ethylene	2.86×10^{-5}	3.34×10^{-5}
			Bromine	7.65×10^{-6}	8.51×10^{-6}
n-BuLi	1.64×10^{-3}	6.21×10^{-5}	Lithium oxide	1.64×10^{-3}	5.99×10^{-5}
			Butane	1.17×10^{-6}	2.14×10^{-6}
THF	2.10×10^{-2}	1.14×10^{-2}	THF	2.10×10^{-2}	1.14×10^{-2}
Hexane	9.41×10^{-5}	2.15×10^{-4}	Hexane	9.41×10^{-5}	2.15×10^{-4}
Grignard reagent	1.64×10^{-5}	5.02×10^{-6}	Dimethylamine	1.51×10^{-5}	4.62×10^{-6}
			Propane	1.30×10^{-6}	3.97×10^{-7}
			$MgCl_2$	0	0
Ethanol	9.70×10^{-4}	2.94×10^{-4}	Ethanol	9.70×10^{-4}	2.94×10^{-4}
HCl	1.03×10^{-3}	2.85×10^{-4}	HCl	1.03×10^{-3}	2.85×10^{-4}
Isopropanol	2.07×10^{-5}	3.86×10^{-5}	Isopropanol	2.07×10^{-5}	3.86×10^{-5}
Electricity	5.77×10^{-1}	2.62×10^{-1}	Electricity	5.77×10^{-1}	2.62×10^{-1}
**Total:**	0.602	0.274			

Table S19. Component-wise land system change results in the amitriptyline hydrochloride process, comparing traditional batch and continuous-flow methods. THF, n-BuLi, EtOH and HCl represent tetrahydrofuran, n-butyllithium, ethanol and hydrochloric acid, respectively.

**Compounds**	**Batch ($\times10^{-3}$ m^2)**	**Flow ($\times10^{-3}$ m^2)**	**Sub-components**	**Batch ($\times10^{-3}$ m^2)**	**Flow ($\times10^{-3}$ m^2)**
1-bromo-2-bromomethylbenzene	8.25×10^{-2}	9.07×10^{-2}	Benzene	2.90×10^{-3}	1.16×10^{-3}
			Ethylene	5.64×10^{-2}	6.47×10^{-2}
			Bromine	2.32×10^{-2}	2.48×10^{-2}
n-BuLi	4.60×10^{-2}	1.54×10^{-1}	Lithium oxide	3.65×10^{-2}	1.37×10^{-1}
			Butane	9.45×10^{-3}	1.70×10^{-2}
THF	$1.01\times10^{+1}$	5.36×10^{0}	THF	$1.01\times10^{+1}$	5.36×10^{0}
Hexane	4.83×10^{-1}	1.09×10^{0}	Hexane	4.83×10^{-1}	1.09×10^{0}
Grignard reagent	2.96×10^{-2}	8.92×10^{-3}	Dimethylamine	1.78×10^{-2}	5.37×10^{-3}
			Propane	1.18×10^{-2}	3.55×10^{-3}
			$MgCl_2$	0	0
Ethanol	4.33×10^{0}	1.29×10^{0}	Ethanol	4.33×10^{0}	1.29×10^{0}
HCl	4.70×10^{-3}	1.27×10^{-3}	HCl	4.70×10^{-3}	1.27×10^{-3}
Isopropanol	2.37×10^{-2}	4.34×10^{-2}	Isopropanol	2.37×10^{-2}	4.34×10^{-2}
Electricity	1.36×10^{-1}	6.08×10^{-2}	Electricity	1.36×10^{-1}	6.08×10^{-2}
**Total:**	15.20	8.09			

Table S20. Component-wise environmental impact assessment results in the amitriptyline hydrochloride manufacturing process considering batch method. THF, n-BuLi, EtOH, HCl, Grig., Isop., and Elec. represent tetrahydrofuran, n-butyllithium, ethanol, hydrochloric acid, Grignard reagent, isopropanol and electricity respectively.

Impact category	Unit	1-bromo	n-BuLi	THF	Hexane	Grig	EtOH	HCl	Isop.	Elec.
Global warming	kg CO_2 equiv	6.13×10^{-3}	1.38×10^{-3}	3.91×10^{-3}	6.04×10^{-3}	1.52×10^{-3}	2.53×10^{-3}	2.82×10^{-4}	2.4×10^{-3}	1.03×10^{-1}
Ozone depletion	kg CFC11 equiv	3.00×10^{-8}	6.04×10^{-9}	2.36×10^{-6}	1.27×10^{-7}	1.05×10^{-8}	4.18×10^{-7}	3.74×10^{-9}	5.07×10^{-9}	3.86×10^{-7}
Ionizing radiation	kBq Co-60 equiv	5.02×10^{-7}	4.00×10^{-8}	6.29×10^{-5}	7.22×10^{-7}	1.52×10^{-7}	8.80×10^{-9}	2.12×10^{-8}	1.93×10^{-7}	2.86×10^{-6}
Ozone form. hum.	kg NO_x equiv	4.59×10^{-7}	1.93×10^{-7}	4.31×10^{-5}	1.65×10^{-6}	1.28×10^{-7}	3.07×10^{-7}	2.90×10^{-8}	2.21×10^{-7}	5.24×10^{-6}
Particulate matter	kg PM2.5 equiv	2.73×10^{-7}	8.30×10^{-8}	2.56×10^{-5}	5.48×10^{-7}	6.13×10^{-8}	1.62×10^{-7}	1.73×10^{-8}	9.35×10^{-8}	1.55×10^{-6}
Ozone form. ter.	kg NO_x equiv	5.50×10^{-7}	2.28×10^{-7}	5.17×10^{-5}	2.37×10^{-6}	1.59×10^{-7}	3.76×10^{-7}	3.40×10^{-8}	2.97×10^{-7}	6.17×10^{-6}
Ter. acidification	kg SO_2 equiv	4.32×10^{-7}	1.41×10^{-7}	3.48×10^{-5}	9.61×10^{-7}	1.10×10^{-7}	5.43×10^{-7}	3.11×10^{-8}	1.74×10^{-7}	3.02×10^{-6}
Water eutrophication	kg P equiv	1.95×10^{-6}	5.48×10^{-7}	2.11×10^{-4}	5.60×10^{-6}	5.39×10^{-7}	9.82×10^{-7}	9.51×10^{-8}	7.75×10^{-7}	1.13×10^{-6}
Marine eutrophication	kg N equiv	2.02×10^{-8}	3.92×10^{-9}	2.06×10^{-6}	1.79×10^{-8}	4.44×10^{-7}	1.84×10^{-6}	1.42×10^{-9}	4.30×10^{-9}	3.80×10^{-7}
Terrestrial ecotoxicity	kg 1,4-DCB	5.40×10^{-8}	2.31×10^{-7}	6.34×10^{-6}	2.54×10^{-7}	6.18×10^{-9}	5.06×10^{-7}	9.78×10^{-8}	1.52×10^{-8}	1.50×10^{-6}
Water ecotoxicity	kg 1,4-DCB	1.69×10^{-7}	7.86×10^{-7}	2.07×10^{-5}	3.94×10^{-7}	7.07×10^{-8}	1.92×10^{-5}	4.37×10^{-7}	4.68×10^{-8}	2.23×10^{-7}
Marine ecotoxicity	kg 1,4-DCB	1.51×10^{-7}	6.77×10^{-7}	1.81×10^{-5}	3.76×10^{-7}	5.63×10^{-8}	2.77×10^{-6}	3.69×10^{-7}	4.17×10^{-8}	4.72×10^{-7}
Carcinogenic toxicity hum.	kg 1,4-DCB	1.47×10^{-7}	6.76×10^{-7}	3.78×10^{-5}	1.29×10^{-7}	3.00×10^{-8}	8.43×10^{-7}	3.43×10^{-7}	1.78×10^{-8}	5.46×10^{-6}
Non-carcinogenic toxicity hum.	kg 1,4-DCB	1.06×10^{-9}	3.28×10^{-8}	1.13×10^{-7}	4.16×10^{-9}	4.12×10^{-10}	5.07×10^{-8}	1.78×10^{-8}	$3.73 \times^{10-10}$	-1.34×10^{-8}
Land use	m^2	8.25×10^{-5}	4.60×10^{-5}	1.01×10^{-2}	4.83×10^{-4}	2.96×10^{-5}	4.33×10^{-3}	4.70×10^{-6}	2.37×10^{-5}	1.36×10^{-4}
Mineral scarcity	kg Cu equiv	7.37×10^{-11}	3.29×10^{-9}	8.03×10^{-9}	2.34×10^{-10}	3.75×10^{-11}	2.34×10^{-11}	1.39×10^{-11}	3.32×10^{-11}	1.87×10^{-10}
Fossil scarcity	kg oil equiv	2.28×10^{-6}	5.76×10^{-7}	1.44×10^{-4}	9.81×10^{-6}	1.17×10^{-6}	3.80×10^{-7}	7.09×10^{-8}	1.66×10^{-6}	2.77×10^{-5}
Water consumption	m^3	4.60×10^{-5}	1.64×10^{-3}	2.10×10^{-2}	9.41×10^{-5}	1.64×10^{-5}	9.70×10^{-4}	1.03×10^{-3}	2.07×10^{-5}	5.77×10^{-1}

Table S21. Component-wise environmental impact assessment results in the amitriptyline hydrochloride manufacturing process considering continuous-flow method. THF, n-BuLi, EtOH, HCl, Grig., Isop., and Elec. represent tetrahydrofuran, n-butyllithium, ethanol, hydrochloric acid, Grignard reagent, isopropanol and electricity respectively.

Impact category	Unit	1-bromo	n-BuLi	THF	Hexane	Grig	EtOH	HCl	Isop.	Elec.
Global warming	kg CO_2 equiv	6.81×10^{-3}	5.33×10^{-3}	2.07×10^{-1}	1.35×10^{-2}	4.57×10^{-4}	7.51×10^{-4}	7.59×10^{-5}	4.47×10^{-3}	4.56×10^{-2}
Ozone depletion	kg CFC11 equiv	3.33×10^{-8}	3.53×10^{-8}	1.27×10^{-6}	2.91×10^{-7}	3.20×10^{-9}	1.27×10^{-7}	1.03×10^{-9}	9.43×10^{-9}	1.75×10^{-7}
Ionizing radiation	kBq Co-60 equiv	6.73×10^{-7}	4.24×10^{-7}	3.40×10^{-5}	1.65×10^{-6}	4.66×10^{-8}	2.67×10^{-9}	5.84×10^{-9}	3.60×10^{-7}	1.30×10^{-6}
Ozone form. hum.	kg NO_x equiv	5.06×10^{-7}	8.26×10^{-7}	2.33×10^{-5}	3.76×10^{-6}	3.93×10^{-8}	9.31×10^{-8}	7.98×10^{-9}	4.12×10^{-7}	2.37×10^{-6}
Particulate matter	kg PM2.5 equiv	2.75×10^{-7}	4.50×10^{-7}	1.39×10^{-5}	1.25×10^{-6}	1.88×10^{-8}	4.90×10^{-8}	4.75×10^{-9}	1.74×10^{-7}	7.01×10^{-7}
Ozone form. ter.	kg NO_x equiv	6.12×10^{-7}	9.72×10^{-7}	2.79×10^{-5}	5.42×10^{-6}	4.86×10^{-8}	1.14×10^{-7}	9.35×10^{-9}	5.52×10^{-7}	2.79×10^{-6}
Ter. acidification	kg SO_2 equiv	4.58×10^{-7}	7.18×10^{-7}	1.88×10^{-5}	2.20×10^{-6}	3.38×10^{-8}	1.65×10^{-7}	8.54×10^{-9}	3.25×10^{-7}	1.37×10^{-6}
Water eutrophication	kg P equiv	2.32×10^{-6}	7.44×10^{-6}	1.14×10^{-4}	1.28×10^{-5}	1.65×10^{-7}	2.98×10^{-7}	2.62×10^{-8}	1.44×10^{-6}	5.13×10^{-7}
Marine eutrophication	kg N equiv	2.39×10^{-8}	7.08×10^{-7}	1.11×10^{-6}	4.10×10^{-8}	1.36×10^{-7}	5.57×10^{-7}	3.90×10^{-10}	8.01×10^{-9}	1.72×10^{-7}
Terrestrial ecotoxicity	kg 1,4-DCB	3.73×10^{-7}	9.54×10^{-8}	3.43×10^{-6}	5.81×10^{-7}	1.89×10^{-9}	1.53×10^{-7}	2.69×10^{-8}	2.83×10^{-8}	6.79×10^{-7}
Water ecotoxicity	kg 1,4-DCB	1.55×10^{-6}	3.31×10^{-7}	1.12×10^{-5}	8.99×10^{-7}	2.17×10^{-8}	5.82×10^{-6}	1.20×10^{-7}	8.72×10^{-8}	1.01×10^{-7}
Marine ecotoxicity	kg 1,4-DCB	1.25×10^{-6}	2.91×10^{-7}	9.80×10^{-6}	8.60×10^{-7}	1.73×10^{-8}	8.40×10^{-7}	1.01×10^{-7}	7.76×10^{-8}	2.14×10^{-7}
Carcinogenic toxicity hum.	kg 1,4-DCB	5.63×10^{-6}	9.16×10^{-8}	2.04×10^{-5}	2.95×10^{-7}	9.20×10^{-9}	2.55×10^{-7}	9.42×10^{-8}	3.32×10^{-8}	2.47×10^{-6}
Non-carcinogenic toxicity hum.	kg 1,4-DCB	2.85×10^{-8}	2.92×10^{-9}	6.09×10^{-8}	9.50×10^{-9}	1.26×10^{-10}	1.54×10^{-8}	4.91×10^{-9}	6.94×10^{-10}	-6.05×10^{-9}
Land use	m^2	9.07×10^{-5}	1.54×10^{-4}	5.36×10^{-3}	1.09×10^{-3}	8.92×10^{-6}	1.29×10^{-3}	1.27×10^{-6}	4.34×10^{-5}	6.08×10^{-5}
Mineral scarcity	kg Cu equiv	8.78×10^{-11}	2.25×10^{-9}	4.34×10^{-9}	5.35×10^{-10}	1.15×10^{-11}	7.09×10^{-12}	3.81×10^{-12}	6.18×10^{-11}	8.48×10^{-11}
Fossil scarcity	kg oil equiv	2.71×10^{-6}	1.86×10^{-6}	7.80×10^{-5}	2.24×10^{-5}	3.59×10^{-7}	1.15×10^{-7}	1.95×10^{-8}	3.09×10^{-6}	1.25×10^{-5}
Water consumption	m^3	5.36×10^{-5}	6.21×10^{-5}	1.14×10^{-2}	2.15×10^{-4}	5.02×10^{-6}	2.94×10^{-4}	2.85×10^{-4}	3.86×10^{-5}	2.62×10^{-1}

Table S22. Component-wise endpoint results in the amitriptyline hydrochloride manufacturing process considering batch method.

Damage category	Unit	1-bromo	n-BuLi	THF	Hexane	Grig	EtOH	HCl	Isop.	Elec.
Human health	DALY	1.02×10^{-8}	2.89×10^{-9}	8.22×10^{-7}	1.47×10^{-8}	2.43×10^{-9}	5.73×10^{-9}	6.78×10^{-10}	3.83×10^{-9}	1.29×10^{-7}
Ecosystems	Species.yr	2.44×10^{-11}	6.57×10^{-12}	1.98×10^{-9}	3.89×10^{-11}	6.27×10^{-12}	5.88×10^{-11}	1.29×10^{-12}	9.90×10^{-12}	3.57×10^{-10}
Resources	USD2013	7.68×10^{-4}	2.83×10^{-4}	4.07×10^{-2}	4.21×10^{-3}	3.98×10^{-4}	1.51×10^{-4}	2.30×10^{-5}	6.56×10^{-4}	8.77×10^{-3}

Table S23. Component-wise endpoint results in the amitriptyline hydrochloride manufacturing process considering continuous-flow method.

Damage category	Unit	1-bromo	n-BuLi	THF	Hexane	Grig	EtOH	HCl	Isop.	Elec.
Human health	DALY	5.88×10^{-9}	6.19×10^{-9}	4.44×10^{-7}	3.35×10^{-8}	7.44×10^{-10}	1.74×10^{-9}	1.87×10^{-10}	7.13×10^{-9}	5.82×10^{-8}
Ecosystems	Species.yr	1.38×10^{-11}	1.47×10^{-11}	1.07×10^{-9}	8.88×10^{-11}	1.92×10^{-12}	1.78×10^{-11}	3.54×10^{-13}	1.84×10^{-11}	1.61×10^{-10}
Resources	USD2013	4.89×10^{-4}	3.04×10^{-4}	2.20×10^{-2}	9.63×10^{-3}	1.22×10^{-4}	4.59×10^{-5}	6.33×10^{-6}	1.22×10^{-3}	3.97×10^{-3}

5. Tamoxifen results

Table S24. Component-wise e-factor results in the tamoxifen process, comparing traditional batch and continuous-flow methods. n-BuLi, TFAA, Et_3N, and THF represent n-butyllithium, trifluoroacetic anhydride, triethylamine and tetrahydrofuran, respectively.

**Compounds**	**Waste batch (mol)**	**Waste flow (mol/h)**	**Flow duration (h)**	**E-factor (batch)**	**E-factor (flow)**
Weinreb amide	0.003	0.000	0.81	0.09	0.00
Grignard reagent	0.070	0.030	0.81	1.76	0.49
Aryl-bromide	0.002	0.000	0.81	0.04	0.00
n-BuLi	0.045	0.006	0.81	0.40	0.03
TFAA	0.050	0.031	0.81	1.46	0.60
Et_3N	0.034	0.024	0.81	0.48	0.22
THF	8.876	3.317	0.81	88.90	21.72
**Total:**				93.13	23.06

Table S25. Component-wise CO_2 emission results in the tamoxifen process, comparing traditional batch and continuous-flow methods. n-BuLi, TFAA, Et_3N, and THF represent n-butyllithium, trifluoroacetic anhydride, triethylamine and tetrahydrofuran, respectively.

**Compounds**	**Batch (kg CO_2 equiv)**	**Flow (kg CO_2 equiv)**	**Sub-components**	**Batch (kg CO_2 equiv)**	**Flow (kg CO_2 equiv)**
Weinreb Amide	4.45×10^{-3}	1.55×10^{-3}	Benzene	1.34×10^{-3}	4.67×10^{-4}
			Acetone	1.17×10^{-3}	4.09×10^{-4}
			Butane	4.30×10^{-4}	1.50×10^{-4}
			Ethylamine	1.51×10^{-3}	5.25×10^{-4}
Grignard reagent	2.16×10^{-3}	7.53×10^{-4}	Benzene	2.16×10^{-3}	7.53×10^{-4}
			$MgBr_2$	0	0
n-BuLi	3.27×10^{-3}	1.35×10^{-3}	Lithium Oxide	2.94×10^{-3}	1.21×10^{-3}
			Butane	3.39×10^{-4}	1.40×10^{-4}
Aryl bromide	1.38×10^{-3}	1.68×10^{-3}	Benzene	2.92×10^{-4}	3.57×10^{-4}
			Ethanol	1.05×10^{-4}	1.28×10^{-4}
			Dimethylamine	2.14×10^{-4}	2.61×10^{-4}
			Br_2	7.70×10^{-4}	9.39×10^{-4}
TFAA	1.15×10^{-2}	8.14×10^{-3}	Trifluoromethane	9.30×10^{-3}	6.58×10^{-3}
			Acetone	2.21×10^{-3}	1.56×10^{-3}
Et_3N	3.61×10^{-3}	2.72×10^{-3}	Et_3N	3.61×10^{-3}	2.72×10^{-3}
THF	5.50×10^{-1}	1.67×10^{-1}	THF	5.50×10^{-1}	1.67×10^{-1}
Electricity	1.9×10^{0}	2.69×10^{-2}	Electricity	1.9×10^{0}	2.69×10^{-2}
**Total:**	2.48	0.21			

Table S26. Component-wise water consumption results in the tamoxifen process, comparing traditional batch and continuous-flow methods. n-BuLi, TFAA, Et_3N, and THF represent n-butyllithium, trifluoroacetic anhydride, triethylamine and tetrahydrofuran, respectively.

Compounds	Batch (m^3)	Flow (m^3)	Sub-components	Batch (m^3)	Flow (m^3)
Weinreb Amide	5.41×10^{-5}	1.86×10^{-5}	Benzene	1.97×10^{-5}	6.77×10^{-6}
			Acetone	1.45×10^{-5}	5.00×10^{-6}
			Butane	1.63×10^{-6}	5.62×10^{-7}
			Ethylamine	1.84×10^{-5}	6.32×10^{-6}
Grignard reagent	3.17×10^{-5}	1.09×10^{-5}	Benzene	3.17×10^{-5}	1.09×10^{-5}
			$MgBr_2$	0	0
n-BuLi	3.73×10^{-5}	1.52×10^{-5}	Lithium Oxide	3.60×10^{-5}	1.47×10^{-5}
			Butane	1.29×10^{-6}	5.25×10^{-7}
Aryl bromide	1.14×10^{-5}	1.37×10^{-5}	Benzene	4.28×10^{-6}	5.16×10^{-6}
			Ethanol	9.26×10^{-7}	1.12×10^{-6}
			Dimethylamine	2.82×10^{-6}	3.40×10^{-6}
			Br_2	3.35×10^{-6}	4.03×10^{-6}
TFAA	1.38×10^{-4}	9.62×10^{-5}	Trifluoromethane	1.10×10^{-4}	7.71×10^{-5}
			Acetone	2.73×10^{-5}	1.91×10^{-5}
Et_3N	3.69×10^{-5}	2.75×10^{-5}	Et_3N	3.69×10^{-5}	2.75×10^{-5}
THF	2.96×10^{-2}	8.87×10^{-3}	THF	2.96×10^{-2}	8.87×10^{-3}
Electricity	$1.06\times10^{+1}$	1.49×10^{-1}	Electricity	$1.06\times10^{+1}$	1.49×10^{-1}
**Total:**	10.700	0.158			

Table S27. Component-wise land system change results in the tamoxifen process, comparing traditional batch and continuous-flow methods. n-BuLi, TFAA, Et_3N, and THF represent n-butyllithium, trifluoroacetic anhydride, triethylamine and tetrahydrofuran, respectively.

**Compounds**	**Batch ($\times 10^{-3}$ m^2)**	**Flow ($\times 10^{-3}$ m^2)**	**Sub-components**	**Batch ($\times 10^{-3}$ m^2)**	**Flow ($\times 10^{-3}$ m^2)**
Weinreb Amide	4.62×10^{-2}	1.59×10^{-2}	Benzene	5.83×10^{-3}	2.01×10^{-3}
			Acetone	4.65×10^{-3}	1.60×10^{-3}
			Butane	1.32×10^{-2}	4.53×10^{-3}
			Ethylamine	2.25×10^{-2}	7.76×10^{-3}
Grignard reagent	9.40×10^{-3}	3.24×10^{-3}	Benzene	9.40×10^{-3}	3.24×10^{-3}
			$MgBr_2$	0	0
n-BuLi	9.41×10^{-2}	3.83×10^{-2}	Lithium Oxide	8.37×10^{-2}	3.41×10^{-2}
			Butane	1.04×10^{-2}	4.23×10^{-3}
Aryl bromide	1.61×10^{-2}	1.94×10^{-2}	Benzene	1.27×10^{-3}	1.53×10^{-3}
			Ethanol	1.34×10^{-3}	1.61×10^{-3}
			Dimethylamine	3.34×10^{-3}	4.03×10^{-3}
			Br_2	1.02×10^{-2}	1.23×10^{-2}
TFAA	1.64×10^{-1}	1.14×10^{-1}	Trifluoromethane	1.55×10^{-1}	1.08×10^{-1}
			Acetone	8.75×10^{-3}	6.11×10^{-3}
Et_3N	5.29×10^{-2}	3.94×10^{-2}	Et_3N	5.29×10^{-2}	3.94×10^{-2}
THF	$1.42\times10^{+1}$	4.26×10^{0}	THF	$1.42\times10^{+1}$	4.26×10^{0}
Electricity	2.53×10^{0}	3.53×10^{-2}	Electricity	2.53×10^{0}	3.53×10^{-2}
**Total:**	17.10	4.53			

Table S28. Component-wise environmental impact assessment results in the tamoxifen manufacturing process considering batch method. n-BuLi, TFAA, Et_3N, THF, W. amide represent n-butyllithium, trifluoroacetic anhydride, triethylamine, tetrahydrofuran and Weinreb amide, respectively.

Impact category	Unit	W. amide	Grig	n-BuLi	Aryl-bromide	TFAA	Et_3N	THF	Elec.
Global warming	kg CO_2 equiv	4.45×10^{-3}	2.16×10^{-3}	3.27×10^{-3}	1.38×10^{-3}	1.15×10^{-2}	3.61×10^{-3}	5.50×10^{-1}	1.90×10^{0}
Ozone depletion	kg CFC11 equiv	1.09×10^{-8}	1.71×10^{-9}	2.12×10^{-8}	5.65×10^{-9}	2.93×10^{-5}	1.27×10^{-8}	3.32×10^{-6}	7.14×10^{-6}
Ionizing radiation	kBq Co-60 equiv	1.53×10^{-7}	9.73×10^{-9}	2.55×10^{-7}	7.43×10^{-8}	1.35×10^{-6}	1.61×10^{-7}	8.85×10^{-5}	5.29×10^{-5}
Ozone form. hum.	kg NO_x equiv	4.58×10^{-7}	2.14×10^{-7}	4.96×10^{-7}	1.07×10^{-7}	8.33×10^{-7}	3.72×10^{-7}	6.06×10^{-5}	9.68×10^{-5}
Particulate matter	kg PM2.5 equiv	2.11×10^{-7}	1.11×10^{-7}	2.70×10^{-7}	5.78×10^{-8}	6.06×10^{-7}	1.58×10^{-7}	3.60×10^{-5}	2.86×10^{-5}
Ozone form. ter.	kg NO_x equiv	5.80×10^{-7}	2.75×10^{-7}	5.84×10^{-7}	1.31×10^{-7}	1.01×10^{-6}	4.57×10^{-7}	7.27×10^{-5}	1.14×10^{-4}
Ter. acidification	kg SO_2 equiv	3.52×10^{-7}	1.56×10^{-7}	4.31×10^{-7}	9.30×10^{-8}	1.02×10^{-6}	2.81×10^{-7}	4.89×10^{-5}	5.58×10^{-5}
Water eutrophication	kg P equiv	1.72×10^{-6}	4.99×10^{-7}	4.47×10^{-6}	4.18×10^{-7}	4.78×10^{-6}	2.87×10^{-6}	2.97×10^{-4}	2.09×10^{-5}
Marine eutrophication	kg N equiv	1.91×10^{-7}	4.82×10^{-9}	4.25×10^{-7}	8.59×10^{-8}	4.12×10^{-8}	2.07×10^{-7}	2.89×10^{-6}	7.02×10^{-6}
Terrestrial ecotoxicity	kg 1,4-DCB	1.53×10^{-8}	3.70×10^{-9}	5.73×10^{-8}	9.64×10^{-9}	6.07×10^{-8}	2.00×10^{-8}	8.92×10^{-6}	2.77×10^{-5}
Water ecotoxicity	kg 1,4-DCB	1.46×10^{-7}	2.93×10^{-8}	1.99×10^{-7}	3.38×10^{-8}	4.68×10^{-7}	2.21×10^{-7}	2.91×10^{-5}	4.13×10^{-6}
Marine ecotoxicity	kg 1,4-DCB	1.21×10^{-7}	2.69×10^{-8}	1.74×10^{-7}	2.97×10^{-8}	3.95×10^{-7}	1.81×10^{-7}	2.55×10^{-5}	8.73×10^{-6}
Carcinogenic toxicity hum.	kg 1,4-DCB	1.84×10^{-7}	3.66×10^{-8}	5.50×10^{-8}	2.87×10^{-8}	5.24×10^{-5}	3.55×10^{-8}	5.32×10^{-5}	1.01×10^{-4}
Non-carcinogenic toxicity hum.	kg 1,4-DCB	7.19×10^{-10}	9.98×10^{-11}	1.75×10^{-9}	2.02×10^{-10}	2.09×10^{-7}	1.05×10^{-9}	1.59×10^{-7}	-2.47×10^{-7}
Land use	m^2	4.62×10^{-5}	9.40×10^{-6}	9.41×10^{-5}	1.61×10^{-5}	1.64×10^{-4}	5.29×10^{-5}	1.42×10^{-2}	2.53×10^{-3}
Mineral scarcity	kg Cu equiv	5.70×10^{-11}	3.57×10^{-12}	1.35×10^{-9}	1.46×10^{-11}	4.40×10^{-10}	8.40×10^{-11}	1.13×10^{-8}	3.46×10^{-9}
Fossil scarcity	kg oil equiv	2.82×10^{-6}	1.57×10^{-6}	1.12×10^{-6}	6.73×10^{-7}	3.18×10^{-6}	1.81×10^{-6}	2.03×10^{-4}	5.12×10^{-4}
Water consumption	m^3	5.41×10^{-5}	3.17×10^{-5}	3.73×10^{-5}	1.14×10^{-5}	1.38×10^{-4}	3.69×10^{-5}	2.96×10^{-2}	1.06×10^{1}

Table S29. Component-wise environmental impact assessment results in the tamoxifen manufacturing process considering continuous-flow method. n-BuLi, TFAA, Et_3N, THF, W. amide represent n-butyllithium, trifluoroacetic anhydride, triethylamine, tetrahydrofuran and Weinreb amide, respectively.

Impact category	Unit	W. amide	Grig	n-BuLi	Aryl-bromide	TFAA	Et_3N	THF	Elec.
Global warming	kg CO_2 equiv	1.55×10^{-3}	7.53×10^{-4}	1.35×10^{-3}	1.68×10^{-3}	8.14×10^{-3}	2.72×10^{-3}	1.67×10^{-1}	2.69×10^{-2}
Ozone depletion	kg CFC11 equiv	3.76×10^{-9}	5.92×10^{-10}	8.67×10^{-9}	6.83×10^{-9}	2.05×10^{-5}	9.51×10^{-9}	1.00×10^{-6}	1.00×10^{-7}
Ionizing radiation	kBq Co-60 equiv	5.29×10^{-8}	3.36×10^{-9}	1.04×10^{-7}	8.99×10^{-8}	9.46×10^{-7}	1.20×10^{-7}	2.67×10^{-5}	7.41×10^{-7}
Ozone form. hum.	kg NO_x equiv	1.58×10^{-7}	7.40×10^{-8}	2.03×10^{-7}	1.30×10^{-7}	5.84×10^{-7}	2.78×10^{-7}	1.82×10^{-5}	1.36×10^{-6}
Particulate matter	kg PM2.5 equiv	7.29×10^{-8}	3.82×10^{-8}	1.11×10^{-7}	6.99×10^{-8}	4.25×10^{-7}	1.18×10^{-7}	1.09×10^{-5}	4.01×10^{-7}
Ozone form. ter.	kg NO_x equiv	2.00×10^{-7}	9.51×10^{-8}	2.39×10^{-7}	1.59×10^{-7}	7.11×10^{-7}	3.42×10^{-7}	2.19×10^{-5}	1.60×10^{-6}
Ter. acidification	kg SO_2 equiv	1.21×10^{-7}	5.38×10^{-8}	1.76×10^{-7}	1.12×10^{-7}	7.15×10^{-7}	2.10×10^{-7}	1.47×10^{-5}	7.83×10^{-7}
Water eutrophication	kg P equiv	5.94×10^{-7}	1.72×10^{-7}	1.83×10^{-6}	5.05×10^{-7}	3.35×10^{-6}	2.14×10^{-6}	8.94×10^{-5}	2.93×10^{-7}
Marine eutrophication	kg N equiv	6.59×10^{-8}	1.66×10^{-9}	1.74×10^{-7}	1.04×10^{-7}	2.89×10^{-8}	1.55×10^{-7}	8.71×10^{-7}	9.84×10^{-8}
Terrestrial ecotoxicity	kg 1,4-DCB	5.29×10^{-9}	1.28×10^{-9}	2.35×10^{-8}	1.17×10^{-8}	4.25×10^{-8}	1.50×10^{-8}	2.69×10^{-6}	3.88×10^{-7}
Water ecotoxicity	kg 1,4-DCB	5.05×10^{-8}	1.01×10^{-8}	8.14×10^{-8}	4.09×10^{-8}	3.28×10^{-7}	1.65×10^{-7}	8.77×10^{-6}	5.78×10^{-8}
Marine ecotoxicity	kg 1,4-DCB	4.17×10^{-8}	9.30×10^{-9}	7.14×10^{-8}	3.59×10^{-8}	2.77×10^{-7}	1.36×10^{-7}	7.68×10^{-6}	1.22×10^{-7}
Carcinogenic toxicity hum.	kg 1,4-DCB	6.34×10^{-8}	1.26×10^{-8}	2.25×10^{-8}	3.47×10^{-8}	3.68×10^{-5}	2.65×10^{-8}	1.60×10^{-5}	1.41×10^{-6}
Non-carcinogenic toxicity hum.	kg 1,4-DCB	2.48×10^{-10}	3.45×10^{-11}	7.17×10^{-10}	2.45×10^{-10}	1.47×10^{-7}	7.83×10^{-10}	4.78×10^{-8}	-3.46×10^{-9}
Land use	m^2	1.59×10^{-5}	3.24×10^{-6}	3.83×10^{-5}	1.94×10^{-5}	1.14×10^{-4}	3.94×10^{-5}	4.26×10^{-3}	3.53×10^{-5}
Mineral scarcity	kg Cu equiv	1.97×10^{-11}	1.23×10^{-12}	5.54×10^{-10}	1.76×10^{-11}	3.08×10^{-10}	6.28×10^{-11}	3.40×10^{-9}	4.85×10^{-11}
Fossil scarcity	kg oil equiv	9.75×10^{-7}	5.42×10^{-7}	4.57×10^{-7}	8.13×10^{-7}	2.23×10^{-6}	1.35×10^{-6}	6.11×10^{-5}	7.18×10^{-6}
Water consumption	m^3	1.86×10^{-5}	1.09×10^{-5}	1.52×10^{-5}	1.37×10^{-5}	9.62×10^{-5}	2.75×10^{-5}	8.87×10^{-3}	1.49×10^{-1}

Table S30. Component-wise endpoint results in the tamoxifen manufacturing process considering batch method.

Damage category	Unit	W. amide	Grig	n-BuLi	Aryl-bromide	TFAA	Et_3N	THF	Elec.
Human health	DALY	7.65×10^{-9}	3.86×10^{-9}	7.48×10^{-9}	2.24×10^{-9}	2.49×10^{-8}	5.99×10^{-9}	1.16×10^{-6}	2.37×10^{-6}
Ecosystems	Species.yr	1.88×10^{-11}	8.78×10^{-12}	1.77×10^{-11}	5.46×10^{-12}	4.88×10^{-11}	1.59×10^{-11}	2.78×10^{-9}	6.59×10^{-9}
Resources	USD2013	1.02×10^{-3}	5.86×10^{-4}	3.67×10^{-4}	2.37×10^{-4}	1.06×10^{-3}	6.19×10^{-4}	5.72×10^{-2}	1.62×10^{-1}

Table S31. Component-wise endpoint results in the tamoxifen manufacturing process considering continuous-flow method.

Damage category	Unit	W. amide	Grig	n-BuLi	Aryl-bromide	TFAA	Et_3N	THF	Elec.
Human health	DALY	2.64×10^{-9}	1.33×10^{-9}	3.06×10^{-9}	2.70×10^{-9}	1.75×10^{-8}	4.48×10^{-9}	3.48×10^{-7}	3.33×10^{-8}
Ecosystems	Species.yr	6.48×10^{-12}	3.03×10^{-12}	7.24×10^{-12}	6.60×10^{-12}	3.42×10^{-11}	1.19×10^{-11}	8.38×10^{-10}	9.23×10^{-11}
Resources	USD2013	3.51×10^{-4}	2.03×10^{-4}	1.50×10^{-4}	2.87×10^{-4}	7.43×10^{-2}	4.62×10^{-3}	1.72×10^{-4}	2.27×10^{-4}

6. Zolpidem results

Table 32. Component-wise e-factor results in the zolpidem process, comparing traditional batch and continuous-flow methods. DIPEA, Me_2AlCl and $HN(NME_2)_2$ represent N,N-diisopropylethylamine, dimethylaluminum chloride and secondary amine, respectively.

Compounds	Waste batch (mol)	Waste flow (mol/h)	Flow duration (h)	E-factor (batch)	E-factor (flow)
Phenylacetone	0.000	0.001	0.88	0.00	0.08
Ethyl glyoxalate	0.027	0.004	0.88	1.04	0.26
Toluene	0.376	0.042	0.88	13.14	2.54
Aminopyridine	0.001	0.004	0.88	0.05	0.31
Acetonitrile	0.766	0.100	0.88	11.91	2.72
DIPEA	0.059	0.008	0.88	2.87	0.70
Me_2AlCl	0.039	0.000	0.88	1.77	0.03
$HN(NMe_2)_2$	0.029	0.002	0.88	1.15	0.11
**Total:**				31.94	6.74

Table S33. Component-wise CO_2 emission results in the zolpidem process, comparing traditional batch and continuous-flow methods. DIPEA, Me_2AlCl and $HN(NME_2)_2$ represent N,N-diisopropylethylamine, dimethylaluminum chloride and secondary amine, respectively.

Compounds	Batch (kg CO_2 equiv)	Flow (kg CO_2 equiv)	Sub-components	Batch (kg CO_2 equiv)	Flow (kg CO_2 equiv)
Ketone	3.38×10^{-4}	1.52×10^{-3}	Benzene	1.80×10^{-4}	8.08×10^{-4}
			Acetone	1.58×10^{-4}	7.08×10^{-4}
Ethyl glyoxalate	2.43×10^{-3}	1.63×10^{-3}	Ethanol	4.11×10^{-4}	2.77×10^{-4}
			Acetone	2.01×10^{-3}	1.36×10^{-4}
Toluene	2.18×10^{-2}	5.42×10^{-3}	Toluene	4.66×10^{-4}	3.05×10^{-4}
Aminopyridine	5.70×10^{-4}	3.73×10^{-3}	3-Methylpyridine	1.04×10^{-4}	6.81×10^{-4}
			NH_3	1.86×10^{-3}	9.05×10^{-4}
Acetonitrile	5.98×10^{-2}	1.75×10^{-2}	Acetonitrile	2.36×10^{-4}	1.15×10^{-4}
DIPEA	4.73×10^{-3}	2.30×10^{-3}	Ethane	2.64×10^{-3}	1.28×10^{-3}
			N_2	4.77×10^{-3}	1.40×10^{-3}
			Propane	1.66×10^{-3}	4.84×10^{-4}
Me_2AlCl	6.43×10^{-3}	1.88×10^{-3}	Methyl chloride	2.70×10^{-3}	1.31×10^{-3}
			Aluminum	4.06×10^{-5}	1.97×10^{-5}
Secondary amine	2.74×10^{-3}	1.33×10^{-3}	Dimethylamine	2.18×10^{-2}	5.42×10^{-3}
			N_2	5.98×10^{-2}	1.75×10^{-2}
Electricity	3.33×10^{-3}	7.09×10^{-4}	Electricity	3.33×10^{-3}	7.09×10^{-4}
**Total:**	0.10	0.04			

Table S34. Component-wise water consumption results in the zolpidem process, comparing traditional batch and continuous-flow methods. DIPEA, Me_2AlCl and $HN(NME_2)_2$ represent N,N-diisopropylethylamine, dimethylaluminum chloride and secondary amine, respectively.

**Compounds**	**Batch (m^3)**	**Flow (m^3)**	**Sub-components**	**Batch (m^3)**	**Flow (m^3)**
Ketone	4.27×10^{-6}	2.00×10^{-5}	Benzene	2.46×10^{-6}	1.15×10^{-5}
			Acetone	1.81×10^{-6}	8.51×10^{-6}
Ethyl glyoxalate	2.66×10^{-5}	1.87×10^{-5}	Ethanol	3.39×10^{-6}	2.39×10^{-6}
			Acetone	2.32×10^{-5}	1.63×10^{-5}
Toluene	4.25×10^{-4}	1.10×10^{-4}	Toluene	4.25×10^{-4}	1.10×10^{-4}
Aminopyridine	7.72×10^{-6}	5.29×10^{-5}	3-Methylpyridine	6.62×10^{-6}	4.54×10^{-5}
			NH_3	1.10×10^{-6}	7.54×10^{-6}
Acetonitrile	6.96×10^{-4}	2.13×10^{-4}	Acetonitrile	5.84×10^{-6}	2.97×10^{-6}
DIPEA	1.16×10^{-3}	5.88×10^{-4}	Ethane	1.14×10^{-3}	5.81×10^{-4}
			N_2	8.29×10^{-6}	4.21×10^{-6}
			Propane	1.17×10^{-5}	3.58×10^{-6}
Me_2AlCl	6.59×10^{-5}	2.01×10^{-5}	Methyl chloride	5.41×10^{-5}	1.66×10^{-5}
			Aluminum	3.32×10^{-5}	1.69×10^{-5}
Secondary amine	2.30×10^{-4}	1.17×10^{-4}	Dimethylamine	1.97×10^{-4}	1.00×10^{-4}
			N_2	6.62×10^{-6}	4.54×10^{-5}
Electricity	1.74×10^{-2}	3.87×10^{-3}	Electricity	1.74×10^{-2}	3.87×10^{-3}
**Total:**	0.020	0.005			

Table S35. Component-wise land system change results in the zolpidem process, comparing traditional batch and continuous-flow methods. DIPEA, Me_2AlCl and $HN(NME_2)_2$ represent N,N-diisopropylethylamine, dimethylaluminum chloride and secondary amine, respectively.

Compounds	Batch ($\times 10^{-3}$ m^2)	Flow ($\times 10^{-3}$ m^2)	Sub-components	Batch ($\times 10^{-3}$ m^2)	Flow ($\times 10^{-3}$ m^2)
Ketone	1.39×10^{-3}	6.34×10^{-3}	Benzene	7.74×10^{-4}	3.53×10^{-3}
			Acetone	6.17×10^{-4}	2.81×10^{-3}
Ethyl glyoxalate	1.31×10^{-2}	8.94×10^{-3}	Ethanol	5.21×10^{-3}	3.56×10^{-3}
			Acetone	7.88×10^{-3}	5.38×10^{-3}
Toluene	2.22×10^{-2}	5.61×10^{-3}	Toluene	6.15×10^{-3}	4.09×10^{-2}
Aminopyridine	7.15×10^{-3}	4.75×10^{-2}	3-Methylpyridine	9.96×10^{-4}	6.62×10^{-3}
			NH_3	5.64×10^{-2}	2.78×10^{-2}
Acetonitrile	5.93×10^{-1}	1.76×10^{-1}	Acetonitrile	5.08×10^{-4}	2.50×10^{-4}
DIPEA	1.37×10^{-1}	6.76×10^{-2}	Ethane	8.02×10^{-2}	3.95×10^{-2}
			N_2	2.57×10^{-3}	7.61×10^{-4}
			Propane	8.69×10^{-2}	2.58×10^{-2}
Me_2AlCl	8.95×10^{-2}	2.65×10^{-2}	Methyl chloride	2.57×10^{-3}	7.61×10^{-4}
			Aluminum	8.69×10^{-2}	2.58×10^{-2}
Secondary amine	4.18×10^{-2}	2.06×10^{-2}	Dimethylamine	4.18×10^{-2}	2.06×10^{-2}
			N_2	8.74×10^{-5}	4.31×10^{-5}
Electricity	4.37×10^{-3}	9.44×10^{-1}	Electricity	4.37×10^{-3}	9.44×10^{-4}
**Total:**	0.91	0.36			

Table S36. Component-wise environmental impact assessment results in the zolpidem manufacturing process considering batch method. Keto, Oxal, Tol, A.P., DIPEA, Me_2 AlCl, MeCN, NH and Elec. represent ketone, oxalate, toluene and amino-pyridine, N,N-diisopropylethylamine, dimethylaluminum chloride, acetonitrile, secondary amine and electricity respectively.

Impact category	Unit	Keto	Oxal	Tol	A.P.	MeCN	DIP-EA	Me_2 AlCl	NH	Elec.
Global warming	kg CO_2 equiv	3.38×10^{-4}	2.43×10^{-3}	2.18×10^{-2}	5.70×10^{-4}	5.98×10^{-2}	4.73×10^{-3}	6.43×10^{-3}	2.74×10^{-3}	3.33×10^{-3}
Ozone depletion	kg CFC11 equiv	2.68×10^{-10}	2.24×10^{-9}	3.14×10^{-9}	1.76×10^{-9}	1.11×10^{-7}	4.12×10^{-8}	3.20×10^{-7}	1.67×10^{-8}	1.24×10^{-8}
Ionizing radiation	kBq Co-60 equiv	6.62×10^{-9}	8.83×10^{-8}	2.78×10^{-8}	6.70×10^{-8}	1.46×10^{-6}	4.17×10^{-7}	2.10×10^{-7}	2.86×10^{-7}	9.22×10^{-8}
Ozone form. hum.	kg NO_x equiv	3.55×10^{-8}	2.63×10^{-7}	1.63×10^{-6}	7.16×10^{-8}	4.23×10^{-6}	5.59×10^{-7}	1.18×10^{-6}	1.97×10^{-7}	1.69×10^{-7}
Particulate matter	kg PM2.5 equiv	1.59×10^{-8}	9.97×10^{-8}	5.26×10^{-7}	3.44×10^{-8}	2.43×10^{-6}	3.01×10^{-7}	5.27×10^{-7}	8.61×10^{-8}	4.99×10^{-8}
Ozone form. ter.	kg NO_x equiv	4.60×10^{-8}	3.42×10^{-7}	2.04×10^{-6}	1.03×10^{-7}	5.16×10^{-6}	6.84×10^{-7}	1.40×10^{-6}	2.44×10^{-7}	1.99×10^{-7}
Ter. acidification	kg SO_2 equiv	2.58×10^{-8}	1.90×10^{-7}	9.96×10^{-7}	8.48×10^{-8}	5.81×10^{-6}	4.54×10^{-7}	8.59×10^{-7}	1.73×10^{-7}	9.74×10^{-8}
Water eutrophication	kg P equiv	7.32×10^{-8}	6.49×10^{-7}	4.40×10^{-7}	2.36×10^{-7}	2.75×10^{-5}	1.79×10^{-6}	2.14×10^{-6}	9.10×10^{-7}	3.65×10^{-8}
Marine eutrophication	kg N equiv	5.51×10^{-10}	2.50×10^{-9}	4.94×10^{-9}	2.02×10^{-9}	1.10×10^{-5}	2.00×10^{-8}	3.81×10^{-8}	1.04×10^{-6}	1.22×10^{-8}
Terrestrial ecotoxicity	kg 1,4-DCB	5.80×10^{-10}	8.69×10^{-9}	8.02×10^{-9}	5.10×10^{-8}	1.81×10^{-7}	3.77×10^{-8}	2.62×10^{-8}	8.32×10^{-9}	4.83×10^{-8}
Water ecotoxicity	kg 1,4-DCB	4.33×10^{-9}	3.39×10^{-8}	4.83×10^{-8}	6.52×10^{-7}	1.57×10^{-6}	3.62×10^{-7}	7.99×10^{-7}	9.44×10^{-8}	7.20×10^{-9}
Marine ecotoxicity	kg 1,4-DCB	3.54×10^{-9}	2.58×10^{-8}	4.10×10^{-8}	1.03×10^{-7}	1.32×10^{-6}	3.04×10^{-7}	6.52×10^{-7}	7.25×10^{-8}	1.52×10^{-8}
Carcinogenic toxicity hum.	kg 1,4-DCB	2.08×10^{-8}	2.29×10^{-7}	2.92×10^{-8}	5.34×10^{-9}	3.60×10^{-7}	1.51×10^{-7}	4.61×10^{-7}	4.53×10^{-8}	1.76×10^{-7}
Non-carcinogenic toxicity hum.	kg 1,4-DCB	1.96×10^{-11}	2.33×10^{-10}	3.42×10^{-10}	6.76×10^{-7}	8.99×10^{-9}	2.03×10^{-9}	5.00×10^{-9}	6.10×10^{-10}	4.30×10^{-10}
Land use	m^2	1.39×10^{-6}	1.31×10^{-5}	2.22×10^{-5}	7.15×10^{-6}	5.93×10^{-4}	1.37×10^{-4}	8.95×10^{-5}	4.18×10^{-5}	4.37×10^{-6}
Mineral scarcity	kg Cu equiv	1.17×10^{-12}	1.90×10^{-11}	1.63×10^{-11}	8.87×10^{-12}	8.12×10^{-10}	1.61×10^{-10}	7.24×10^{-10}	5.62×10^{-11}	6.03×10^{-12}
Fossil scarcity	kg oil equiv	2.24×10^{-7}	1.53×10^{-6}	1.82×10^{-5}	2.58×10^{-7}	2.93×10^{-5}	5.04×10^{-6}	1.76×10^{-6}	1.76×10^{-6}	8.93×10^{-7}
Water consumption	m^3	4.27×10^{-6}	2.66×10^{-5}	4.25×10^{-4}	7.72×10^{-6}	0	1.16×10^{-3}	6.59×10^{-5}	2.30×10^{-4}	1.74×10^{-2}

Table S37. Component-wise environmental impact assessment results in the zolpidem manufacturing process considering continuous-flow method. Keto, Oxal, Tol, A.P., DIPEA, Me_2 AlCl, MeCN, NH and Elec. represent ketone, oxalate, toluene and amino-pyridine, N,N-diisopropylethylamine, dimethylaluminum chloride, acetonitrile, secondary amine and electricity respectively.

**Impact category**	**Unit**	**Keto**	**Oxal**	**Tol**	**A.P.**	**MeCN**	**DIP-EA**	**Me_2 AlCl**	**NH**	**Elec.**
Global warming	kg CO_2 equiv	1.52 ×10^{-3}	1.63 ×10^{-3}	5.42 ×10^{-3}	3.73 ×10^{-3}	1.75 ×10^{-2}	2.30 ×10^{-3}	1.88 ×10^{-3}	1.33 ×10^{-3}	7.09 ×10^{-4}
Ozone depletion	kg CFC11 equiv	1.21 ×10^{-9}	1.52 ×10^{-9}	7.85 ×10^{-10}	1.16 ×10^{-8}	3.28 ×10^{-8}	2.02 ×10^{-8}	9.41 ×10^{-8}	8.19 ×10^{-9}	2.67 ×10^{-9}
Ionizing radiation	kBq Co-60 equiv	2.99 ×10^{-8}	5.98 ×10-8	6.96 ×10^{-9}	4.42 ×10^{-7}	4.30 ×10^{-7}	2.04 ×10^{-7}	6.19 ×10^{-8}	1.40 ×10^{-7}	1.97 ×10^{-8}
Ozone form. hum.	kg NO_x equiv	1.60 ×10^{-7}	1.79 ×10^{-7}	4.07 ×10^{-7}	4.73 ×10^{-7}	1.25 ×10^{-6}	2.73 ×10^{-7}	3.47 ×10^{-7}	9.62 ×10^{-8}	3.62 ×10^{-8}
Particulate matter	kg PM2.5 equiv	7.18 ×10^{-8}	6.75 ×10^{-8}	1.31 ×10^{-7}	2.27 ×10^{-7}	7.14 ×10^{-7}	1.47 ×10^{-7}	1.55 ×10^{-7}	4.21 ×10^{-8}	1.07 ×10^{-8}
Ozone form. ter.	kg NO_x equiv	2.08 ×10^{-7}	2.32 ×10^{-7}	5.10 ×10^{-7}	6.82 ×10^{-7}	1.52 ×10^{-6}	3.35 ×10^{-7}	4.12 ×10^{-7}	1.19 ×10^{-7}	4.26 ×10^{-8}
Ter. acidification	kg SO_2 equiv	1.17 ×10^{-7}	1.29 ×10^{-7}	2.49 ×10^{-7}	5.60 ×10^{-7}	1.71 ×10^{-6}	2.22 ×10^{-7}	2.53 ×10^{-7}	8.47 ×10^{-8}	2.09 ×10^{-8}
Water eutrophication	kg P equiv	3.31 ×10^{-7}	4.40 ×10^{-7}	1.10 ×10^{-7}	1.55 ×10^{-6}	8.11 ×10^{-6}	8.75 ×10^{-7}	6.30 ×10^{-7}	4.45 ×10^{-7}	7.82 ×10^{-9}
Marine eutrophication	kg N equiv	2.49 ×10^{-9}	1.70 ×10^{-9}	1.23 ×10^{-9}	1.33 ×10^{-8}	3.24 ×10^{-6}	9.79 ×10^{-9}	1.12 ×10^{-8}	5.10 ×10^{-7}	2.62 ×10^{-9}
Terrestrial ecotoxicity	kg 1,4-DCB	2.62 ×10^{-9}	5.89 ×10^{-9}	2.01 ×10^{-9}	3.36 ×10^{-7}	5.32 ×10^{-8}	1.85 ×10^{-8}	7.70 ×10^{-9}	4.07 ×10^{-9}	1.03 ×10^{-8}
Water ecotoxicity	kg 1,4-DCB	1.95 ×10^{-8}	2.30 ×10^{-8}	1.21 ×10^{-8}	4.31 ×10^{-6}	4.63 ×10^{-7}	1.77 ×10^{-7}	2.35 ×10^{-7}	4.62 ×10^{-8}	1.54 ×10^{-9}
Marine ecotoxicity	kg 1,4-DCB	1.60 ×10^{-8}	1.75 ×10^{-8}	1.02 ×10^{-8}	6.78 ×10^{-7}	3.89 ×10^{-7}	1.49 ×10^{-7}	1.92 ×10^{-7}	3.54 ×10^{-8}	3.26 ×10^{-9}
Carcinogenic toxicity hum.	kg 1,4-DCB	9.39 ×10^{-8}	1.55 ×10^{-7}	7.31 ×10^{-9}	3.52 ×10^{-8}	1.06 ×10^{-7}	7.37 ×10^{-8}	1.36 ×10^{-7}	2.22 ×10^{-8}	3.77 ×10^{-8}
Non-carcinogenic toxicity hum.	kg 1,4-DCB	8.87 ×10^{-11}	1.58 ×10^{-10}	8.54 ×10^{-11}	4.46 ×10^{-6}	2.65 ×10^{-9}	9.95 ×10^{-10}	1.47 ×10^{-9}	2.98 ×10^{-10}	-9.22 ×10^{-11}
Land use	m^2	6.34 ×10^{-6}	8.94 ×10^{-6}	5.61 ×10^{-6}	4.75 ×10^{-5}	1.76 ×10^{-4}	6.76 ×10^{-5}	2.65 ×10^{-5}	2.06 ×10^{-5}	9.44 ×10^{-7}
Mineral scarcity	kg Cu equiv	5.29 ×10^{-12}	1.29 ×10^{-11}	4.09 ×10^{-12}	5.85 ×10^{-11}	2.39 ×10^{-10}	7.87 ×10^{-11}	2.13 ×10^{-10}	2.75 ×10^{-11}	1.29 ×10^{-12}
Fossil scarcity	kg oil equiv	1.01 ×10^{-6}	1.03 ×10^{-6}	4.56 ×10^{-6}	1.70 ×10^{-6}	8.64 ×10^{-6}	2.46 ×10^{-6}	5.19 ×10^{-7}	8.60 ×10^{-7}	1.91 ×10^{-7}
Water consumption	m^3	2.00 ×10^{-5}	1.87 ×10^{-5}	1.10 ×10^{-4}	5.29 ×10^{-5}	2.13 ×10^{-4}	5.88 ×10^{-4}	2.01 ×10^{-5}	1.17 ×10^{-4}	3.87 ×10^{-3}

Table S38. Component-wise endpoint results in the zolpidem manufacturing process considering batch method.

Damage category	Unit	Keto	Oxal	Tol	A.P.	MeCN	DIP-EA	Me_2 AlCl	NH	Elec.
Human health	DALY	5.79×10^{-10}	3.93×10^{-9}	2.97×10^{-8}	5.90×10^{-9}	9.58×10^{-8}	9.26×10^{-9}	1.46×10^{-8}	3.98×10^{-9}	4.14×10^{-9}
Ecosystems	Species.yr	1.38×10^{-12}	1.00×10^{-11}	8.08×10^{-11}	2.84×10^{-12}	2.56×10^{-10}	2.10×10^{-11}	3.15×10^{-11}	1.09×10^{-11}	1.15×10^{-11}
Resources	USD2013	8.47×10^{-5}	5.91×10^{-4}	7.35×10^{-3}	8.94×10^{-5}	1.08×10^{-2}	1.69×10^{-3}	5.61×10^{-4}	6.02×10^{-4}	2.83×10^{-4}

Table S39. Component-wise endpoint results in the zolpidem manufacturing process considering continuous-flow method.

Damage category	Unit	Keto	Oxal	Tol	A.P.	MeCN	DIP-EA	Me_2 AlCl	NH	Elec.
Human health	DALY	2.61×10^{-9}	2.66×10^{-9}	7.41×10^{-9}	3.89×10^{-8}	2.82×10^{-8}	4.53×10^{-9}	4.29×10^{-9}	1.95×10^{-9}	8.87×10^{-10}
Ecosystems	Species.yr	6.23×10^{-12}	6.80×10^{-12}	2.02×10^{-11}	1.88×10^{-11}	7.54×10^{-11}	1.03×10^{-11}	9.26×10^{-12}	5.31×10^{-12}	2.46×10^{-12}
Resources	USD2013	3.83×10^{-5}	4.01×10^{-4}	1.84×10^{-3}	5.90×10^{-4}	3.17×10^{-3}	8.27×10^{-4}	1.65×10^{-4}	2.94×10^{-4}	6.05×10^{-5}

7. Rufinamide results

Table S40. Component-wise e-factor results in the rufinamide process, comparing traditional batch and continuous-flow methods. NaN_3, DMSO and NH_3 represent sodium azide, dimethyl sulfoxide and ammonia, respectively.

**Compounds**	**Waste batch (mol)**	**Waste flow (mol/h)**	**Flow duration (h)**	**E-factor (batch)**	**E-factor (flow)**
Aryl Bromide	0.000005	0.000000	0.96	0.005	0.000
NaN_3	0.000005	0.000201	0.96	0.002	0.012
DMSO	0.035200	0.031100	0.96	14.007	2.287
NH_3	0.001052	0.000280	0.96	0.091	0.004
Methyl Propiolate	0.000462	0.000000	0.96	0.198	0.000
H_2O	0.016650	0.002720	0.96	1.528	0.046
**Total:**				15.83	2.35

Table S41. Component-wise CO_2 emission results in the rufinamide process, comparing traditional batch and continuous-flow methods. NaN_3, DMSO and NH_3 represent sodium azide, dimethyl sulfoxide and ammonia, respectively.

Compounds	Batch (kg CO_2 equiv)	Flow (kg CO_2 equiv)	Sub-components	Batch (kg CO_2 equiv)	Flow (kg CO_2 equiv)
Aryl bromide	4.19×10^{-3}	2.12×10^{-3}	Benzene	1.07×10^{-3}	5.41×10^{-4}
			F_2	3.08×10^{-4}	1.56×10^{-4}
			Br_2	2.81×10^{-3}	1.43×10^{-3}
NaN_3	3.50×10^{-4}	2.31×10^{-4}	Na	5.86×10^{-5}	3.87×10^{-5}
			N_2	2.91×10^{-4}	1.92×10^{-4}
DMSO	1.96×10^{-2}	1.62×10^{-2}	DMSO	1.96×10^{-2}	1.62×10^{-2}
NH_3	1.03×10^{-3}	5.57×10^{-4}	NH_3	1.03×10^{-3}	5.57×10^{-4}
Methyl propiolate	1.50×10^{-3}	8.50×10^{-4}	Acetylene	5.64×10^{-4}	3.20×10^{-4}
			Methyl ethyl ketone	9.35×10^{-4}	5.31×10^{-4}
Deionized water	1.28×10^{-6}	1.95×10^{-7}	Deionized water	1.28×10^{-6}	1.95×10^{-7}
Electricity	5.54×10^{-2}	7.52×10^{-5}	Electricity	5.54×10^{-2}	7.52×10^{-5}
**Total:**	0.08	0.02			

Table S42. Component-wise water consumption results in the rufinamide process, comparing traditional batch and continuous-flow methods. NaN_3, DMSO and NH_3 represent sodium azide, dimethyl sulfoxide and ammonia, respectively.

**Compounds**	**Batch (m^3)**	**Flow (m^3)**	**Sub-components**	**Batch (m^3)**	**Flow (m^3)**
Aryl bromide	4.20×10^{-5}	5.47×10^{-5}	Benzene	1.55×10^{-5}	2.02×10^{-5}
			F_2	1.43×10^{-5}	1.87×10^{-5}
			Br_2	1.21×10^{-5}	1.58×10^{-5}
NaN_3	7.07×10^{-6}	1.20×10^{-5}	Na	3.35×10^{-6}	5.67×10^{-5}
			N_2	3.72×10^{-6}	6.30×10^{-5}
DMSO	3.64×10^{-4}	7.72×10^{-4}	DMSO	3.64×10^{-4}	7.72×10^{-5}
NH_3	1.16×10^{-5}	1.62×10^{-5}	NH_3	1.16×10^{-5}	1.62×10^{-5}
Methyl propiolate	4.56×10^{-5}	6.63×10^{-5}	Acetylene	3.22×10^{-5}	4.68×10^{-5}
			Methyl ethyl ketone	1.34×10^{-5}	1.94×10^{-5}
Deionized water	6.61×10^{-6}	2.59×10^{-6}	Deionized water	6.61×10^{-6}	2.59×10^{-6}
Electricity	3.09×10^{-1}	1.08×10^{-3}	Electricity	3.09×10^{-1}	1.08×10^{-3}
**Total:**	0.309	0.002			

Table S43. Component-wise land system change results in the rufinamide process, comparing traditional batch and continuous-flow methods. NaN_3, DMSO and NH_3 represent sodium azide, dimethyl sulfoxide and ammonia, respectively.

**Compounds**	**Batch ($\times 10^{-3}$ m^2)**	**Flow ($\times 10^{-3}$ m^2)**	**Sub-components**	**Batch ($\times 10^{-3}$ m^2)**	**Flow ($\times 10^{-3}$ m^2)**
Aryl bromide	5.31×10^{-2}	2.75×10^{-2}	Benzene	4.59×10^{-3}	2.38×10^{-3}
			F_2	1.17×10^{-2}	6.08×10^{-3}
			Br_2	3.67×10^{-2}	1.91×10^{-2}
NaN_3	1.04×10^{-2}	7.01×10^{-3}	Na	2.04×10^{-3}	1.38×10^{-3}
			N_2	8.35×10^{-3}	5.63×10^{-3}
DMSO	4.63×10^{-1}	3.92×10^{-1}	DMSO	4.63×10^{-1}	3.92×10^{-1}
NH_3	9.87×10^{-3}	5.46×10^{-3}	NH_3	9.87×10^{-3}	5.46×10^{-3}
Methyl propiolate	3.14×10^{-2}	1.82×10^{-2}	Acetylene	2.03×10^{-2}	1.17×10^{-2}
			Methyl ethyl ketone	1.11×10^{-2}	6.46×10^{-3}
Deionized water	1.10×10^{-5}	1.72×10^{-6}	Deionized water	1.10×10^{-5}	1.72×10^{-6}
Electricity	7.28×10^{-2}	1.01×10^{-4}	Electricity	7.28×10^{-2}	1.01×10^{-4}
**Total:**	0.64	0.45			

Table S44. Component-wise environmental impact assessment results in the rufinamide manufacturing process considering batch method. NaN_3, DMSO, NH_3 and Elec. represent sodium azide, dimethyl sulfoxide, ammonia and electricity respectively.

Impact category	Unit	Aryl-bromide	NaN_3	DMSO	NH_3	Methyl propiolate	H_2O	Elec.
Global warming	kg CO_2 equiv	4.19×10^{-3}	3.50×10^{-4}	1.96×10^{-2}	1.03×10^{-3}	1.50×10^{-3}	1.28×10^{-6}	5.54×10^{-2}
Ozone depletion	kg CFC11 equiv	1.72×10^{-8}	2.25×10^{-9}	1.42×10^{-7}	1.50×10^{-9}	7.62×10^{-9}	1.41×10^{-11}	2.07×10^{-7}
Ionizing radiation	kBq Co-60 equiv	2.43×10^{-7}	1.22×10^{-7}	2.28×10^{-6}	9.69×10^{-9}	7.25×10^{-7}	1.12×10^{-10}	1.53×10^{-6}
Ozone form. hum.	kg NO_x equiv	3.45×10^{-7}	3.96×10^{-8}	2.14×10^{-6}	6.65×10^{-8}	1.37×10^{-7}	1.26×10^{-10}	2.81×10^{-6}
Particulate matter	kg PM2.5 equiv	2.50×10^{-7}	2.47×10^{-8}	1.75×10^{-6}	4.75×10^{-8}	6.44×10^{-8}	5.83×10^{-11}	8.28×10^{-7}
Ozone form. ter.	kg NO_x equiv	4.19×10^{-7}	4.65×10^{-8}	2.58×10^{-6}	8.13×10^{-8}	1.73×10^{-7}	1.49×10^{-10}	3.30×10^{-6}
Ter. acidification	kg SO_2 equiv	4.17×10^{-7}	3.23×10^{-8}	3.04×10^{-6}	6.34×10^{-8}	1.07×10^{-7}	9.73×10^{-11}	1.62×10^{-6}
Water eutrophication	kg P equiv	1.24×10^{-6}	2.85×10^{-7}	9.72×10^{-6}	2.95×10^{-7}	1.26×10^{-6}	1.18×10^{-10}	6.06×10^{-7}
Marine eutrophication	kg N equiv	1.24×10^{-8}	4.89×10^{-9}	7.69×10^{-8}	2.56×10^{-9}	1.13×10^{-8}	4.72×10^{-12}	2.03×10^{-7}
Terrestrial ecotoxicity	kg 1,4-DCB	3.25×10^{-8}	4.19×10^{-9}	1.64×10^{-7}	3.24×10^{-9}	1.33×10^{-8}	1.79×10^{-10}	8.03×10^{-7}
Water ecotoxicity	kg 1,4-DCB	1.14×10^{-7}	2.59×10^{-8}	1.14×10^{-6}	2.29×10^{-8}	6.53×10^{-8}	4.68×10^{-10}	1.20×10^{-7}
Marine ecotoxicity	kg 1,4-DCB	1.02×10^{-7}	2.20×10^{-8}	9.15×10^{-7}	2.01×10^{-8}	5.56×10^{-8}	4.08×10^{-10}	2.53×10^{-7}
Carcinogenic toxicity hum.	kg 1,4-DCB	9.68×10^{-8}	7.93×10^{-9}	4.14×10^{-7}	1.40×10^{-8}	9.11×10^{-9}	5.24×10^{-10}	2.92×10^{-6}
Non-carcinogenic toxicity hum.	kg 1,4-DCB	7.37×10^{-10}	1.58×10^{-10}	7.38×10^{-9}	1.01×10^{-10}	3.32×10^{-10}	2.10×10^{-11}	7.15×10^{-9}
Land use	m^2	5.31×10^{-5}	1.04×10^{-5}	4.63×10^{-4}	9.87×10^{-6}	3.14×10^{-5}	1.10×10^{-8}	7.28×10^{-5}
Mineral scarcity	kg Cu equiv	5.60×10^{-11}	1.27×10^{-11}	6.19×10^{-10}	8.91×10^{-12}	2.55×10^{-11}	6.45×10^{-14}	1.00×10^{-10}
Fossil scarcity	kg oil equiv	1.75×10^{-6}	9.52×10^{-8}	1.40×10^{-5}	1.71×10^{-7}	8.26×10^{-7}	2.85×10^{-10}	1.48×10^{-5}
Water consumption	m^3	4.20×10^{-5}	7.07×10^{-6}	3.64×10^{-4}	1.16×10^{-5}	4.56×10^{-5}	6.61×10^{-6}	3.09×10^{-1}

Table S45. Component-wise environmental impact assessment results in the rufinamide manufacturing process considering continuous-flow method. NaN_3, DMSO, NH_3 and Elec. represent sodium azide, dimethyl sulfoxide, ammonia and electricity respectively.

Impact category	Unit	Aryl-bromide	NaN_3	DMSO	NH_3	Methyl propiolate	H_2O	Elec.
Global warming	kg CO_2 equiv	2.12×10^{-3}	2.31×10^{-4}	1.62×10^{-2}	5.57×10^{-4}	8.50×10^{-4}	1.95×10^{-7}	7.52×10^{-5}
Ozone depletion	kg CFC11 equiv	8.96×10^{-9}	1.52×10^{-9}	1.21×10^{-7}	8.33×10^{-10}	4.43×10^{-9}	2.20×10^{-12}	2.88×10^{-10}
Ionizing radiation	kBq Co-60 equiv	1.26×10^{-7}	8.23×10^{-8}	1.93×10^{-6}	5.37×10^{-9}	4.21×10^{-7}	1.75×10^{-11}	2.13×10^{-9}
Ozone form. hum.	kg NO_x equiv	1.79×10^{-7}	2.68×10^{-8}	1.81×10^{-6}	3.69×10^{-8}	7.98×10^{-8}	1.97×10^{-11}	3.91×10^{-9}
Particulate matter	kg PM2.5 equiv	1.30×10^{-7}	1.67×10^{-8}	1.48×10^{-6}	2.63×10^{-8}	3.74×10^{-8}	9.12×10^{-12}	1.15×10^{-9}
Ozone form. ter.	kg NO_x equiv	2.18×10^{-7}	3.14×10^{-8}	2.19×10^{-6}	4.51×10^{-8}	1.00×10^{-7}	2.33×10^{-11}	4.60×10^{-9}
Ter. acidification	kg SO_2 equiv	2.17×10^{-7}	2.18×10^{-8}	2.58×10^{-6}	3.51×10^{-8}	6.23×10^{-8}	1.52×10^{-11}	2.25×10^{-9}
Water eutrophication	kg P equiv	6.43×10^{-7}	1.93×10^{-7}	8.25×10^{-6}	1.63×10^{-7}	7.32×10^{-7}	1.85×10^{-11}	8.44×10^{-10}
Marine eutrophication	kg N equiv	6.47×10^{-9}	3.31×10^{-9}	6.52×10^{-8}	1.42×10^{-9}	6.55×10^{-9}	7.39×10^{-13}	2.83×10^{-10}
Terrestrial ecotoxicity	kg 1,4-DCB	1.69×10^{-8}	2.84×10^{-9}	1.39×10^{-7}	1.80×10^{-9}	7.74×10^{-9}	2.80×10^{-11}	1.12×10^{-9}
Water ecotoxicity	kg 1,4-DCB	5.94×10^{-8}	1.75×10^{-8}	9.67×10^{-7}	1.27×10^{-8}	3.80×10^{-8}	7.33×10^{-11}	1.67×10^{-10}
Marine ecotoxicity	kg 1,4-DCB	5.29×10^{-8}	1.49×10^{-8}	7.76×10^{-7}	1.12×10^{-8}	3.23×10^{-8}	6.38×10^{-11}	3.52×10^{-10}
Carcinogenic toxicity hum.	kg 1,4-DCB	5.03×10^{-8}	5.36×10^{-9}	3.51×10^{-7}	7.75×10^{-9}	5.29×10^{-9}	8.20×10^{-11}	4.07×10^{-9}
Non-carcinogenic toxicity hum.	kg 1,4-DCB	3.83×10^{-10}	1.07×10^{-10}	6.26×10^{-9}	5.63×10^{-11}	1.93×10^{-10}	3.28×10^{-12}	-9.96×10^{-12}
Land use	m^2	2.75×10^{-5}	7.01×10^{-6}	3.92×10^{-4}	5.46×10^{-6}	1.82×10^{-5}	1.72×10^{-9}	1.01×10^{-7}
Mineral scarcity	kg Cu equiv	2.91×10^{-11}	8.59×10^{-12}	5.25×10^{-10}	4.94×10^{-12}	1.48×10^{-11}	1.01×10^{-14}	1.40×10^{-13}
Fossil scarcity	kg oil equiv	9.12×10^{-7}	6.44×10^{-8}	1.19×10^{-5}	9.46×10^{-8}	4.80×10^{-7}	4.45×10^{-11}	2.07×10^{-8}
Water consumption	m^3	5.47×10^{-5}	1.20×10^{-5}	7.72×10^{-4}	1.62×10^{-5}	6.63×10^{-5}	2.59×10^{-6}	1.08×10^{-3}

Table S46. Component-wise endpoint results in the rufinamide manufacturing process considering batch method.

Damage category	Unit	Aryl-bromide	NaN_3	DMSO	NH_3	Methyl propiolate	H_2O	Elec.
Human health	DALY	7.98×10^{-9}	7.34×10^{-10}	4.70×10^{-8}	1.73×10^{-9}	2.51×10^{-9}	3.69×10^{-12}	6.88×10^{-8}
Ecosystems	Species.yr	1.78×10^{-11}	1.67×10^{-12}	1.00×10^{-10}	3.91×10^{-12}	6.86×10^{-12}	9.10×10^{-15}	1.91×10^{-10}
Resources	USD2013	6.04×10^{-4}	2.36×10^{-5}	4.73×10^{-3}	2.48×10^{-5}	2.94×10^{-4}	8.24×10^{-8}	4.70×10^{-3}

Table S47. Component-wise endpoint results in the rufinamide manufacturing process considering continuous-flow method.

Damage category	Unit	Aryl-bromide	NaN_3	DMSO	NH_3	Methyl propiolate	H_2O	Elec.
Human health	DALY	4.15×10^{-9}	4.97×10^{-10}	3.98×10^{-8}	9.57×10^{-10}	1.46×10^{-9}	5.77×10^{-13}	9.58×10^{-11}
Ecosystems	Species.yr	9.28×10^{-12}	1.13×10^{-12}	8.50×10^{-11}	2.17×10^{-12}	3.99×10^{-12}	1.42×10^{-15}	2.66×10^{-13}
Resources	USD2013	3.14×10^{-4}	1.59×10^{-5}	4.01×10^{-3}	1.37×10^{-5}	1.71×10^{-4}	1.29×10^{-8}	6.54×10^{-6}

8. Artesunate results

Table S48. Component-wise e-factor results in the artesunate process, comparing traditional batch and continuous-flow methods. EtOH, $NaBH_4$, Li_2CO_3, LiCl, DCM, and HCl represent ethanol, sodium borohydride, lithium carbonate, lithium chloride, dichloromethane and hydrochloric acid, respectively.

Compounds	Waste batch (mol)	Waste flow (mol/h)	Flow duration (h)	E-factor (batch)	E-factor (flow)
Artemisinin	0.000	0.000	0.16	0.36	0.00
EtOH	0.242	0.200	0.16	35.96	0.85
$NaBH_4$	0.007	0.032	0.16	0.84	0.11
Li_2CO_3	0.003	0.014	0.16	0.78	0.10
LiCl	0.005	0.020	0.16	0.62	0.08
DCM	0.185	0.172	0.16	50.68	1.35
Succinic anhydride	0.002	0.012	0.16	0.73	0.11
HCl	0.000	0.000	0.16	0.03	0.00
**Total:**				90.01	2.61

Table S49. Component-wise CO_2 emission results in the artesunate process, comparing traditional batch and continuous-flow methods. EtOH, $NaBH_4$, Li_2CO_3, LiCl, DCM, and HCl represent ethanol, sodium borohydride, lithium carbonate, lithium chloride, dichloromethane and hydrochloric acid, respectively.

**Compounds**	**Batch (kg CO_2 equiv)**	**Flow (kg CO_2 equiv)**	**Sub-components**	**Batch (kg CO_2 equiv)**	**Flow (kg CO_2 equiv)**
Artemisinin	3.40×10^{-3}	2.56×10^{-3}	THF	2.41×10^{-3}	1.81×10^{-3}
			Cyclohexane	5.92×10^{-4}	4.46×10^{-4}
			Methyl ethyl ketone	3.50×10^{-4}	2.63×10^{-4}
			O_2	5.44×10^{-5}	4.10×10^{-5}
EtOH	4.42×10^{-2}	5.49×10^{-3}	EtOH	4.42×10^{-2}	5.49×10^{-3}
$NaBH_4$	1.09×10^{-4}	8.09×10^{-5}	$NaBH_4$	1.09×10^{-4}	8.09×10^{-5}
Li_2CO_3	7.06×10^{-3}	4.56×10^{-3}	Li_2CO_3	7.06×10^{-3}	4.56×10^{-3}
LiCl	6.30×10^{-3}	4.30×10^{-3}	LiCl	6.30×10^{-3}	4.30×10^{-3}
DCM	1.83×10^{-1}	2.55×10^{-2}	DCM	1.83×10^{-1}	2.55×10^{-2}
Succinic anyhdride	3.69×10^{-3}	2.94×10^{-3}	THF	2.26×10^{-3}	2.94×10^{-3}
			Acetone	1.43×10^{-3}	1.14×10^{-3}
HCl	8.46×10^{-5}	7.73×10^{-5}	HCl	8.46×10^{-5}	7.73×10^{-5}
Electricity	9.27×10^{-4}	5.20×10^{-4}	Electricity	9.27×10^{-4}	5.20×10^{-4}
**Total:**	0.25	0.05			

Table S50. Component-wise water consumption results in the artesunate process, comparing traditional batch and continuous-flow methods. EtOH, $NaBH_4$, Li_2CO_3, LiCl, DCM, and HCl represent ethanol, sodium borohydride, lithium carbonate, lithium chloride, dichloromethane and hydrochloric acid, respectively.

Compounds	Batch (m^3)	Flow (m^3)	Sub-components	Batch (m^3)	Flow (m^3)
Artemisinin	4.18×10^{-4}	3.32×10^{-4}	THF	1.26×10^{-4}	1.00×10^{-4}
			Cyclohexane	8.07×10^{-6}	6.42×10^{-6}
			Methyl ethyl ketone	4.90×10^{-6}	3.90×10^{-6}
			O_2	2.79×10^{-4}	2.22×10^{-4}
EtOH	3.82×10^{-4}	5.01×10^{-5}	EtOH	3.82×10^{-4}	5.01×10^{-5}
$NaBH_4$	2.48×10^{-7}	1.94×10^{-7}	$NaBH_4$	2.48×10^{-7}	1.94×10^{-7}
Li_2CO_3	9.50×10^{-5}	6.49×10^{-5}	Li_2CO_3	9.50×10^{-5}	6.49×10^{-5}
LiCl	8.95×10^{-5}	6.46×10^{-5}	LiCl	8.95×10^{-5}	6.46×10^{-5}
DCM	5.15×10^{-4}	7.59×10^{-5}	DCM	5.15×10^{-4}	7.59×10^{-5}
Succinic anyhdride	1.36×10^{-4}	1.14×10^{-4}	THF	1.18×10^{-4}	9.99×10^{-5}
			Acetone	1.72×10^{-5}	1.45×10^{-5}
HCl	3.03×10^{-4}	2.92×10^{-4}	HCl	3.03×10^{-4}	2.92×10^{-4}
Electricity	5.07×10^{-3}	3.01×10^{-3}	Electricity	5.07×10^{-3}	3.01×10^{-3}
**Total:**	0.007	0.004			

Table S51. Component-wise land system change results in the artesunate process, comparing traditional batch and continuous-flow methods. EtOH, $NaBH_4$, Li_2CO_3, LiCl, DCM, and HCl represent ethanol, sodium borohydride, lithium carbonate, lithium chloride, dichloromethane and hydrochloric acid, respectively.

**Compounds**	**Batch ($\times10^{-3}$ m^2)**	**Flow ($\times10^{-3}$ m^2)**	**Sub-components**	**Batch ($\times10^{-3}$ m^2)**	**Flow ($\times10^{-3}$ m^2)**
Artemisinin	7.25×10^{-2}	5.49×10^{-2}	THF	6.21×10^{-2}	4.70×10^{-2}
			Cyclohexane	6.16×10^{-3}	4.66×10^{-3}
			Methyl ethyl ketone	4.20×10^{-3}	3.18×10^{-3}
			O_2	8.21×10^{-5}	6.22×10^{-5}
EtOH	5.65×10^{-1}	7.05×10^{-2}	EtOH	5.65×10^{-1}	7.05×10^{-2}
$NaBH_4$	9.92×10^{-3}	7.39×10^{-3}	$NaBH_4$	9.92×10^{-3}	7.39×10^{-2}
Li_2CO_3	2.19×10^{-1}	1.42×10^{-1}	Li_2CO_3	2.19×10^{-1}	1.42×10^{-1}
LiCl	1.85×10^{-1}	1.27×10^{-1}	LiCl	1.85×10^{-1}	1.27×10^{-1}
DCM	3.36×10^{-2}	4.70×10^{-3}	DCM	3.36×10^{-2}	4.70×10^{-3}
Succinic anyhdride	6.39×10^{-2}	5.13×10^{-2}	THF	5.83×10^{-2}	4.67×10^{-2}
			Acetone	5.64×10^{-3}	4.52×10^{-3}
HCl	1.41×10^{-3}	1.29×10^{-3}	HCl	1.41×10^{-3}	1.29×10^{-3}
Electricity	1.23×10^{-3}	6.94×10^{-4}	Electricity	1.23×10^{-3}	6.94×10^{-4}
**Total:**	1.15	0.46			

Table S52. Component-wise environmental impact assessment results in the artesunate manufacturing process considering batch method. Art, EtOH, $NaBH_4$, Li_2CO_3, LiCl, DCM, S. A. and HCl represent artemisinin, ethanol, sodium borohydride, lithium carbonate, lithium chloride, dichloromethane, succinic anhydride and hydrochloric acid, respectively.

Impact category	Unit	Art	Et-OH	$NaBH_4$	Li_2CO_3	Li Cl	DCM	S. A.	HCl	Elec.
Global warming	kg CO_2 equiv	3.40×10^{-3}	4.42×10^{-2}	1.09×10^{-4}	7.06×10^{-3}	6.30×10^{-3}	1.83×10^{-1}	3.69×10^{-3}	8.46×10^{-5}	9.27×10^{-4}
Ozone depletion	kg CFC11 equiv	1.69×10^{-8}	6.89×10^{-8}	1.16×10^{-9}	4.70×10^{-8}	4.35×10^{-8}	5.36×10^{-5}	1.48×10^{-8}	1.12×10^{-9}	3.48×10^{-9}
Ionizing radiation	kBq Co-60 equiv	4.49×10^{-7}	1.51×10^{-6}	9.60×10^{-9}	5.45×10^{-7}	5.88×10^{-7}	5.99×10^{-8}	4.17×10^{-7}	6.37×10^{-9}	2.58×10^{-8}
Ozone form. hum.	kg NO_x equiv	3.62×10^{-7}	4.01×10^{-6}	5.94×10^{-8}	1.21×10^{-6}	9.77×10^{-7}	3.49×10^{-5}	4.10×10^{-7}	8.70×10^{-9}	4.72×10^{-8}
Particulate matter	kg PM2.5 equiv	2.03×10^{-7}	1.51×10^{-6}	2.01×10^{-8}	6.60×10^{-7}	5.57×10^{-7}	1.29×10^{-5}	2.09×10^{-7}	5.18×10^{-9}	1.39×10^{-8}
Ozone form. ter.	kg NO_x equiv	4.41×10^{-7}	4.97×10^{-6}	6.97×10^{-8}	1.41×10^{-6}	1.15×10^{-6}	4.10×10^{-5}	5.09×10^{-7}	1.02×10^{-8}	5.56×10^{-8}
Ter. acidification	kg SO_2 equiv	2.83×10^{-7}	2.72×10^{-6}	2.34×10^{-8}	1.07×10^{-6}	8.72×10^{-7}	2.21×10^{-5}	3.18×10^{-7}	9.32×10^{-9}	2.72×10^{-8}
Water eutrophication	kg P equiv	1.62×10^{-6}	2.63×10^{-5}	3.12×10^{-8}	1.20×10^{-5}	1.04×10^{-5}	8.20×10^{-6}	1.51×10^{-6}	2.85×10^{-8}	1.02×10^{-8}
Marine eutrophication	kg N equiv	1.53×10^{-8}	6.15×10^{-8}	2.90×10^{-10}	1.20×10^{-6}	8.68×10^{-7}	2.23×10^{-8}	1.33×10^{-8}	4.25×10^{-10}	3.42×10^{-9}
Terrestrial ecotoxicity	kg 1,4-DCB	4.57×10^{-8}	5.62×10^{-7}	1.49×10^{-9}	1.04×10^{-7}	8.55×10^{-8}	1.22×10^{-8}	3.91×10^{-8}	2.93×10^{-8}	1.35×10^{-8}
Water ecotoxicity	kg 1,4-DCB	1.51×10^{-7}	1.04×10^{-6}	6.35×10^{-9}	4.27×10^{-7}	3.86×10^{-7}	8.11×10^{-7}	1.37×10^{-7}	1.31×10^{-7}	2.01×10^{-9}
Marine ecotoxicity	kg 1,4-DCB	1.32×10^{-7}	9.84×10^{-7}	5.56×10^{-9}	3.68×10^{-7}	3.31×10^{-7}	8.62×10^{-7}	1.17×10^{-7}	1.11×10^{-7}	4.25×10^{-9}
Carcinogenic toxicity hum.	kg 1,4-DCB	3.13×10^{-7}	2.80×10^{-7}	1.10×10^{-9}	1.15×10^{-7}	1.05×10^{-7}	3.77×10^{-5}	3.80×10^{-7}	1.03×10^{-7}	4.92×10^{-8}
Non-carcinogenic toxicity hum.	kg 1,4-DCB	8.88×10^{-10}	9.48×10^{-9}	2.40×10^{-11}	4.17×10^{-9}	3.63×10^{-9}	2.64×10^{-7}	7.55×10^{-10}	5.35×10^{-9}	-1.20×10^{-10}
Land use	m^2	7.25×10^{-5}	5.65×10^{-4}	9.92×10^{-6}	2.19×10^{-4}	1.85×10^{-4}	3.36×10^{-5}	6.39×10^{-5}	1.41×10^{-6}	1.23×10^{-6}
Mineral scarcity	kg Cu equiv	6.15×10^{-11}	8.44×10^{-10}	1.73×10^{-12}	3.80×10^{-9}	2.76×10^{-9}	2.02×10^{-10}	5.44×10^{-11}	4.16×10^{-12}	1.69×10^{-12}
Fossil scarcity	kg oil equiv	1.52×10^{-6}	3.51×10^{-5}	3.23×10^{-8}	1.90×10^{-6}	1.59×10^{-6}	3.44×10^{-5}	1.69×10^{-6}	2.13×10^{-8}	2.50×10^{-7}
Water consumption	m^3	4.18×10^{-4}	3.82×10^{-4}	2.48×10^{-7}	9.50×10^{-5}	8.95×10^{-5}	5.15×10^{-4}	1.36×10^{-4}	3.03×10^{-4}	5.07×10^{-3}

Table S53. Component-wise environmental impact assessment results in the artesunate manufacturing process considering continuous-flow method. Art, EtOH, $NaBH_4$, Li_2CO_3, LiCl, DCM, S.A. and HCl represent artemisinin, ethanol, sodium borohydride, lithium carbonate, lithium chloride, dichloromethane, succinic anhydride and hydrochloric acid, respectively.

**Impact category**	**Unit**	**Art**	**Et-OH**	**$NaBH_4$**	**Li_2CO_3**	**LiCl**	**DCM**	**S.A.**	**HCl**	**Elec.**
Global warming	kg CO_2 equiv	2.56×10^{-3}	5.49×10^{-3}	8.09×10^{-5}	4.56×10^{-3}	4.30×10^{-3}	2.55×10^{-2}	2.94×10^{-3}	7.73×10^{-5}	5.20×10^{-4}
Ozone depletion	kg CFC11 equiv	1.27×10^{-8}	8.59×10^{-9}	8.66×10^{-10}	3.05×10^{-8}	2.98×10^{-8}	7.51×10^{-6}	1.18×10^{-8}	1.03×10^{-9}	1.96×10^{-9}
Ionizing radiation	kBq Co-60 equiv	3.39×10^{-7}	1.88×10^{-7}	7.15×10^{-9}	3.53×10^{-7}	4.03×10^{-7}	8.38×10^{-9}	3.34×10^{-7}	5.84×10^{-9}	1.45×10^{-8}
Ozone form. hum.	kg NO_x equiv	2.73×10^{-7}	5.00×10^{-7}	4.42×10^{-8}	7.82×10^{-7}	6.70×10^{-7}	4.89×10^{-6}	3.28×10^{-7}	7.98×10^{-9}	2.66×10^{-8}
Particulate matter	kg PM2.5 equiv	1.53×10^{-7}	1.89×10^{-7}	1.50×10^{-8}	4.28×10^{-7}	3.82×10^{-7}	1.80×10^{-6}	1.67×10^{-7}	4.75×10^{-9}	7.85×10^{-9}
Ozone form. ter.	kg NO_x equiv	3.33×10^{-7}	6.20×10^{-7}	5.19×10^{-8}	9.17×10^{-7}	7.85×10^{-7}	5.74×10^{-6}	4.08×10^{-7}	9.35×10^{-9}	3.13×10^{-8}
Ter. acidification	kg SO_2 equiv	2.14×10^{-7}	3.39×10^{-7}	1.74×10^{-8}	6.96×10^{-7}	5.98×10^{-7}	3.10×10^{-6}	2.55×10^{-7}	8.54×10^{-9}	1.53×10^{-8}
Water eutrophication	kg P equiv	1.22×10^{-6}	3.27×10^{-6}	2.32×10^{-8}	7.79×10^{-6}	7.12×10^{-6}	1.15×10^{-6}	1.21×10^{-6}	2.62×10^{-8}	5.75×10^{-9}
Marine eutrophication	kg N equiv	1.15×10^{-8}	7.67×10^{-9}	2.16×10^{-10}	7.81×10^{-7}	5.95×10^{-7}	3.13×10^{-9}	1.06×10^{-8}	3.90×10^{-10}	1.93×10^{-9}
Terrestrial ecotoxicity	kg 1,4-DCB	3.45×10^{-8}	7.00×10^{-8}	1.11×10^{-9}	6.73×10^{-8}	5.86×10^{-8}	1.70×10^{-9}	3.14×10^{-8}	2.69×10^{-8}	7.61×10^{-9}
Water ecotoxicity	kg 1,4-DCB	1.14×10^{-7}	1.29×10^{-7}	4.72×10^{-9}	2.77×10^{-7}	2.65×10^{-7}	1.14×10^{-7}	1.10×10^{-7}	1.20×10^{-7}	1.13×10^{-9}
Marine ecotoxicity	kg 1,4-DCB	9.97×10^{-8}	1.23×10^{-7}	4.14×10^{-9}	2.39×10^{-7}	2.27×10^{-7}	1.21×10^{-7}	9.34×10^{-8}	1.01×10^{-7}	2.40×10^{-9}
Carcinogenic toxicity hum.	kg 1,4-DCB	2.37×10^{-7}	3.49×10^{-8}	8.20×10^{-10}	7.44×10^{-8}	7.19×10^{-8}	5.28×10^{-6}	3.04×10^{-7}	9.42×10^{-8}	2.77×10^{-8}
Non-carcinogenic toxicity hum.	kg 1,4-DCB	6.71×10^{-10}	1.18×10^{-9}	1.79×10^{-11}	2.70×10^{-9}	2.49×10^{-9}	3.70×10^{-8}	6.05×10^{-10}	4.91×10^{-9}	-6.78×10^{-11}
Land use	m^2	5.49×10^{-5}	7.05×10^{-5}	7.39×10^{-6}	1.42×10^{-4}	1.27×10^{-4}	4.70×10^{-6}	5.13×10^{-5}	1.29×10^{-6}	6.94×10^{-7}
Mineral scarcity	kg Cu equiv	4.64×10^{-11}	1.05×10^{-10}	1.29×10^{-12}	2.46×10^{-9}	1.89×10^{-9}	2.83×10^{-11}	4.35×10^{-11}	3.81×10^{-12}	9.50×10^{-13}
Fossil scarcity	kg oil equiv	1.15×10^{-6}	4.38×10^{-6}	2.40×10^{-8}	1.23×10^{-6}	1.09×10^{-6}	4.82×10^{-6}	1.35×10^{-6}	1.95×10^{-8}	1.41×10^{-7}
Water consumption	m^3	3.32×10^{-4}	5.01×10^{-5}	1.94×10^{-7}	6.49×10^{-5}	6.46×10^{-5}	7.59×10^{-5}	1.14×10^{-4}	2.92×10^{-4}	3.01×10^{-3}

Table S54. Component-wise endpoint results in the artesunate manufacturing process considering batch method.

Damage category	Unit	Art	Et-OH	$NaBH_4$	Li_2CO_3	LiCl	DCM	S. A.	HCl	Elec.
Human health	DALY	6.74×10^{-9}	6.71×10^{-8}	4.26×10^{-10}	1.74×10^{-8}	1.50×10^{-8}	3.83×10^{-7}	7.10×10^{-9}	2.04×10^{-10}	1.16×10^{-9}
Ecosystems	Species.yr	1.63×10^{-11}	1.86×10^{-10}	7.73×10^{-13}	4.13×10^{-11}	3.56×10^{-11}	8.09×10^{-10}	1.73×10^{-11}	3.86×10^{-13}	3.21×10^{-12}
Resources	USD2013	4.89×10^{-4}	1.41×10^{-2}	1.23×10^{-5}	6.16×10^{-4}	4.95×10^{-4}	1.08×10^{-2}	5.63×10^{-4}	6.91×10^{-6}	7.90×10^{-5}

Table S55. Component-wise endpoint results in the artesunate manufacturing process considering continuous-flow method.

Damage category	Unit	Art	Et-OH	$NaBH_4$	Li_2CO_3	LiCl	DCM	S. A.	HCl	Elec.
Human health	DALY	5.09×10^{-9}	8.37×10^{-9}	3.17×10^{-10}	1.13×10^{-8}	1.03×10^{-8}	5.37×10^{-8}	5.69×10^{-9}	1.87×10^{-10}	6.52×10^{-10}
Ecosystems	Species.yr	1.23×10^{-11}	2.32×10^{-11}	5.76×10^{-13}	2.68×10^{-11}	2.44×10^{-11}	1.13×10^{-10}	1.39×10^{-11}	3.54×10^{-13}	1.81×10^{-12}
Resources	USD2013	3.70×10^{-4}	1.76×10^{-3}	9.14×10^{-6}	3.99×10^{-4}	3.39×10^{-4}	1.51×10^{-3}	3.51×10^{-4}	6.33×10^{-6}	4.45×10^{-5}

9. Ibuprofen results

Table S56. Component-wise E-factor results in the ibuprofen process, comparing traditional batch and continuous-flow methods. $AlCl_3$, HCl, TMOF, DMF, ICl, NaOH, MeOH, and 2-ME represent aluminum chloride, hydrochloric acid, trimethyl orthoformate, dimethylformamide, iodine monochloride, sodium hydroxide, methanol and 2-mercaptoethanol, respectively.

Compounds	Waste batch (mol)	Waste flow (mol/h)	Flow duration (h)	E-factor (batch)	E-factor (flow)
Isobutyl benzene	0.0054	0.0017	0.05	0.081	0.008
Propionyl chloride	0.0121	0.0091	0.05	0.125	0.030
$AlCl_3$	0.0121	0.0065	0.05	0.180	0.031
HCl	0.0006	0.0300	0.05	0.002	0.040
TMOF	2.9848	0.2649	0.05	49.558	1.421
DMF	0.1370	0.0106	0.05	1.122	0.028
ICl	0.0228	0.0002	0.05	0.414	0.001
Isopropanol	0.0000	0.0023	0.05	0.000	0.005
NaOH(2M)	0.4268	1.0899	0.05	1.913	1.578
MeOH	13.4130	3.0277	0.05	48.147	3.511
H_2O	1.1060	1.9257	0.05	2.230	1.254
2-ME	0.9210	0.0284	0.05	8.062	0.080
**Total:**				111.83	7.99

Table S57. Component-wise CO_2 emission results in the ibuprofen process, comparing traditional batch and continuous-flow methods. $AlCl_3$, HCl, TMOF, DMF, ICl, NaOH, MeOH, and 2-ME represent aluminum chloride, hydrochloric acid, trimethyl orthoformate, dimethylformamide, iodine monochloride, sodium hydroxide, methanol and 2-mercaptoethanol, respectively.

Compounds	Batch (kg CO_2 equiv)	Flow (kg CO_2 equiv)	Sub-components	Batch (kg CO_2 equiv)	Flow (kg CO_2 equiv)
Isobutyl benzene	3.57×10^{-4}	3.41×10^{-4}	Butane	8.67×10^{-5}	8.27×10^{-5}
			Benzene	2.70×10^{-4}	2.58×10^{-4}
Propionyl chloride	2.55×10^{-4}	2.60×10^{-4}	Methyl ethyl ketone	2.09×10^{-4}	2.12×10^{-4}
			Cl_2	4.66×10^{-5}	4.74×10^{-5}
$AlCl_3$	1.38×10^{-3}	1.32×10^{-3}	$AlCl_3$	1.38×10^{-3}	1.32×10^{-4}
TMOF	2.77×10^{-2}	5.02×10^{-3}	TMOF	2.77×10^{-2}	9.50×10^{-5}
DMF	7.00×10^{-4}	9.50×10^{-5}	DMF	7.00×10^{-4}	3.72×10^{-5}
ICl	1.33×10^{-3}	3.88×10^{-5}	ICl	1.28×10^{-3}	1.61×10^{-6}
NaOH	5.58×10^{-4}	2.16×10^{-3}	NaOH	5.45×10^{-5}	2.16×10^{-3}
MeOH	6.87×10^{-3}	2.55×10^{-3}	MeOH	5.58×10^{-4}	2.55×10^{-3}
Deionized water	4.27×10^{-7}	1.21×10^{-6}	Deionized water	6.87×10^{-3}	1.21×10^{-6}
2-ME	1.31×10^{-3}	2.22×10^{-4}	Ethanol	4.27×10^{-7}	2.06×10^{-4}
			S_2	1.22×10^{-3}	1.57×10^{-5}
Electricity	3.70×10^{-1}	9.38×10^{-4}	Electricity	9.31×10^{-5}	9.38×10^{-4}
**Total:**	0.41	0.01			

Table S58. Component-wise water consumption results in the ibuprofen process, comparing traditional batch and continuous-flow methods. $AlCl_3$, HCl, TMOF, DMF, ICl, NaOH, MeOH, and 2-ME represent aluminum chloride, hydrochloric acid, trimethyl orthoformate, dimethylformamide, iodine monochloride, sodium hydroxide, methanol and 2-mercaptoethanol, respectively.

Compounds	Batch (m^3)	Flow (m^3)	Sub-components	Batch (m^3)	Flow (m^3)
Isobutyl benzene	4.28×10^{-6}	7.94×10^{-6}	Butane	3.28×10^{-7}	6.10×10^{-7}
			Benzene	3.95×10^{-6}	7.33×10^{-6}
Propionyl chloride	3.25×10^{-6}	6.44×10^{-6}	Methyl ethyl ketone	2.99×10^{-6}	5.92×10^{-6}
			Cl_2	2.60×10^{-7}	5.14×10^{-7}
$AlCl_3$	1.24×10^{-5}	2.09×10^{-4}	$AlCl_3$	1.24×10^{-5}	2.09×10^{-4}
TMOF	1.43×10^{-3}	5.05×10^{-4}	TMOF	1.43×10^{-3}	5.05×10^{-4}
DMF	8.86×10^{-6}	2.34×10^{-6}	DMF	8.86×10^{-6}	2.34×10^{-6}
ICl	5.60×10^{-6}	3.18×10^{-7}	ICl	5.60×10^{-6}	3.18×10^{-7}
NaOH	1.64×10^{-6}	1.23×10^{-6}	NaOH	1.64×10^{-6}	1.23×10^{-6}
MeOH	4.16×10^{-5}	3.00×10^{-5}	MeOH	4.16×10^{-5}	3.00×10^{-5}
Deionized water	1.29×10^{-6}	7.10×10^{-6}	Deionized water	1.29×10^{-6}	7.10×10^{-6}
2-ME	1.10×10^{-5}	3.63×10^{-6}	Ethanol	1.08×10^{-5}	3.55×10^{-6}
			S_2	2.55×10^{-7}	8.40×10^{-8}
Electricity	2.07×10^{0}	1.02×10^{-2}	Electricity	2.07×10^{0}	1.02×10^{-2}
**Total:**	2.070	0.110			

Table S59. Component-wise land system change results in the ibuprofen process, comparing traditional batch and continuous-flow methods. $AlCl_3$, HCl, TMOF, DMF, ICl, NaOH, MeOH, and 2-ME represent aluminum chloride, hydrochloric acid, trimethyl orthoformate, dimethylformamide, iodine monochloride, sodium hydroxide, methanol and 2-mercaptoethanol, respectively.

**Compounds**	**Batch ($\times 10^{-3}$ m^2)**	**Flow ($\times 10^{-3}$ m^2)**	**Sub-components**	**Batch ($\times 10^{-3}$ m^2)**	**Flow ($\times 10^{-3}$ m^2)**
Isobutyl benzene	3.83×10^{-3}	3.43×10^{-3}	Butane	2.65×10^{-3}	2.38×10^{-3}
			Benzene	1.17×10^{-3}	1.05×10^{-3}
Propionyl chloride	3.59×10^{-3}	3.43×10^{-3}	Methyl ethyl ketone	2.51×10^{-3}	2.40×10^{-3}
			Cl_2	1.08×10^{-3}	1.03×10^{-3}
$AlCl_3$	2.12×10^{-2}	1.91×10^{-2}	$AlCl_3$	2.12×10^{-2}	1.91×10^{-2}
TMOF	7.85×10^{-1}	1.34×10^{-1}	TMOF	7.85×10^{-1}	1.34×10^{-1}
DMF	1.73×10^{-1}	2.20×10^{-3}	DMF	1.73×10^{-1}	2.20×10^{-3}
ICl	1.83×10^{-3}	5.00×10^{-4}	ICl	1.83×10^{-2}	5.00×10^{-4}
NaOH	0	0	NaOH	0	0
MeOH	1.24×10^{-1}	4.31×10^{-2}	MeOH	1.24×10^{-1}	4.31×10^{-2}
Deionized water	-2.11×10^{-5}	-5.59×10^{-5}	Deionized water	-2.11×10^{-5}	-5.59×10^{-5}
2-ME	1.74×10^{-2}	2.76×10^{-3}	Ethanol	1.56×10^{-2}	2.48×10^{-3}
			S_2	1.75×10^{-3}	2.78×10^{-4}
Electricity	4.91×10^{-1}	1.17×10^{-3}	Electricity	4.91×10^{-1}	1.17×10^{-3}
**Total:**	1.48	0.21			

Table S60. Component-wise environmental impact assessment results in the ibuprofen process, considering batch method. I.P, P.C, $AlCl_3$, HCl, TF, DF, ICl, NaOH, MeOH, and 2-ME represent isobutyl benzene, propionyl chloride, aluminum chloride, hydrochloric acid, trimethyl orthoformate, dimethylformamide, iodine monochloride, sodium hydroxide, methanol and 2-mercaptoethanol, respectively.

Impact category	Unit	I.P	P.C	Al-Cl_3	HCl	TF	DF	ICl	NaOH	MeOH	H_2O	2-ME	Elec.
Global warming	kg CO_2 equiv	3.57×10^{-4}	2.55×10^{-4}	1.38×10^{-3}	2.77×10^{-2}	7.00×10^{-4}	1.33×10^{-3}	5.58×10^{-4}	6.87×10^{-3}	4.27×10^{-7}	1.31×10^{-3}	3.70×10^{-1}	3.57×10^{-4}
Ozone depletion	kg CFC11 equiv	9.85×10^{-10}	8.11×10^{-10}	8.50×10^{-9}	2.28×10^{-7}	4.54×10^{-9}	6.84×10^{-9}	2.81×10^{-9}	6.11×10^{-8}	2.26×10^{-12}	2.49×10^{-9}	1.39×10^{-6}	9.85×10^{-10}
Ionizing radiation	kBq Co-60 equiv	8.63×10^{-9}	2.66×10^{-8}	1.05×10^{-7}	5.28×10^{-6}	8.83×10^{-8}	7.99×10^{-8}	0	2.27×10^{-7}	1.72×10^{-11}	4.37×10^{-8}	1.03×10^{-5}	8.63×10^{-9}
Ozone form. hum.	kg NO_x equiv	3.74×10^{-8}	2.46×10^{-8}	1.72×10^{-7}	3.21×10^{-6}	6.77×10^{-8}	9.88×10^{-8}	5.46×10^{-8}	6.69×10^{-7}	4.09×10^{-11}	1.22×10^{-7}	1.88×10^{-5}	3.74×10^{-8}
Particulate matter	kg PM2.5 equiv	1.95×10^{-8}	1.03×10^{-8}	1.09×10^{-7}	1.84×10^{-6}	3.77×10^{-8}	5.74×10^{-8}	4.82×10^{-8}	2.53×10^{-7}	1.65×10^{-11}	1.14×10^{-7}	5.55×10^{-6}	1.95×10^{-8}
Ozone form. ter.	kg NO_x equiv	4.75×10^{-8}	3.12×10^{-8}	2.00×10^{-7}	3.90×10^{-6}	8.16×10^{-8}	1.17×10^{-7}	6.40×10^{-8}	8.27×10^{-7}	5.21×10^{-11}	2.78×10^{-8}	2.21×10^{-5}	4.75×10^{-8}
Ter. acidification	kg SO_2 equiv	2.80×10^{-8}	1.69×10^{-8}	1.46×10^{-7}	2.44×10^{-6}	5.67×10^{-8}	9.02×10^{-8}	1.03×10^{-7}	4.36×10^{-7}	2.63×10^{-11}	1.55×10^{-7}	1.08×10^{-5}	2.80×10^{-8}
Water eutrophication	kg P equiv	9.68×10^{-8}	1.16×10^{-7}	7.89×10^{-7}	1.62×10^{-5}	3.86×10^{-7}	3.57×10^{-7}	3.82×10^{-9}	1.22×10^{-7}	4.25×10^{-11}	7.38×10^{-7}	4.06×10^{-6}	9.68×10^{-8}
Marine eutrophication	kg N equiv	9.69×10^{-10}	6.72×10^{-10}	8.13×10^{-9}	1.51×10^{-7}	1.52×10^{-7}	3.95×10^{-9}	3.14×10^{-10}	1.37×10^{-8}	2.92×10^{-11}	1.86×10^{-9}	1.36×10^{-6}	9.69×10^{-10}
Terrestrial ecotoxicity	kg 1,4-DCB	1.13×10^{-9}	2.20×10^{-9}	9.59×10^{-9}	3.45×10^{-7}	2.34×10^{-8}	1.23×10^{-8}	2.63×10^{-10}	1.42×10^{-8}	6.61×10^{-11}	2.05×10^{-8}	5.38×10^{-6}	1.13×10^{-9}
Water ecotoxicity	kg 1,4-DCB	1.06×10^{-8}	9.89×10^{-8}	4.55×10^{-7}	1.65×10^{-6}	6.56×10^{-8}	1.44×10^{-7}	1.42×10^{-10}	2.71×10^{-7}	3.27×10^{-10}	3.12×10^{-8}	8.02×10^{-7}	1.06×10^{-8}
Marine ecotoxicity	kg 1,4-DCB	9.14×10^{-9}	8.15×10^{-8}	3.76×10^{-7}	1.39×10^{-6}	2.99×10^{-8}	1.21×10^{-7}	1.41×10^{-10}	1.93×10^{-7}	2.89×10^{-11}	6.06×10^{-9}	1.69×10^{-6}	9.14×10^{-9}
Carcinogenic toxicity hum.	kg 1,4-DCB	7.21×10^{-9}	1.82×10^{-9}	6.01×10^{-8}	4.39×10^{-7}	1.06×10^{-8}	3.36×10^{-8}	6.45×10^{-9}	1.75×10^{-7}	4.59×10^{-8}	2.76×10^{-9}	1.96×10^{-5}	7.21×10^{-9}

Non-carcinogenic toxicity hum.	kg 1,4-DCB	4.90 $\times 10^{-11}$	2.97 $\times 10^{-10}$	1.36 $\times 10^{-9}$	8.40 $\times 10^{-9}$	2.06 $\times 10^{-10}$	4.94 $\times 10$-^{10}	1.49 $\times 10^{-11}$	1.17 $\times 10^{-9}$	5.59 $\times 10^{-12}$	2.80 $\times 10^{-10}$	4.79 $\times 10^{-8}$	4.90 $\times 10^{-11}$
Land use	m^2	3.83 $\times 10^{-6}$	3.59 $\times 10^{-6}$	2.12 $\times 10^{-5}$	7.85 $\times 10^{-4}$	1.73 $\times 10^{-4}$	1.83 $\times 10^{-5}$	0	1.24 $\times 10^{-4}$	2.11 $\times 10^{-8}$	1.74 $\times 10^{-5}$	4.91 $\times 10^{-4}$	3.83 $\times 10^{-6}$
Mineral scarcity	kg Cu equiv	3.52 $\times 10^{-12}$	1.43 $\times 10^{-11}$	1.16 $\times 10^{-10}$	5.51 $\times 10^{-10}$	1.69 $\times 10^{-11}$	1.42 $\times 10^{-8}$	0	8.60 $\times 10^{-11}$	5.06 $\times 10^{-14}$	2.41 $\times 10^{-11}$	6.72 $\times 10^{-10}$	3.52 $\times 10^{-12}$
Fossil scarcity	kg oil equiv	2.92 $\times 10^{-7}$	1.60 $\times 10^{-7}$	3.12 $\times 10^{-7}$	1.71 $\times 10^{-5}$	3.75 $\times 10^{-7}$	4.07 $\times 10^{-7}$	1.89 $\times 10^{-7}$	7.64 $\times 10^{-6}$	8.00 $\times 10^{-11}$	1.09 $\times 10^{-6}$	9.94 $\times 10^{-5}$	2.92 $\times 10^{-7}$
Water consumption	m^3	4.28 $\times 10^{-6}$	3.25 $\times 10^{-6}$	1.24 $\times 10^{-5}$	1.43 $\times 10^{-3}$	8.86 $\times 10^{-6}$	5.60 $\times 10^{-6}$	1.64 $\times 10^{-6}$	4.16 $\times 10^{-5}$	1.29 $\times 10^{-6}$	1.10 $\times 10^{-5}$	2.07 $\times 10^{0}$	4.28 $\times 10^{-6}$

Table S61. Component-wise environmental impact assessment results in the ibuprofen manufacturing process considering continuous-flow method.

Impact category	Unit	I.P	P.C	Al-Cl$_3$	HCl	TF	DF	ICl	NaOH	MeOH	H_2O	2-ME	Elec.
Global warming	kg CO_2 equiv	3.41 $\times10^{-4}$	2.60 $\times10^{-4}$	1.32 $\times10^{-3}$	2.94 $\times10^{-5}$	5.02 $\times10^{-3}$	9.50 $\times10^{-5}$	3.88 $\times10^{-5}$	2.16 $\times10^{-3}$	2.55 $\times10^{-3}$	1.21 $\times10^{-6}$	2.22 $\times10^{-4}$	9.38 $\times10^{-4}$
Ozone depletion	kg CFC11 equiv	9.04 $\times10^{-10}$	7.93 $\times10^{-10}$	7.82 $\times10^{-9}$	3.74 $\times10^{-10}$	3.98 $\times10^{-8}$	5.93 $\times10^{-10}$	1.91 $\times10^{-10}$	1.05 $\times10^{-8}$	2.18 $\times10^{-8}$	-6.15 $\times10^{-12}$	4.03 $\times10^{-10}$	3.38 $\times10^{-9}$
Ionizing radiation	kBq Co-60 equiv	7.91 $\times10^{-9}$	2.60 $\times10^{-8}$	9.64 $\times10^{-8}$	2.12 $\times10^{-9}$	9.22 $\times10^{-7}$	1.15 $\times10^{-8}$	2.24 $\times10^{-9}$	0	8.11 $\times10^{-8}$	4.67 $\times10^{-11}$	7.10 $\times10^{-9}$	2.50 $\times10^{-8}$
Ozone form. hum.	kg NO_x equiv	3.43 $\times10^{-8}$	2.40 $\times10^{-8}$	1.58 $\times10^{-7}$	2.90 $\times10^{-9}$	5.60 $\times10^{-7}$	8.83 $\times10^{-9}$	2.77 $\times10^{-9}$	2.03 $\times10^{-7}$	2.39 $\times10^{-7}$	1.11 $\times10^{-10}$	1.98 $\times10^{-8}$	4.59 $\times10^{-8}$
Particulate matter	kg PM2.5 equiv	1.79 $\times10^{-8}$	1.00 $\times10^{-8}$	1.01 $\times10^{-7}$	1.73 $\times10^{-9}$	3.22 $\times10^{-7}$	4.91 $\times10^{-9}$	1.61 $\times10^{-9}$	1.79 $\times10^{-7}$	9.03 $\times10^{-8}$	4.47 $\times10^{-11}$	1.86 $\times10^{-8}$	1.35 $\times10^{-8}$
Ozone form. ter.	kg NO_x equiv	4.36 $\times10^{-8}$	3.05 $\times10^{-8}$	1.84 $\times10^{-7}$	3.40 $\times10^{-9}$	6.80 $\times10^{-7}$	1.06 $\times10^{-8}$	3.27 $\times10^{-9}$	2.38 $\times10^{-7}$	2.95 $\times10^{-7}$	1.41 $\times10^{-10}$	2.46 $\times10^{-8}$	5.40 $\times10^{-8}$
Ter. acidification	kg SO_2 equiv	2.57 $\times10^{-8}$	1.65 $\times10^{-8}$	1.34 $\times10^{-7}$	3.11 $\times10^{-9}$	4.25 $\times10^{-7}$	7.39 $\times10^{-9}$	2.52 $\times10^{-9}$	3.84 $\times10^{-7}$	1.56 $\times10^{-7}$	7.16 $\times10^{-11}$	3.73 $\times10^{-8}$	2.65 $\times10^{-8}$
Water eutrophication	kg P equiv	8.87 $\times10^{-8}$	1.14 $\times10^{-7}$	7.26 $\times10^{-7}$	9.51 $\times10^{-9}$	2.83 $\times10^{-6}$	5.03 $\times10^{-8}$	1.10 $\times10^{-8}$	1.42 $\times10^{-8}$	4.36 $\times10^{-7}$	1.15 $\times10^{-10}$	1.20 $\times10^{-7}$	9.91 $\times10^{-9}$
Marine eutrophication	kg N equiv	8.88 $\times10^{-10}$	6.57 $\times10^{-10}$	7.48 $\times10^{-9}$	1.42 $\times10^{-10}$	2.63 $\times10^{-8}$	1.99 $\times10^{-8}$	1.11 $\times10^{-10}$	1.17 $\times10^{-9}$	4.88 $\times10^{-9}$	7.93 $\times10^{-11}$	3.02 $\times10^{-10}$	3.32 $\times10^{-9}$
Terrestrial ecotoxicity	kg 1,4-DCB	1.03 $\times10^{-9}$	2.15 $\times10^{-9}$	8.83 $\times10^{-9}$	9.78 $\times10^{-9}$	6.02 $\times10^{-8}$	3.05 $\times10^{-9}$	3.45 $\times10^{-10}$	9.77 $\times10^{-10}$	5.07 $\times10^{-9}$	1.80 $\times10^{-10}$	3.32 $\times10^{-9}$	1.31 $\times10^{-8}$
Water ecotoxicity	kg 1,4-DCB	9.68 $\times10^{-9}$	9.67 $\times10^{-8}$	4.18 $\times10^{-7}$	4.37 $\times10^{-8}$	2.88 $\times10^{-7}$	8.56 $\times10^{-9}$	4.06 $\times10^{-9}$	5.28 $\times10^{-10}$	9.67 $\times10^{-8}$	8.90 $\times10^{-10}$	5.06 $\times10^{-9}$	1.96 $\times10^{-9}$
Marine ecotoxicity	kg 1,4-DCB	8.38 $\times10^{-9}$	7.97 $\times10^{-8}$	3.45 $\times10^{-7}$	3.69 $\times10^{-8}$	2.42 $\times10^{-7}$	3.90 $\times10^{-9}$	3.41 $\times10^{-9}$	5.23 $\times10^{-10}$	6.89 $\times10^{-8}$	7.85 $\times10^{-10}$	4.96 $\times10^{-9}$	4.13 $\times10^{-9}$
Carcinogenic toxicity hum.	kg 1,4-DCB	6.61 $\times10^{-9}$	1.78 $\times10^{-9}$	5.53 $\times10^{-8}$	3.43 $\times10^{-8}$	7.67 $\times10^{-8}$	1.38 $\times10^{-9}$	9.41 $\times10^{-10}$	2.39 $\times10^{-8}$	6.25 $\times10^{-8}$	1.25 $\times10^{-7}$	1.58 $\times10^{-9}$	4.78 $\times10^{-8}$
Non-carcinogenic toxicity hum.	kg 1,4-DCB	4.50 $\times10^{-11}$	2.90 $\times10^{-10}$	1.25 $\times10^{-9}$	1.78 $\times10^{-9}$	1.47 $\times10^{-9}$	2.69 $\times10^{-11}$	1.39 $\times10^{-11}$	5.53 $\times10^{-11}$	4.16 $\times10^{-10}$	1.52 $\times10^{-11}$	4.54 $\times10^{-11}$	-1.17 $\times10^{-10}$

Land use	m^2	3.43×10^{-6}	3.43×10^{-6}	1.91×10^{-5}	4.60×10^{-7}	1.34×10^{-4}	2.20×10^{-6}	5.00×10^{-7}	0	4.31×10^{-5}	-5.59×10^{-8}	2.76×10^{-6}	1.17×10^{-6}
Mineral scarcity	kg Cu equiv	3.23×10^{-12}	1.40×10^{-11}	1.07×10^{-10}	1.39×10^{-12}	9.62×10^{-11}	2.20×10^{-12}	3.75×10^{-6}	0	3.07×10^{-11}	1.38×10^{-6}	1.28×10^{-6}	1.64×10^{-12}
Fossil scarcity	kg oil equiv	2.68×10^{-7}	1.57×10^{-7}	2.87×10^{-7}	7.09×10^{-9}	2.99×10^{-6}	4.89×10^{-8}	1.14×10^{-8}	7.04×10^{-7}	2.72×10^{-6}	2.17×10^{-10}	1.78×10^{-7}	2.43×10^{-7}
Water consumption	m^3	7.94×10^{-6}	6.44×10^{-6}	2.31×10^{-5}	2.09×10^{-4}	5.05×10^{-4}	2.34×10^{-6}	3.18×10^{-7}	1.23×10^{-5}	3.00×10^{-5}	7.10×10^{-6}	3.63×10^{-6}	1.02×10^{-2}

Table S62. Component-wise endpoint results in the ibuprofen manufacturing process considering batch method.

Damage category	Unit	I.P	P.C	Al-Cl_3	HCl	TF	DF	ICl	NaOH	MeOH	H_2O	2-ME	Elec.
Human health	DALY	6.55×10^{-10}	4.11×10^{-10}	3.07×10^{-9}	7.81×10^{-10}	5.84×10^{-9}	1.27×10^{-9}	2.17×10^{-9}	1.30×10^{-9}	1.05×10^{-8}	2.68×10^{-12}	3.11×10^{-9}	4.61×10^{-7}
Ecosystems	Species.yr	1.49×10^{-12}	1.06×10^{-12}	6.30×10^{-12}	3.44×10^{-11}	1.41×10^{-10}	3.08×10^{-12}	5.19×10^{-12}	2.63×10^{-12}	2.72×10^{-11}	2.64×10^{-15}	6.79×10^{-12}	1.28×10^{-9}
Resources	USD2013	1.06×10^{-4}	6.12×10^{-5}	6.21×10^{-5}	2.90×10^{-4}	5.65×10^{-3}	1.24×10^{-4}	5.22×10^{-4}	4.95×10^{-5}	2.65×10^{-3}	2.05×10^{-8}	4.34×10^{-4}	3.15×10^{-2}

Table S63. Component-wise endpoint results in the ibuprofen manufacturing process considering batch method.

Damage category	Unit	I.P	P.C	Al-Cl_3	HCl	TF	DF	ICl	NaOH	MeOH	H_2O	2-ME	Elec.
Human health	DALY	6.01×10^{-10}	4.01×10^{-10}	2.82×10^{-9}	6.78×10^{-11}	1.02×10^{-8}	1.66×10^{-10}	6.08×10^{-11}	4.82×10^{-9}	3.76×10^{-9}	7.27×10^{-12}	5.04×10^{-10}	1.12×10^{-9}
Ecosystems	Species.yr	1.36×10^{-12}	1.04×10^{-12}	5.80×10^{-12}	1.29×10^{-13}	2.45×10^{-11}	4.01×10^{-13}	1.45×10^{-13}	9.76×10^{-12}	9.69×10^{-12}	7.17×10^{-15}	1.10×10^{-12}	3.12×10^{-12}
Resources	USD2013	9.68×10^{-5}	5.98×10^{-5}	5.71×10^{-5}	2.30×10^{-6}	9.85×10^{-4}	1.62×10^{-5}	1.46×10^{-5}	1.84×10^{-4}	9.45×10^{-4}	5.57×10^{-8}	7.05×10^{-5}	7.68×10^{-5}

10. Phenibut results

Table S64. Component-wise E-factor results in the phenibut process, comparing traditional batch and continuous-flow methods. TsCl, TEMPO, BF_3OEt_2 and TBAF represent 4-toluenesulfonyl chloride, 2,2,6,6-tetramethylpiperidine 1-oxyl, boron trifluoride etherate and tetra-n-butylammonium fluoride, respectively.

Compounds	Waste batch (mol)	Waste flow (mol/h)	Flow duration (h)	E-factor (batch)	E-factor (flow)
Benzaldehyde	0.0030830	0.0000000	0.05	0.3142949	0.0000000
Nitromethane	0.2012900	0.0272261	0.05	11.8032968	0.1573939
NaOAc	0.0013760	0.0000000	0.05	0.1084321	0.0000000
KBr	0.0000000	0.0000001	0.05	0.0000000	0.0000008
NaOCl	0.0000000	0.0000009	0.05	0.0000000	0.0000062
MeOH	0.6179775	0.0000000	0.05	19.0209320	0.0000000
TsCl	0.0227700	0.0000000	0.05	4.1702918	0.0000000
Et_3N	0.0390000	0.0000000	0.05	3.7911311	0.0000000
THF	0.2462904	0.0069806	0.05	17.0611996	0.0476730
TEMPO	0.0000000	0.0000000	0.05	0.0000000	0.0000000
Thiourea	0.0000555	0.0000000	0.05	0.0040584	0.0000000
Diethyl malonate	0.0050000	0.0000000	0.05	0.7693391	0.0000000
Toluene	0.2634686	0.4286473	0.05	23.3208154	3.7405522
$NaBH_4$	0.0350000	0.0000000	0.05	1.2719528	0.0000000
BF_3oEt_2	0.0017000	0.0000000	0.05	0.2317873	0.0000000
Benzyl alcohol	0.0000000	0.0000000	0.05	0.0000000	0.0000000
TBAF	0.0000000	0.0000530	0.05	0.0000000	0.0013128
HCl	0.0025000	0.0000000	0.05	0.0875635	0.0000000
H_2O	0.0018000	0.9762457	0.05	0.0311252	1.6642520
$NaHCO_3$	0.0000000	0.0000001	0.05	0.0000000	0.0000008
Methyl cyanoacetate	0.0000000	0.0001158	0.05	0.0000000	0.0010853
LiOH	0.0000000	0.0016950	0.05	0.0000000	0.0038447
H_2	0.0000000	0.0000276	0.05	0.0000000	0.0000053
**Total:**				81.99	5.62

Table S65. Component-wise CO_2 emission results in the phenibut process, comparing traditional batch and continuous-flow methods.

**Compounds**	**Batch (kg CO_2 equiv)**	**Flow (kg CO_2 equiv)**	**Sub-components**	**Batch (kg CO_2 equiv)**	**Flow (kg CO_2 equiv)**
Benzaldehyde	9.10×10^{-3}	1.64×10^{-3}	Benzaldehyde	9.10×10^{-3}	1.64×10^{-3}
Nitromethane	3.08×10^{-2}	1.85×10^{-3}	Nitrous oxide	3.08×10^{-2}	1.75×10^{-3}
			CH_4	2.06×10^{-3}	9.64×10^{-5}
Toluene	3.86×10^{-2}	9.34×10^{-5}	Toluene	3.86×10^{-2}	9.34×10^{-5}
THF	1.05×10^{-1}	6.23×10^{-4}	THF	1.05×10^{-1}	6.23×10^{-4}
NaOAc	9.13×10^{-4}	0	NaOAc	9.13×10^{-4}	0
NaOCl	0	4.80×10^{-4}	NaOCl	0	4.80×10^{-4}
TsCl	6.21×10^{-3}	0	Sulfuryl chloride	3.41×10^{-3}	0
			Toluene	2.81×10^{-3}	0
KBr	0	9.34×10^{-5}	K	0	5.44×10^{-5}
			Br_2	0	3.90×10^{-5}
Et_3N	1.54×10^{-2}	0	Et_3N	1.54×10^{-2}	0
TBAF	0	3.33×10^{-5}	TBAF	0	3.33×10^{-5}
Thiourea	5.12×10^{-3}	0	Thiourea	5.12×10^{-3}	0
TEMPO	0	3.95×10^{-5}	Piperidine	0	3.87×10^{-5}
			CH_4	0	8.43×10^{-7}
MeOH	1.33×10^{-2}	0	MeOH	1.33×10^{-2}	0
Benzyl alcohol	0	5.10×10^{-4}	Benzyl alcohol	0	5.10×10^{-4}
Diethyl malonate	5.14×10^{-3}	0	Diethyl malonate	5.14×10^{-3}	0
Methyl cyanoacetate	0	3.82×10^{-4}	Methyl cyanoacetate	0	3.82×10^{-4}
Deionized water	2.79×10^{-8}	3.17×10^{-6}	Deionized water	2.79×10^{-8}	3.17×10^{-6}
BF_3oEt_2	8.64×10^{-4}	0	Ethane	1.16×10^{-4}	0
			BF_3	7.48×10^{-4}	0
LiOH	0	3.97×10^{-6}	LiOH	0	3.97×10^{-6}
HCl	6.35×10^{-5}	0	HCl	6.35×10^{-5}	0
H_2	0	9.42×10^{-6}	H_2	0	9.42×10^{-6}
Electricity	2.03×10^{-1}	4.07×10^{-3}	Electricity	2.03×10^{-1}	4.07×10^{-3}
**Total:**	0.43	0.02			

Table S66. Component-wise water consumption results in the phenibut process, comparing traditional batch and continuous-flow methods.

Compounds	Batch (m^3)	Flow (m^3)	Sub-components	Batch (m^3)	Flow (m^3)
Benzaldehyde	2.52×10^{-4}	4.39×10^{-5}	Benzaldehyde	2.52×10^{-4}	4.39×10^{-5}
Nitromethane	1.88×10^{-3}	8.82×10^{-5}	Nitrous oxide	7.74×10^{-4}	3.81×10^{-5}
			CH_4	1.11×10^{-3}	5.01×10^{-5}
Toluene	8.43×10^{-4}	3.29×10^{-3}	Toluene	8.43×10^{-4}	3.29×10^{-3}
THF	5.91×10^{-3}	3.38×10^{-5}	THF	5.91×10^{-3}	4.71×10^{-7}
NaOAc	1.93×10^{-5}	0	NaOAc	1.93×10^{-5}	0
NaOCl	0	1.46×10^{-5}	NaOCl	0	1.46×10^{-5}
TsCl	1.42×10^{-4}	0	Sulfuryl chloride	8.12×10^{-5}	0
			Toluene	6.13×10^{-5}	0
KBr	0	7.04×10^{-7}	K	0	5.33×10^{-7}
			Br_2	0	1.71×10^{-7}
Et_3N	1.65×10^{-4}	0	Et_3N	1.65×10^{-4}	0
TBAF	0	4.03×10^{-5}	TBAF	0	4.03×10^{-5}
Thiourea	1.57×10^{-6}	0	Thiourea	1.57×10^{-6}	0
TEMPO	0	9.09×10^{-7}	Piperidine	0	4.71×10^{-7}
			CH_4	0	4.38×10^{-7}
MeOH	8.40×10^{-5}	0	MeOH	8.40×10^{-5}	0
Benzyl alcohol	0	4.60×10^{-4}	Benzyl alcohol	0	4.60×10^{-4}
Diethyl malonate	1.17×10^{-4}	0	Diethyl malonate	1.17×10^{-4}	0
Methyl cyanoacetate	0	2.18×10^{-5}	Methyl cyanoacetate	0	2.18×10^{-5}
Deionized water	8.80×10^{-8}	9.67×10^{-6}	Deionized water	8.80×10^{-8}	9.67×10^{-6}
BF_3oEt_2	2.19×10^{-5}	0	Ethane	4.10×10^{-7}	0
			BF_3	2.15×10^{-5}	0
LiOH	0	9.75×10^{-9}	LiOH	0	9.75×10^{-9}
HCl	2.43×10^{-4}	0	HCl	2.43×10^{-4}	0
H_2	0	4.64×10^{-9}	H_2	0	4.64×10^{-9}
Electricity	1.19×10^{0}	2.30×10^{-2}	Electricity	1.19×10^{0}	2.30×10^{-2}
**Total:**	1.200	0.270			

Table S67. Component-wise land system change results in the phenibut process, comparing traditional batch and continuous-flow methods.

**Compounds**	**Batch ($\times 10^{-3}$ m^2)**	**Flow ($\times 10^{-3}$ m^2)**	**Sub-components**	**Batch ($\times 10^{-3}$ m^2)**	**Flow ($\times 10^{-3}$ m^2)**
Benzaldehyde	2.17×10^{-1}	3.91×10^{-2}	Benzaldehyde	2.17×10^{-1}	3.91×10^{-2}
Nitromethane	6.12×10^{-1}	3.19×10^{-2}	Nitrous oxide	6.08×10^{-1}	3.17×10^{-2}
			CH_4	3.85×10^{-3}	1.80×10^{-4}
Toluene	3.97×10^{-2}	2.32×10^{-3}	Toluene	3.97×10^{-2}	8.48×10^{-3}
THF	2.71×10^{0}	1.60×10^{-2}	THF	2.71×10^{0}	1.60×10^{-2}
NaOAc	2.97×10^{-2}	0	NaOAc	2.97×10^{-2}	0
NaOCl	0	1.60×10^{-2}	NaOCl	0	1.60×10^{-2}
TsCl	1.05×10^{-1}	0	Sulfuryl chloride	1.02×10^{-1}	0
			Toluene	3.97×10^{-2}	0
KBr	0	2.32×10^{-3}	K	0	1.81×10^{-3}
			Br_2	0	5.13×10^{-4}
Et_3N	2.25×10^{-1}	0	Et_3N	6.12×10^{-1}	0
TBAF	0	8.58×10^{-4}	TBAF	0	8.58×10^{-4}
Thiourea	7.47×10^{-4}	0	Thiourea	7.47×10^{-4}	0
TEMPO	0	5.68×10^{-4}	Piperidine	0	5.66×10^{-4}
			CH_4	0	1.58×10^{-6}
MeOH	2.38×10^{-1}	0	MeOH	2.38×10^{-1}	0
Benzyl alcohol	0	6.52×10^{-3}	Benzyl alcohol	0	6.52×10^{-3}
Diethyl malonate	1.21×10^{-1}	0	Diethyl malonate	1.21×10^{-1}	0
Methyl cyanoacetate	0	1.92×10^{-2}	Methyl cyanoacetate	0	1.92×10^{-2}
Deionized water	-1.37×10^{-6}	-1.56×10^{-4}	Deionized water	-1.37×10^{-6}	-1.56×10^{-4}
BF_3oEt_2	3.81×10^{-2}	0	Ethane	3.56×10^{-3}	0
			BF_3	3.46×10^{-2}	0
LiOH	0	1.21×10^{-3}	LiOH	0	1.21×10^{-3}
HCl	1.05×10^{-3}	0	HCl	1.05×10^{-3}	0
H_2	0	0	H_2	0	0
Electricity	2.69×10^{-1}	5.38×10^{-3}	Electricity	2.69×10^{-1}	5.38×10^{-3}
**Total:**	4.68	0.15			

Table S68. Component-wise environmental impact assessment results in the phenibut manufacturing process considering batch method. NaOAc, TsCl, THF, and Thio. represent sodium acetate, sodium hypochlorite, 4-toluenesulfonyl chloride, 1-oxyl, tetrahydrofuran and thiourea, respectively.

Impact category	Unit	Benzal-dehyde	Nitro methane	NaO Ac	MeOH	TsCl	Et_3N	THF	Thio.
Global warming	kg CO_2 equiv	9.10 $\times10^{-3}$	3.08 $\times10^{-2}$	9.13 $\times10^{-4}$	1.33 $\times10^{-2}$	6.21 $\times10^{-3}$	1.54 $\times10^{-2}$	1.05 $\times10^{-1}$	5.12 $\times10^{-5}$
Ozone depletion	kg CFC11 equiv	7.19 $\times10^{-8}$	1.40 $\times10^{-7}$	7.63 $\times10^{-9}$	1.18 $\times10^{-7}$	3.93 $\times10^{-8}$	5.44 $\times10^{-8}$	6.36 $\times10^{-7}$	1.25 $\times10^{-10}$
Ionizing radiation	kBq Co-60 equiv	2.30 $\times10^{-6}$	6.38 $\times10^{-6}$	1.44 $\times10^{-7}$	4.40 $\times10^{-7}$	8.30 $\times10^{-7}$	6.89 $\times10^{-7}$	1.70 $\times10^{-5}$	9.81 $\times10^{-10}$
Ozone form. hum.	kg NO_x equiv	8.11 $\times10^{-7}$	3.14 $\times10^{-6}$	1.02 $\times10^{-7}$	1.29 $\times10^{-6}$	6.15 $\times10^{-7}$	1.59 $\times10^{-6}$	1.16 $\times10^{-5}$	4.62 $\times10^{-9}$
Particulate matter	kg PM2.5 equiv	3.85 $\times10^{-7}$	1.68 $\times10^{-6}$	6.33 $\times10^{-8}$	4.89 $\times10^{-7}$	1.74 $\times10^{-6}$	6.77 $\times10^{-7}$	6.91 $\times10^{-6}$	3.58 $\times10^{-9}$
Ozone form. ter.	kg NO_x equiv	9.97 $\times10^{-7}$	3.70 $\times10^{-6}$	1.22 $\times10^{-7}$	1.60 $\times10^{-6}$	7.39 $\times10^{-7}$	1.95 $\times10^{-6}$	1.39 $\times10^{-5}$	5.59 $\times10^{-9}$
Ter. acidification	kg SO_2 equiv	6.40 $\times10^{-7}$	2.51 $\times10^{-6}$	8.97 $\times10^{-8}$	8.43 $\times10^{-7}$	3.45 $\times10^{-6}$	1.20 $\times10^{-6}$	9.37 $\times10^{-6}$	5.65 $\times10^{-9}$
Water eutrophication	kg P equiv	5.44 $\times10^{-6}$	1.65 $\times10^{-5}$	5.46 $\times10^{-7}$	2.36 $\times10^{-6}$	2.80 $\times10^{-6}$	1.23 $\times10^{-5}$	5.68 $\times10^{-5}$	1.89 $\times10^{-8}$
Marine eutrophication	kg N equiv	5.63 $\times10^{-8}$	1.80 $\times10^{-6}$	5.66 $\times10^{-9}$	2.64 $\times10^{-8}$	3.18 $\times10^{-8}$	8.86 $\times10^{-7}$	5.54 $\times10^{-7}$	3.07 $\times10^{-10}$
Terrestrial ecotoxicity	kg 1,4-DCB	1.78 $\times10^{-7}$	2.25 $\times10^{-7}$	1.88 $\times10^{-8}$	2.75 $\times10^{-8}$	4.59 $\times10^{-8}$	8.57 $\times10^{-8}$	1.71 $\times10^{-6}$	2.82 $\times10^{-10}$
Water ecotoxicity	kg 1,4-DCB	4.42 $\times10^{-7}$	1.45 $\times10^{-6}$	2.83 $\times10^{-7}$	5.24 $\times10^{-7}$	3.04 $\times10^{-7}$	9.45 $\times10^{-7}$	5.57 $\times10^{-6}$	1.76 $\times10^{-9}$
Marine ecotoxicity	kg 1,4-DCB	3.58 $\times10^{-7}$	1.24 $\times10^{-6}$	5.45 $\times10^{-8}$	3.73 $\times10^{-7}$	2.56 $\times10^{-7}$	7.76 $\times10^{-7}$	4.88 $\times10^{-6}$	1.54 $\times10^{-9}$
Carcinogenic toxicity hum.	kg 1,4-DCB	1.63 $\times10^{-7}$	3.75 $\times10^{-7}$	1.87 $\times10^{-8}$	3.39 $\times10^{-7}$	8.82 $\times10^{-8}$	1.52 $\times10^{-7}$	1.02 $\times10^{-5}$	1.06 $\times10^{-9}$
Non-carcinogenic toxicity hum.	kg 1,4-DCB	3.22 $\times10^{-9}$	8.31 $\times10^{-9}$	4.17 $\times10^{-10}$	2.25 $\times10^{-9}$	2.03 $\times10^{-9}$	4.48 $\times10^{-9}$	3.04 $\times10^{-8}$	9.80 $\times10^{-12}$
Land use	m^2	2.17 $\times10^{-4}$	6.12 $\times10^{-4}$	2.97 $\times10^{-5}$	2.38 $\times10^{-4}$	1.05 $\times10^{-4}$	2.25 $\times10^{-4}$	2.71 $\times10^{-3}$	7.47 $\times10^{-7}$
Mineral scarcity	kg Cu equiv	2.33 $\times10^{-10}$	6.19 $\times10^{-10}$	3.33 $\times10^{-11}$	1.66 $\times10^{-10}$	2.43 $\times10^{-10}$	3.59 $\times10^{-10}$	2.16 $\times10^{-9}$	8.69 $\times10^{-13}$
Fossil scarcity	kg oil equiv	4.46 $\times10^{-6}$	1.19 $\times10^{-5}$	3.02 $\times10^{-7}$	1.48 $\times10^{-5}$	3.44 $\times10^{-6}$	7.73 $\times10^{-6}$	3.89 $\times10^{-5}$	1.27 $\times10^{-8}$
Water consumption	m^3	2.52 $\times10^{-4}$	1.88 $\times10^{-3}$	1.93 $\times10^{-5}$	8.40 $\times10^{-5}$	1.42 $\times10^{-4}$	1.65 $\times10^{-4}$	5.91 $\times10^{-3}$	1.57 $\times10^{-6}$

Table S69. Component-wise environmental impact assessment results in the phenibut manufacturing process considering batch method. $NaBH_4$, BF_3OEt_2, and Elec. represent sodium borohydride, boron trifluoride etherate and electricity, respectively.

Impact category	Unit	Diethyl malonate	$NaBH_4$	BF_3OEt_2	HCl	H_2O	Toluene	Elec.
Global warming	kg CO_2 equiv	5.14×10^{-3}	4.26×10^{-3}	8.64×10^{-4}	6.35×10^{-5}	2.79×10^{-8}	3.86×10^{-2}	2.03×10^{-1}
Ozone depletion	kg CFC11 equiv	4.66×10^{-8}	3.53×10^{-8}	6.88×10^{-9}	8.42×10^{-10}	-1.48×10^{-13}	5.58×10^{-9}	7.62×10^{-7}
Ionizing radiation	kBq Co-60 equiv	7.24×10^{-7}	2.99×10^{-7}	1.13×10^{-7}	4.77×10^{-9}	1.12×10^{-12}	4.95×10^{-8}	5.64×10^{-6}
Ozone form. hum.	kg NO_x equiv	5.30×10^{-7}	1.40×10^{-6}	1.79×10^{-7}	6.53×10^{-9}	2.67×10^{-12}	2.89×10^{-6}	1.03×10^{-5}
Particulate matter	kg PM2.5 equiv	3.10×10^{-7}	6.57×10^{-7}	1.55×10^{-7}	3.88×10^{-9}	1.07×10^{-12}	9.35×10^{-7}	3.05×10^{-6}
Ozone form. ter.	kg NO_x equiv	6.37×10^{-7}	1.67×10^{-6}	2.11×10^{-7}	7.65×10^{-9}	3.40×10^{-12}	3.63×10^{-6}	1.22×10^{-5}
Ter. acidification	kg SO_2 equiv	4.28×10^{-7}	1.19×10^{-6}	2.65×10^{-7}	6.99×10^{-9}	1.72×10^{-12}	1.77×10^{-6}	5.96×10^{-6}
Water eutrophication	kg P equiv	2.89×10^{-6}	1.02×10^{-6}	7.71×10^{-7}	2.14×10^{-8}	2.77×10^{-12}	7.83×10^{-7}	2.23×10^{-6}
Marine eutrophication	kg N equiv	6.05×10^{-6}	1.99×10^{-8}	5.35×10^{-9}	3.19×10^{-10}	1.90×10^{-12}	8.78×10^{-9}	7.49×10^{-7}
Terrestrial ecotoxicity	kg 1,4-DCB	7.87×10^{-7}	2.25×10^{-7}	1.39×10^{-8}	2.20×10^{-8}	4.31×10^{-12}	1.43×10^{-8}	2.96×10^{-6}
Water ecotoxicity	kg 1,4-DCB	1.89×10^{-6}	7.21×10^{-7}	8.33×10^{-8}	9.83×10^{-8}	2.14×10^{-11}	8.59×10^{-8}	4.40×10^{-7}
Marine ecotoxicity	kg 1,4-DCB	2.89×10^{-7}	6.37×10^{-7}	6.95×10^{-8}	8.30×10^{-8}	1.88×10^{-11}	7.29×10^{-8}	9.31×10^{-7}
Carcinogenic toxicity hum.	kg 1,4-DCB	1.08×10^{-7}	5.88×10^{-6}	1.90×10^{-8}	7.71×10^{-8}	2.99×10^{-9}	5.20×10^{-8}	1.08×10^{-5}
Non-carcinogenic toxicity hum.	kg 1,4-DCB	2.17×10^{-9}	3.41×10^{-8}	1.65×10^{-9}	4.02×10^{-9}	3.65×10^{-13}	6.07×10^{-10}	-2.63×10^{-8}
Land use	m^2	1.21×10^{-4}	7.43×10^{-5}	3.81×10^{-5}	1.05×10^{-6}	-1.37×10^{-9}	3.97×10^{-5}	2.69×10^{-4}
Mineral scarcity	kg Cu equiv	1.69×10^{-10}	9.78×10^{-11}	1.35×10^{-10}	3.12×10^{-12}	3.30×10^{-15}	2.91×10^{-11}	3.69×10^{-10}
Fossil scarcity	kg oil equiv	2.03×10^{-6}	1.42×10^{-6}	3.64×10^{-7}	1.60×10^{-8}	5.22×10^{-12}	3.24×10^{-5}	5.46×10^{-5}
Water consumption	m^3	1.17×10^{-4}	2.00×10^{-3}	2.19×10^{-5}	2.43×10^{-4}	8.80×10^{-8}	8.43×10^{-4}	1.19×10^{0}

Table S70. Component-wise environmental impact assessment results in the phenibut manufacturing process considering continuous-flow method. KBr, NaOCl, THF, TEMPO, TBAF, and B.A. represent potassium bromide, sodium hypochlorite, tetrahydrofuran, 2,2,6,6-tetramethylpiperidin-1-yl, tetra-n-butylammonium fluoride and benzyl alcohol, respectively.

Impact category	Unit	Benzal-dehyde	Nitro methane	KBr	NaOCl	THF	TEMPO	TBAF	B.A.
Global warming	kg CO_2 equiv	1.64×10^{-3}	1.85×10^{-3}	9.34×10^{-5}	4.80×10^{-4}	6.23×10^{-4}	3.95×10^{-5}	3.33×10^{-5}	5.10×10^{-4}
Ozone depletion	kg CFC11 equiv	1.28×10^{-8}	7.65×10^{-9}	5.60×10^{-10}	9.24×10^{-9}	3.73×10^{-9}	1.28×10^{-10}	1.88×10^{-10}	1.99×10^{-9}
Ionizing radiation	kBq Co-60 equiv	4.10×10^{-7}	2.73×10^{-7}	1.14×10^{-8}	1.87×10^{-7}	9.94×10^{-8}	2.76×10^{-9}	1.56×10^{-9}	5.97×10^{-9}
Ozone form. hum.	kg NO_x equiv	1.45×10^{-7}	1.46×10^{-7}	9.72×10^{-9}	5.78×10^{-8}	6.80×10^{-8}	3.73×10^{-9}	5.42×10^{-9}	4.79×10^{-8}
Particulate matter	kg PM2.5 equiv	6.87×10^{-8}	9.63×10^{-8}	5.79×10^{-9}	3.84×10^{-8}	4.05×10^{-8}	1.73×10^{-9}	4.04×10^{-9}	2.23×10^{-8}
Ozone form. ter.	kg NO_x equiv	1.78×10^{-7}	1.73×10^{-7}	1.14×10^{-8}	6.77×10^{-8}	8.16×10^{-8}	4.63×10^{-9}	6.42×10^{-9}	5.83×10^{-8}
Ter. acidification	kg SO_2 equiv	1.14×10^{-7}	1.40×10^{-7}	7.69×10^{-9}	4.70×10^{-8}	5.49×10^{-8}	2.63×10^{-9}	7.38×10^{-9}	3.80×10^{-8}
Water eutrophication	kg P equiv	9.71×10^{-7}	8.53×10^{-7}	4.48×10^{-8}	4.42×10^{-7}	3.33×10^{-7}	7.01×10^{-8}	1.69×10^{-8}	1.59×10^{-7}
Marine eutrophication	kg N equiv	1.01×10^{-8}	1.23×10^{-7}	4.48×10^{-10}	6.27×10^{-9}	3.25×10^{-9}	2.17×10^{-8}	1.20×10^{-10}	7.52×10^{-10}
Terrestrial ecotoxicity	kg 1,4-DCB	3.17×10^{-8}	1.14×10^{-8}	1.07×10^{-9}	7.02×10^{-9}	1.00×10^{-8}	2.33×10^{-10}	9.63×10^{-9}	7.83×10^{-8}
Water ecotoxicity	kg 1,4-DCB	7.89×10^{-8}	7.72×10^{-8}	4.95×10^{-9}	4.13×10^{-8}	3.27×10^{-8}	3.29×10^{-9}	5.91×10^{-8}	2.92×10^{-7}
Marine ecotoxicity	kg 1,4-DCB	6.39×10^{-8}	6.58×10^{-8}	4.28×10^{-9}	3.52×10^{-8}	2.86×10^{-8}	1.32×10^{-9}	4.95×10^{-8}	2.36×10^{-7}
Carcinogenic toxicity hum.	kg 1,4-DCB	2.92×10^{-8}	2.04×10^{-8}	1.92×10^{-9}	1.82×10^{-8}	5.98×10^{-8}	3.20×10^{-8}	4.09×10^{-8}	2.45×10^{-7}
Non-carcinogenic toxicity hum.	kg 1,4-DCB	5.76×10^{-10}	3.85×10^{-10}	3.02×10^{-11}	2.84×10^{-10}	1.78×10^{-10}	1.77×10^{-11}	2.36×10^{-9}	1.16×10^{-8}
Land use	m^2	3.91×10^{-5}	3.19×10^{-5}	2.32×10^{-6}	1.60×10^{-5}	1.60×10^{-5}	5.68×10^{-7}	8.58×10^{-7}	6.52×10^{-6}
Mineral scarcity	kg Cu equiv	4.15×10^{-11}	2.67×10^{-11}	2.29×10^{-12}	1.93×10^{-11}	1.27×10^{-11}	7.07×10^{-13}	2.08×10^{-12}	9.25×10^{-12}
Fossil scarcity	kg oil equiv	7.97×10^{-7}	6.63×10^{-7}	2.70×10^{-8}	1.25×10^{-7}	2.28×10^{-7}	1.93×10^{-8}	2.10×10^{-8}	2.15×10^{-7}
Water consumption	m^3	4.39×10^{-5}	8.82×10^{-5}	7.04×10^{-7}	1.46×10^{-5}	3.38×10^{-5}	9.09×10^{-7}	4.03×10^{-5}	4.60×10^{-4}

Table S71. Component-wise environmental impact assessment results in the phenibut manufacturing process considering continuous-flow method. $NaHCO_3$, MCA, LiOH, and Elec. represent sodium bicarbonate, methyl cyanoacetate, lithium hydroxide and electricity, respectively.

Impact category	Unit	H_2O	$NaHCO_3$	MCA	LiOH	H_2	Toluene	Elec.
Global warming	kg CO_2 equiv	3.17×10^{-6}	3.87×10^{-5}	3.82×10^{-4}	3.97×10^{-6}	9.42×10^{-6}	1.12×10^{-2}	4.07×10^{-3}
Ozone depletion	kg CFC11 equiv	-1.66×10^{-11}	1.97×10^{-10}	5.07×10^{-9}	3.26×10^{-11}	3.42×10^{-13}	3.25×10^{-8}	1.51×10^{-8}
Ionizing radiation	kBq Co-60 equiv	1.26×10^{-10}	3.36×10^{-9}	3.68×10^{-8}	2.29×10^{-10}	0	6.57×10^{-8}	1.12×10^{-7}
Ozone form. hum.	kg NO_x equiv	3.01×10^{-10}	5.33×10^{-9}	5.83×10^{-8}	1.20×10^{-9}	8.30×10^{-11}	9.39×10^{-7}	2.05×10^{-7}
Particulate matter	kg PM2.5 equiv	1.21×10^{-10}	4.07×10^{-9}	3.16×10^{-8}	2.74×10^{-10}	1.48×10^{-10}	3.54×10^{-7}	6.05×10^{-8}
Ozone form. ter.	kg NO_x equiv	3.83×10^{-10}	6.24×10^{-9}	7.18×10^{-8}	1.42×10^{-9}	1.00×10^{-10}	1.14×10^{-6}	2.41×10^{-7}
Ter. acidification	kg SO_2 equiv	1.93×10^{-10}	8.42×10^{-9}	5.21×10^{-8}	3.58×10^{-10}	3.17×10^{-10}	7.04×10^{-7}	1.18×10^{-7}
Water eutrophication	kg P equiv	3.12×10^{-10}	3.06×10^{-8}	2.57×10^{-7}	8.95×10^{-10}	7.37×10^{-12}	5.43×10^{-8}	4.43×10^{-8}
Marine eutrophication	kg N equiv	2.14×10^{-10}	2.83×10^{-10}	1.27×10^{-9}	9.49×10^{-12}	1.07×10^{-12}	7.37×10^{-9}	1.49×10^{-8}
Terrestrial ecotoxicity	kg 1,4-DCB	4.85×10^{-10}	1.23×10^{-9}	2.07×10^{-8}	7.41×10^{-11}	3.19×10^{-13}	2.04×10^{-7}	5.87×10^{-8}
Water ecotoxicity	kg 1,4-DCB	2.41×10^{-9}	3.14×10^{-9}	2.26×10^{-8}	1.90×10^{-10}	5.34×10^{-13}	2.36×10^{-7}	8.74×10^{-9}
Marine ecotoxicity	kg 1,4-DCB	2.12×10^{-9}	2.84×10^{-9}	2.11×10^{-8}	1.72×10^{-10}	4.10×10^{-13}	3.32×10^{-7}	1.85×10^{-8}
Carcinogenic toxicity hum.	kg 1,4-DCB	3.37×10^{-7}	1.11×10^{-9}	8.12×10^{-9}	1.50×10^{-10}	4.98×10^{-11}	1.26×10^{-6}	2.14×10^{-7}
Non-carcinogenic toxicity hum.	kg 1,4-DCB	4.11×10^{-11}	2.75×10^{-11}	1.54×10^{-10}	2.16×10^{-12}	4.26×10^{-14}	6.50×10^{-8}	-5.23×10^{-10}
Land use	m^2	-1.56×10^{-7}	2.78×10^{-6}	1.92×10^{-5}	1.21×10^{-6}	0	8.48×10^{-6}	5.38×10^{-6}
Mineral scarcity	kg Cu equiv	3.72×10^{-13}	2.68×10^{-12}	1.01×10^{-11}	2.16×10^{-10}	0	1.60×10^{-11}	7.33×10^{-12}
Fossil scarcity	kg oil equiv	5.88×10^{-10}	8.28×10^{-9}	3.46×10^{-7}	1.32×10^{-9}	3.57×10^{-10}	1.01×10^{-5}	1.08×10^{-6}
Water consumption	m^3	9.67×10^{-6}	1.45×10^{-6}	2.18×10^{-5}	9.75×10^{-9}	4.64×10^{-9}	3.29×10^{-3}	2.30×10^{-2}

Table S72. Component-wise endpoint results in the phenibut manufacturing process considering batch method.

Damage category	Unit	Benzal-dehyde	Nitro methane	NaO Ac	MeOH	TsCl	Et_3N	THF	Thio.
Human health	DALY	1.51×10^{-8}	5.76×10^{-8}	1.90×10^{-9}	2.04×10^{-8}	3.40×10^{-8}	2.56×10^{-8}	2.21×10^{-7}	1.06×10^{-10}
Ecosystems	Species.yr	4.07×10^{-11}	1.40×10^{-10}	4.38×10^{-12}	5.25×10^{-11}	5.30×10^{-11}	6.80×10^{-11}	5.33×10^{-10}	2.29×10^{-13}
Resources	USD2013	1.60×10^{-3}	3.60×10^{-3}	9.04×10^{-5}	4.40×10^{-3}	5.12×10^{-3}	1.22×10^{-3}	2.64×10^{-2}	1.10×10^{-6}

Table S73. Component-wise endpoint results in the phenibut manufacturing process considering batch method.

Damage category	Unit	Diethyl malonate	$NaBH_4$	BF_3O Et_2	HCl	H_2O	Tolue ne	Elec.
Human health	DALY	1.00×10^{-8}	1.52×10^{-8}	3.35×10^{-9}	1.53×10^{-10}	1.75×10^{-13}	5.27×10^{-8}	2.53×10^{-7}
Ecosystems	Species.yr	2.36×10^{-11}	2.70×10^{-11}	6.19×10^{-12}	2.89×10^{-13}	1.72×10^{-16}	1.44×10^{-10}	7.03×10^{-10}
Resources	USD2013	6.47×10^{-4}	5.11×10^{-4}	1.16×10^{-4}	5.18×10^{-6}	1.34×10^{-9}	1.31×10^{-2}	1.73×10^{-2}

Table S74. Component-wise endpoint results in the phenibut manufacturing process considering continuous-flow method.

Damage category	Unit	Benzal-dehyde	Nitro methane	KBr	NaOCl	THF	TEMPO	TBAF	B.A.
Human health	DALY	2.70×10^{-9}	3.35×10^{-9}	1.80×10^{-10}	1.09×10^{-9}	1.30×10^{-9}	6.65×10^{-11}	1.14×10^{-10}	9.16×10^{-10}
Ecosystems	Species.yr	7.26×10^{-12}	7.94×10^{-12}	4.02×10^{-13}	2.41×10^{-12}	3.12×10^{-12}	1.86×10^{-13}	1.92×10^{-13}	2.02×10^{-12}
Resources	USD2013	2.85×10^{-4}	1.94×10^{-4}	7.66×10^{-6}	2.71×10^{-5}	6.42×10^{-5}	6.83×10^{-6}	7.54×10^{-6}	8.34×10^{-5}

Table S75. Component-wise endpoint results in the phenibut manufacturing process considering continuous-flow method.

Damage category	Unit	H_2O	$NaHCO_3$	MCA	LiOH	H_2	Toluene	Elec.
Human health	DALY	1.97×10^{-11}	1.04×10^{-10}	9.07×10^{-10}	8.12×10^{-12}	1.10×10^{-11}	1.70×10^{-8}	5.03×10^{-9}
Ecosystems	Species.yr	1.94×10^{-14}	2.53×10^{-13}	2.26×10^{-12}	3.75×10^{-14}	2.92×10^{-14}	4.47×10^{-11}	1.40×10^{-11}
Resources	USD2013	1.51×10^{-7}	1.75×10^{-6}	1.41×10^{-4}	6.50×10^{-6}	1.29×10^{-6}	4.55×10^{-3}	3.43×10^{-4}

11. References for Supporting Information

[1] Aspen Tech., Aspen Plus 11.1 User Guide, https://esupport.aspentech.com/FileAttachment?id=a0g0B00000GfK2z (accessed 08/09/2024)
